# Distinct Characteristic Binding Modes of Benzofuran Core Inhibitors to Diverse Genotypes of Hepatitis C Virus NS5B Polymerase: A Molecular Simulation Study

**DOI:** 10.3390/ijms25158028

**Published:** 2024-07-23

**Authors:** Di Han, Fang Zhao, Yifan Chen, Yiwei Xue, Ke Bao, Yuxiao Chang, Jiarui Lu, Meiting Wang, Taigang Liu, Qinghe Gao, Wei Cui, Yongtao Xu

**Affiliations:** 1 School of Medical Engineering, Xinxiang Medical University, Xinxiang 453003, China; zffff0213@163.com (F.Z.); 18236137338@163.com (Y.C.); xyw15896742780@163.com (Y.X.); 18790570138@163.com (K.B.); cyx19980529@163.com (Y.C.); lujiarui@xxmu.edu.cn (J.L.); wangmeitingbetter@163.com (M.W.); liuttgg@163.com (T.L.); 2 Henan International Joint Laboratory of Neural Information Analysis and Drug Intelligent Design, Xinxiang 453003, China; 3 Xinxiang Key Laboratory of Biomedical Information Research, Xinxiang 453003, China; 4 School of Pharmacy, Xinxiang Medical University, Xinxiang 453003, China; gao_qinghe@xxmu.edu.cn; 5 School of Chemical Sciences, University of Chinese Academy of Sciences, No. 19A, YuQuan Road, Beijing 100049, China; cuiwei@ucas.ac.cn

**Keywords:** benzofuran inhibitor, HCV NS5B polymerase, binding mechanism, molecular dynamics simulation, ASMD simulation

## Abstract

The benzofuran core inhibitors **HCV-796**, **BMS-929075**, **MK-8876**, compound **2**, and compound **9B** exhibit good pan-genotypic activity against various genotypes of NS5B polymerase. To elucidate their mechanism of action, multiple molecular simulation methods were used to investigate the complex systems of these inhibitors binding to GT**1a**, **1b**, **2a**, and **2b** NS5B polymerases. The calculation results indicated that these five inhibitors can not only interact with the residues in the palm II subdomain of NS5B polymerase, but also with the residues in the palm I subdomain or the palm I/III overlap region. Interestingly, the binding of inhibitors with longer substituents at the C5 position (**BMS-929075**, **MK-8876**, compound **2**, and compound **9B**) to the GT**1a** and **2b** NS5B polymerases exhibits different binding patterns compared to the binding to the GT**1b** and **2a** NS5B polymerases. The interactions between the para-fluorophenyl groups at the C2 positions of the inhibitors and the residues at the binding pockets, together with the interactions between the substituents at the C5 positions and the residues at the reverse β-fold (residues 441–456), play a key role in recognition and the induction of the binding. The relevant studies could provide valuable information for further research and development of novel anti-HCV benzofuran core pan-genotypic inhibitors.

## 1. Introduction

Hepatitis C virus (HCV) belongs to the Flaviviridae family and is the causative agent of hepatitis C [1,2]. HCV is a single-stranded, positive-stranded RNA virus, which is mainly transmitted through blood, mother-to-child, and sexual transmission. HCV infection can lead to chronic hepatitis C, cirrhosis, hepatocellular carcinoma, and other liver diseases, posing a great threat to the lives and health of patients [3]. According to data released by the World Health Organization (WHO) in 2022 [4], approximately 58 million people worldwide are infected with HCV, with about 1.5 million new infections occurring per year. HCV infection has been a major global public health concern for many years, and unfortunately, there is currently no effective vaccine against HCV [2].

HCV has high genetic variability due to its high replication rate and lack of proofreading activity of the polymerase on which its RNA replication depends. There are two dimensions of genetic variation in HCV [5]: intra-host variability and inter-host variability. Intra-host variability is characterized by the distribution of HCV mutant genomes present in an infected individual; inter-host variability is characterized by the generation of different HCV genotypes and subtypes among the globally circulating viral strains, and these genotypes exhibit different geographic distributions [6]. Currently, eight major genotypes (genotypes 1-8, GT1-8) have been identified, most of which have multiple subtypes (a, b, c, etc.) [7]. HCV genetic diversity is closely related to the pathogenesis and the development of antiviral strategies [5].

Much progress has been made with direct-acting antivirals (DAAs) for HCV [8,9]. Combination therapy can treat patients infected with multiple genotypes of HCV, but pre-treatment regimen development and even the adjustment of regimens during treatment require viral genotyping testing for the patients, which is difficult to achieve in many economically underdeveloped countries and regions [10,11]. Pan-genotypic HCV DAAs can reduce the need for genotyping to guide treatment decisions, reduce costs, and simplify procurement and supply chains, and are crucial for the treatment of hepatitis C patients in the many less-developed countries and regions where genotyping testing is not available [12]. Therefore, the development of novel pan-genotypic anti-HCV drugs is of great value and plays a significant role in achieving the WHO’s goal of eliminating the public health hazards of viral hepatitis.

At present, the main direct anti-HCV agents include NS3/4A inhibitors, NS5A inhibitors, and NS5B inhibitors [13]. Due to the lack of expression of enzymes with similar functions to NS5B polymerase in the human body [14], inhibitors targeting NS5B polymerase are less likely to induce host cellular toxicity. Moreover, this polymerase is crucial for the replication of viral RNA, making it an important target for anti-HCV drug development [15]. The structure of NS5B polymerase is similar to that of a human right hand, containing characteristic thumb (thumb I and II), palm (palm I, II, and III), and finger subdomains [2]. Among them, the palm subdomains are relatively well conserved, and NS5B polymerase inhibitors targeting this site generally have good pan-genotypic activity [16].

**Figure 1:**
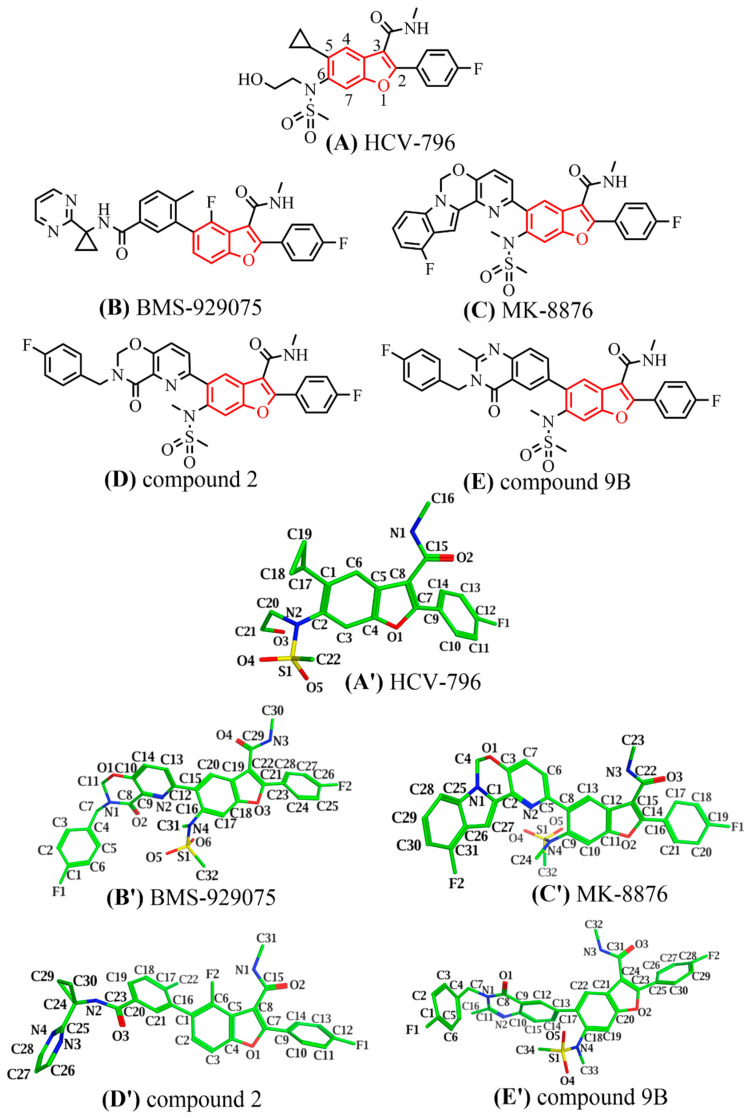
Chemical structures and 3D structures with atom labels of **HCV-796** (5-cyclopropyl-2-(4-fluorophenyl)-6-[(2-hydroxyethyl)(methyldioxo-λ6-sulfanyl)amino]-N-methyl-1-benzofuran-3-carboxamide), **BMS-929075** (3-[4-fluoro-2-(4-fluorophenyl)-3-[(methylamino)carbonyl]-1-benzofuran-5-yl]-4-methyl-N-(pyrimidin-2-ylcyclopropyl)benzamide), **MK-8876** (2-(4-fluorophenyl)-5-(11-fluoropyrido[2′,3′:5,6][1,3]oxazino[3,4-a]indol-2-yl)-N-methyl-6-[methyl(methyldioxo-λ6-sulfanyl)amino]-1-benzofuran-3-carboxamide), compound **2** (2-(4-fluorophenyl)-5-{3-[(4-fluorophenyl)methyl]-4-oxo-3,4-dihydro-2H-pyrido[2,3-e][1,3]oxazin-6-yl}-N-methyl-6-[methyl(methyldioxo-λ6-sulfanyl)amino]-1-benzofuran-3-carboxamide), and compound **9B** (2-(4-fluorophenyl)-5-{3-[(4-fluorophenyl)methyl]-2-methyl-4-oxoquinazolin-6-yl}-N-methyl-6-[methyl(methyldioxo-λ6-sulfanyl)amino]-1-benzofuran-3-carboxamide).

In recent years, a series of benzofuran inhibitors capable of acting on the palm II subdomain of HCV NS5B polymerase have been reported (Figure 1). These inhibitors share the same 2-(4-fluorophenyl)-N-methyl-1-benzofuran-3-carboxamide substructure and exhibit effective inhibitory activity against multiple genotypes of HCV NS5B polymerase. Specifically, the compound **HCV-796** [17] (Figure 1A), jointly developed by Wyeth Research and ViroPharma Incorporated, is the first non-nucleoside inhibitor to enter Phase II clinical trials [18]. This inhibitor showed good activity and a good antiviral profile (GT**1a**, **1b**, **2a**, **3a**, and **4a**), but the clinical trial was terminated due to its hepatotoxicity [19]. In 2017, Kap-Sun Yeung and coworkers from Bristol Myers Squibb reported the 4-fluorobenzofuran core compound **BMS-929075** [20] (Figure 1B) with pan-genotypic antiviral activity. Except for GT2 HCV NS5B polymerase, this compound showed effective inhibition of GT1-6 NS5B and high oral bioavailability in three preclinical animal models. In the same year, a research team from Merck & Co. Inc. (Rahway, NJ, USA) obtained a series of novel 5-aryl benzofuran compounds by modifying the C5 position of the benzofuran core, from which the clinical candidate **MK-8876** [16] was discovered (Figure 1C). This compound can simultaneously interact with both the palm I and palm II subdomains and effectively inhibit various genotypes of HCV NS5B polymerase (GT**1a**, **1b**, **2a**, **2b**, **3a**, and **4a**) with EC_50_ values in the range of several nanomoles [21]. However, the special tetracyclic structure of **MK-8876** makes it have low solubility, so researchers tried to solve this problem by breaking the planarity of the tetracyclic ring. Dong Xiao et al. tried to open the tetracyclic ring of **MK-8876** and introduced a carbonyl group, resulting in compound **2** (Figure 1D). Subsequently, further optimization of compound **2** yielded compound **9B** (Figure 1E) containing a quinazolinone bicyclic ring. This bicyclic compound showed EC_50_ values of less than 5 nM against several genotypes of HCV NS5B polymerase (GT**1a**, **1b**, **2a**, **2b**, **3a**, and **4a**), and its FASSIF solubility was superior to that of the tetracyclic compound **MK-8876** [22].

In view of the effective inhibitory activity of the above inhibitors on multiple genotypes of HCV NS5B polymerase, benzofuran core compounds have great potential to become anti-HCV drugs. However, the detailed mechanism of action of these inhibitors is still unclear. Therefore, in this study, the binding mechanism of the above benzofuran core pan-genotypic HCV NS5B polymerase (GT**1a**, **1b**, **2a**, and **2b**) inhibitors was investigated through a combination of molecular simulation methods, such as molecular docking, molecular dynamics (MD) simulations, MM/GBSA energy calculations, and ASMD simulations. The research results could provide valuable information for the further design and optimization of this series of inhibitors.

## 2. Results and Discussion

### 2.1. Stability of the NS5B/Inhibitor Complexes during the MD Simulations

**Figure 2:**
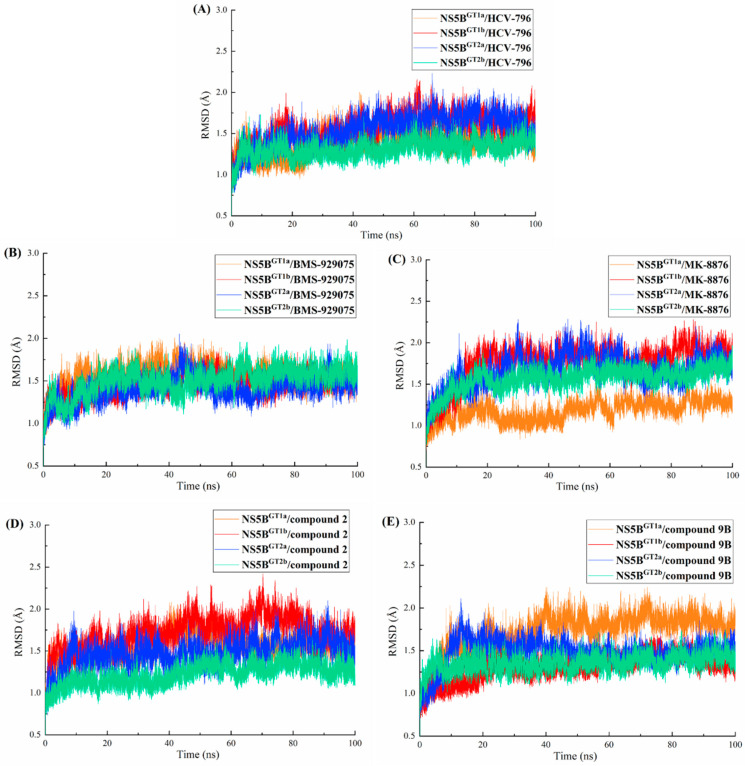
The root-mean-square deviations (RMSDs) of the complex systems. (**A**) The systems of the four genotypes of NS5B polymerase with **HCV-796**. (**B**) The systems of the four genotypes of NS5B polymerase with **BMS-929075**. (**C**) The systems of the four genotypes of NS5B polymerase with **MK-8876**. (**D**) The systems of the four genotypes of NS5B polymerase with compound **2**. (**E**) The systems of the four genotypes of NS5B polymerase with compound **9B**.

MD simulations (lasting 100 ns) were performed for each of the twenty complex systems formed by binding the five inhibitors with the four genotypes of NS5B polymerase. To evaluate whether the simulation of each system reached equilibrium, the root-mean-square deviations (RMSDs) of the receptor backbone atoms and ligand were calculated relative to their respective initial structures (Figure 2 and Figure S1). As can be seen from the figures, all systems reached a stable state after 40 ns in the MD simulations. Some small fluctuations were still within the acceptable scale. Therefore, it is reasonable and reliable to perform the following binding free energy calculations and free energy decomposition analyses based on the trajectories in the last 20 ns.

### 2.2. Binding Free Energies Predicted by MM/GBSA Method

Table 1 lists the binding free energies and the individual energy terms for each complex system predicted by the MM/GBSA method. From the table, it can be seen that the binding free energy values of each complex system range from −47.97 kcal/mol to −84.98 kcal/mol, indicating that these five inhibitors should have different degrees of inhibition against the four genotypes of NS5B polymerase. Comparing the contribution values of each energy term in the table, it is obvious that the van der Waals contribution ($∆E_{\mathrm{vdw}}$) is the most important component of the binding free energy of each system, followed by the electrostatic contribution ($∆E_{\mathrm{ele}}$). This indicates that the intermolecular van der Waals interactions play a key role in the binding of these inhibitors to the four genotypes of NS5B polymerase.

Since the polar contribution of desolvation ($∆G_{\mathrm{GB}}$) will offset some of the electrostatic contribution, all the net electrostatic contributions ($∆E_{\mathrm{ele}}+∆G_{\mathrm{GB}}$) in these systems are more than 3.23 kcal/mol, indicating that the net electrostatic contribution in the solution environment is unfavorable for the binding of such inhibitors with NS5B polymerase. In addition, the desolvation energies $∆G_{\mathrm{sol}}$ in the complex systems are all greater than 24.70 kcal/mol, suggesting that solvation is also detrimental to the binding of such inhibitors to NS5B polymerase. Thus, van der Waals interactions should be the main factor promoting the stable binding of these benzofuran core inhibitors to the four genotypes of NS5B polymerase.

**Figure 3:**
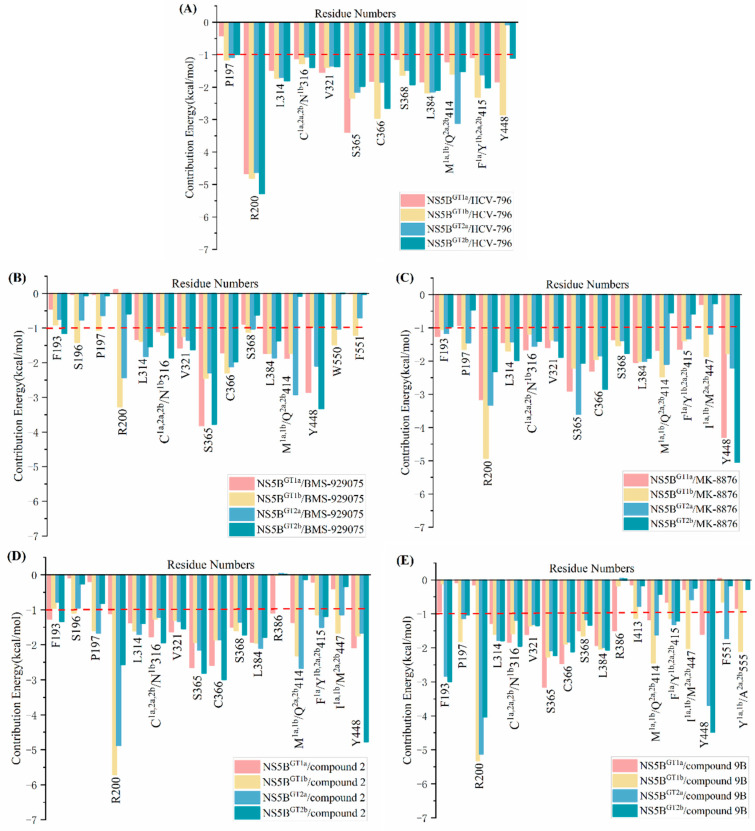
Residues that contribute significantly to the binding free energy of each system (<−1.00 kcal/mol) according to the MM/GBSA decomposition analyses. (**A**) The systems of the four genotypes of NS5B polymerase with **HCV-796**. (**B**) The systems of the four genotypes of NS5B polymerase with **BMS-929075**. (**C**) The systems of the four genotypes of NS5B polymerase with **MK-8876**. (**D**) The systems of the four genotypes of NS5B polymerase with compound **2**. (**E**) The systems of the four genotypes of NS5B polymerase with compound **9B**.

To gain insights into the details of the interaction between these five inhibitors and the four genotypes of NS5B polymerase, the per-residue energy decomposition of binding free energy was calculated using MM/GBSA method. The residues that contribute significantly to the binding free energy of each system (<−1.00 kcal/mol) are shown in Figure 3. In addition, as an important type of protein–drug interaction, intermolecular hydrogen bonds were also calculated based on the MD simulation trajectories in the last 20 ns, and the results are listed in Table 2. A hydrogen bond is defined if the donor–acceptor distance is less than 3.00 Å and the donor–donor H-acceptor angle is >135.00°. It is generally accepted that an occupancy of more than 20% in the MD trajectories indicates that an effective hydrogen bond has been formed [23,24]. The relevant detailed discussion will be provided later.

### 2.3. The Binding Modes of the Benzofuran Core Inhibitors with NS5B Polymerases

#### 2.3.1. The Binding Modes of **HCV-796** Binding to NS5B Polymerases

**Figure 4:**
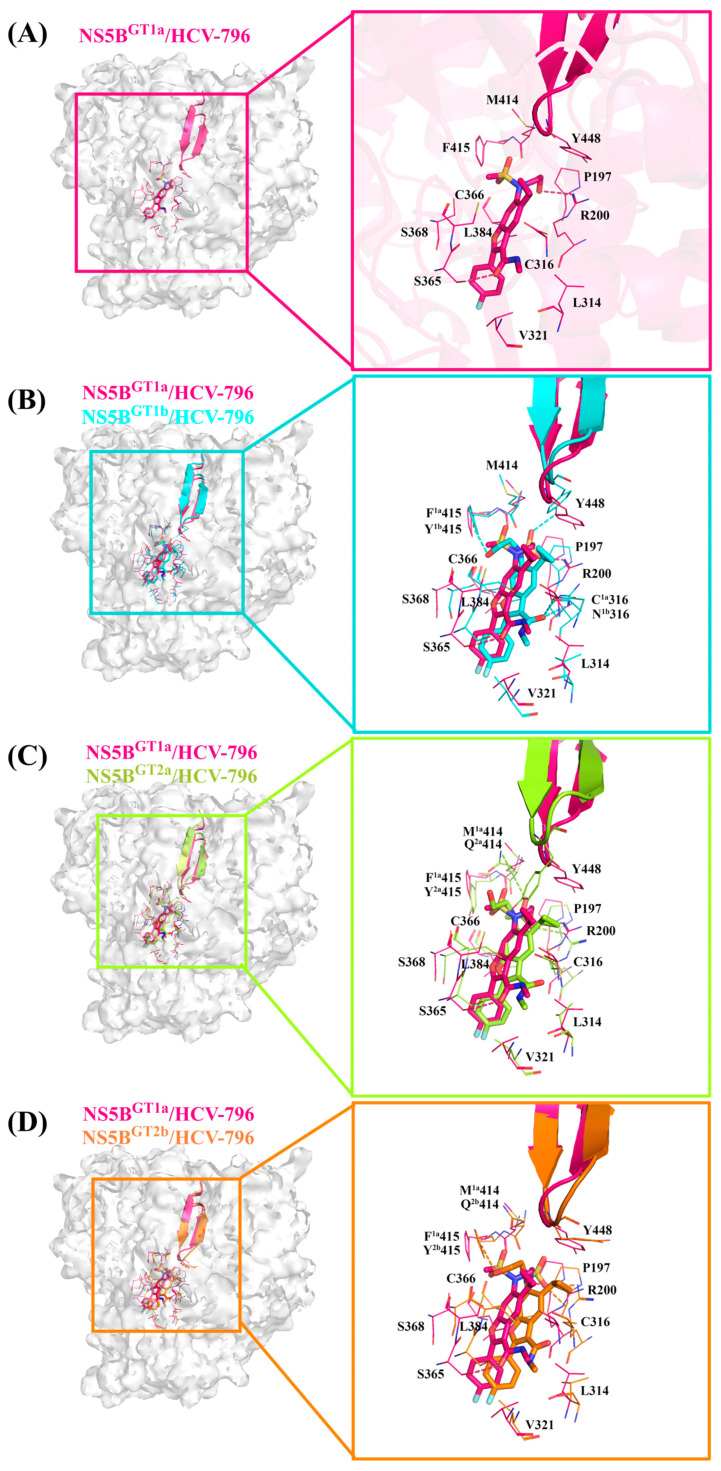
Binding modes of **HCV-796** with four genotypes of NS5B polymerase. The dashed lines represent hydrogen bonds. The structure shown represents the last frame of the simulation after reaching a steady state. (**A**) Binding modes of NS5B^GT1a^/**HCV-796**. (**B**) Binding modes of NS5B^GT1a^/**HCV-796** and NS5B^GT1b^/**HCV-796**. (**C**) Binding modes of NS5B^GT1a^/**HCV-796** and NS5B^GT2a^/**HCV-796**. (**D**) Binding modes of NS5B^GT1a^/**HCV-796** and NS5B^GT2b^/**HCV-796**.

According to the classification results of the palm subdomains of NS5B polymerase by Maria Letizia Barreca et al. [25], the palm II subdomain contains residues such as Phe193, Pro197, and Arg200. The palm I subdomain contains residues Phe193, Pro197, Arg200, Val228, Asn291, Cys^1a,2a,2b^/Asn^1b^316, Gly317, Asp318, Cys366, Ser368, Leu384, Met^1a,1b^/Gln^2a,2b^414, Phe^1a^/Tyr^1b,2a,2b^415, Glu^1a,2a,2b^/Gln^1b^446, Ile^1a,1b^/Met^2a,2b^447, Tyr448, Gly449, and Ser^1a,1b^/Gly^2a,2b^556; the palm III subdomain contains residues Tyr195, Pro197, Arg200, Leu384, Met^1a,1b^/Gln^2a,2b^414, Phe^1a^/Tyr^1b,2a,2b^415, Cys^1a,2a,2b^/Asn^1b^316, Ile^1a,1b^/Met^2a,2b^447, Tyr452, Glu^1a,2a,2b^/Gln^1b^446, Trp550, and Phe551. There is some overlap among these regions. From the key residues that were identified by binding free energy decomposition (Figure 3A), **HCV-796** can establish key interactions with residues Arg200, Leu314, Cys^1a,2a,2b^/Asn^1b^316, Val321, Ser365, Cys366, Ser368, Leu384, Met^1a,1b^/Gln^2a,2b^414, and Phe^1a^/Tyr^1b,2a,2b^415 after binding with the four genotypes of NS5B polymerase. The contribution values of these residues to the binding free energy of the corresponding system are all less than −1.00 kcal/mol. In addition to establishing extensive interactions with residues in the palm II subdomain, this inhibitor can also interact with Ser368 in the palm I subdomain and the residues Cys^1a,2a,2b^/Asn^1b^316 and Leu384 in its overlap region with the palm III subdomain. In the NS5B^GT1a^/**HCV-796** system (Figure 4A), the benzofuran core of **HCV-796** can interact with the surrounding residues Arg200, Cys316, Ser365, Cys366, Ser368 and Leu384. Coincidentally, these key residues near the inhibitor core are all polar residues except Leu384, and polar solvation weakens the binding strength of the inhibitor to these residues, which is confirmed by the decomposition of the energy terms of the key residues (Figure S2A). This suggests that the future structural optimization of scaffolds based on such inhibitors should consider the fact that there are multiple polar residues around. In addition, the methyl amide group at the C3 position and the sulfonamide group at the C6 position of the inhibitor benzofuran core establish hydrophobic interactions with the surrounding residues Leu314, Val321, Met414, and Phe415 (Figure 4A and Figure S2A). Moreover, the cyclopropyl group at the C5 position and the sulfonamide group at the C6 position also interact with the nearby Tyr448 (−1.84 kcal/mol). The hydrogen bond calculations (Table 2) show that the oxygen atom of the C3 formamide group of **HCV-796** is facing residue Ser365 and can form a strong hydrogen bond with it [OG-HG (Ser365) ··· O2 (**HCV-796**)]. The oxygen atom on the C6 hydroxyl group is able to form a weak hydrogen bond with residue Arg200 [NH1-HH12 (Arg200) ··· O3 (**HCV-796**)].

In order to compare and analyze the interactions between the key residues in the binding pockets and **HCV-796** after binding with different genotypes of NS5B polymerase, the differences between the contribution values of key residues to binding free energy in the other three systems and the corresponding contribution values of key residues to the binding free energy in the NS5B^GT1a^/**HCV-796** system were calculated. The results are shown in Figure S3A. Taking ±0.5 kcal/mol as the threshold, the contributions of the five key residues to the binding free energy in the NS5B^GT1b^/**HCV-796** system are significantly different from those in the NS5B^GT1a^/**HCV-796** system (Pro197, Ser365, Cys366, Tyr^1b^415, and Tyr448). Compared to binding to NS5B^GT1a^ polymerase, **HCV-796** binds to NS5B^GT1b^ polymerase to form a new hydrogen bonding network (Table 2 and Figure 4B). The MD simulations showed that the inhibitor can form a weak hydrogen bond with residues Arg200 and Asn1b316 ([NH1-HH12 (Arg200) ··· O2 (**HCV-796**)] and [ND2-HD21 (Asn316) ··· O2 (**HCV-796**)]), and a strong hydrogen bond with residues Tyr1b415 and Tyr448 ([OH-HH (Tyr415) ··· O3 (**HCV-796**)] and [OH-HH (Tyr448) ··· O4 (**HCV-796**)]). This is mainly due to the rotation of the N-methylmethanamide at the C3 position and the N-(2-hydroxyethyl)methanesulfonamide at the C6 position of the benzofuran core (both of them rotate by almost 180°). Although **HCV-796** and Asn316 can form a hydrogen bond in the NS5B^GT1b^/**HCV-796** system, due to the close distance between the carbonyl oxygen of the residue side chain and the carbonyl oxygen of the inhibitor C3 substituent (Figure S4B), the repulsive force between these groups leads to a positive electrostatic contribution of the residue to the binding free energy (0.21 kcal/mol). Therefore, the mutation from Cys316 in NS5B^GT1a^ polymerase to Asn316 in NS5B^GT1b^ polymerase does not significantly increase the binding force of **HCV-796** to the residue at this position (Figure S2A,B). Unlike the above situation, the hydrogen bond formed with **HCV-796** after mutation from Phe415 in NS5B^GT1a^ polymerase to Tyr415 in NS5B^GT1b^ polymerase markedly enhances the strength of binding between the inhibitor and polymerase (Figures S2A,B and S3A). Compared with the situation in the NS5B^GT1a^/**HCV-796** system, the hydrogen bond formed by Tyr448 with the inhibitor also significantly enhances the binding strength (Figures S2A,B and S3A). This rotation also leads to the inability to form an effective hydrogen bond between the inhibitor and Ser365, resulting in a weaker contribution of this residue to the binding free energy of the NS5B^GT1b^/**HCV-796** system compared to its contribution to the binding free energy of the NS5B^GT1a^/**HCV-796** system (ΔΔG = 1.06 kcal/mol). Moreover, the rotation of the methylamide group at the C3 position brings its carbonyl oxygen closer to the sulfhydryl group of Cys366 (Figure S4C), resulting in enhanced electrostatic interactions between the inhibitor and this residue (Figure S2A,B), as well as an enhanced contribution of this residue to the binding free energy (Figure S3A). In addition, the distance between **HCV-796** and Pro197 is smaller in the NS5B^GT1b^/**HCV-796** system compared to that in the NS5B^GT1a^/**HCV-796** system (Figure S4A), and there is an enhancement of the van der Waals interactions between them (Figure S2A,B), as well as an enhancement of the contribution of this residue to the binding free energy (Figure S3A).

From the energetic difference spectra of the key residues (Figure S3A), it can be seen that compared to the corresponding residues in the NS5B^GT1a^/**HCV-796** system, five residues (Pro197, Ser365, Gln^2a^414, Tyr^2a^415, and Tyr448) in the NS5B^GT2a^/**HCV-796** system have significantly different contributions to the binding free energy. The superimposed simulated steady-state structures of NS5B^GT2a^/**HCV-796** and NS5B^GT1a^/**HCV-796** (Figure 4C) revealed that the N-methylmethanamide at the C3 position and the N-(2-hydroxyethyl)methanesulfonamide at the C6 position of the benzofuran core also rotate to a certain degree upon binding of **HCV-796** to NS5B^GT2a^ polymerase compared to its binding to NS5B^GT1a^ polymerase, and that the corresponding magnitude of the rotations is similar to that in the NS5B^GT1b^/**HCV-796** system. Compared to the NS5B^GT1a^/**HCV-796** system, these rotations also reconstruct the hydrogen bond network in the NS5B^GT2a^/**HCV-796** system, but the positions of the hydrogen bonds formed are different from those in the NS5B^GT1b^/**HCV-796** system ([NH2-HH21 (Arg200) ··· O5 (**HCV-796**)] and [NE2-HE22 (Gln414) ··· O4 (**HCV-796**)]). Meanwhile, the hydrogen bond formed between **HCV-796** and Gln^2a^414 significantly enhances their binding strength (Figures S2A,C and S3A). Compared with the situation in the NS5B^GT1a^/**HCV-796** system, the rotation of the C3 N-methylmethanamide also leads to a weakened interaction between the inhibitor and Ser365 (ΔΔG = 1.24 kcal/mol); the reason for this is the same as in the NS5B^GT1b^/**HCV-796** system. Elsewhere, the induced fit effect of **HCV-796** binding to NS5B^GT2a^ polymerase also results in enhanced interactions of the inhibitor with the residues Pro197 and Tyr^2a^415, and a weakened interaction with Tyr448 compared to binding to NS5B^GT1a^ polymerase. This is due to changes in the distances between the inhibitor and these residues after binding (Figure S4A,E,F).

For the NS5B^GT2b^/**HCV-796** system (Figure 4D), compared to the NS5B^GT1a^/**HCV-796** system, the N-methylmethanamide at the C3 position and the N-(2-hydroxyethyl)methanesulfonamide at the C6 position of the benzofuran core of the binding inhibitor also undergo similar rotations as in the NS5B^GT1b^/**HCV-796** and NS5B^GT2a^/**HCV-796** systems, and form two stronger hydrogen bonds to Arg200 ([NH2-HH22 (Arg200) ··· O5 (**HCV-796**)] and [NE-HE (Arg200) ··· O5 (**HCV-796**)]), as well as one weaker hydrogen bond to Tyr^2b^415 ([OH-HH (Tyr415) ··· O3 (**HCV-796**)]). This also enhances the interactions between the inhibitor and these two residues compared to the corresponding residues in the NS5B^GT1a^/**HCV-796** system (Figure S3A). The rotation of the N-methylmethanamide at the C3 position also leads to an attenuation of the interaction between this inhibitor and Ser365 (ΔΔG = 1.42 kcal/mol) compared to the situation in the NS5B^GT1a^/**HCV-796** system, for the same reason as in the NS5B^GT1b^/**HCV-796** and NS5B^GT2a^/**HCV-796** systems. At the same time, this rotation also leads to enhanced interactions between the inhibitor and residue Cys366 (Figures S3A and S4C), which is the same reason as in the NS5B^GT1b^/**HCV-796** system. In addition, the interactions of the inhibitor with Pro197 and Ser368 are enhanced, while the interaction with Tyr448 is weakened, all of which are caused by changes in the distances between the inhibitor and these residues (Figure S4A,D,F).

#### 2.3.2. The Binding Modes of **BMS-929075** with NS5B Polymerases

Structurally, **BMS-929075** is obtained by replacing the hydrogen atom at the C4 position, cyclopropyl group at the C5 position, and N-(2-hydroxyethyl)methanesulfonamide at the C6 position of the **HCV-796** benzofuran core with a fluorine atom, 4-methyl-N-(pyrimidin-2-ylcyclopropyl)benzamide, and a hydrogen atom, respectively. From the key residues identified by binding free energy decomposition (Figure 3B), it can be seen that **BMS-929075** can establish important interactions with residues Leu314, Cys^1a,2a,2b^/Asn^1b^316, Val321, Ser365, Cys366, and Leu384 after binding with the four genotypes of NS5B polymerase. Of these, Cys^1a,2a,2b^/Asn^1b^316 and Leu384 are located in the palm I/III overlap region, and the remaining residues are located in the palm II subdomain.

**Figure 5:**
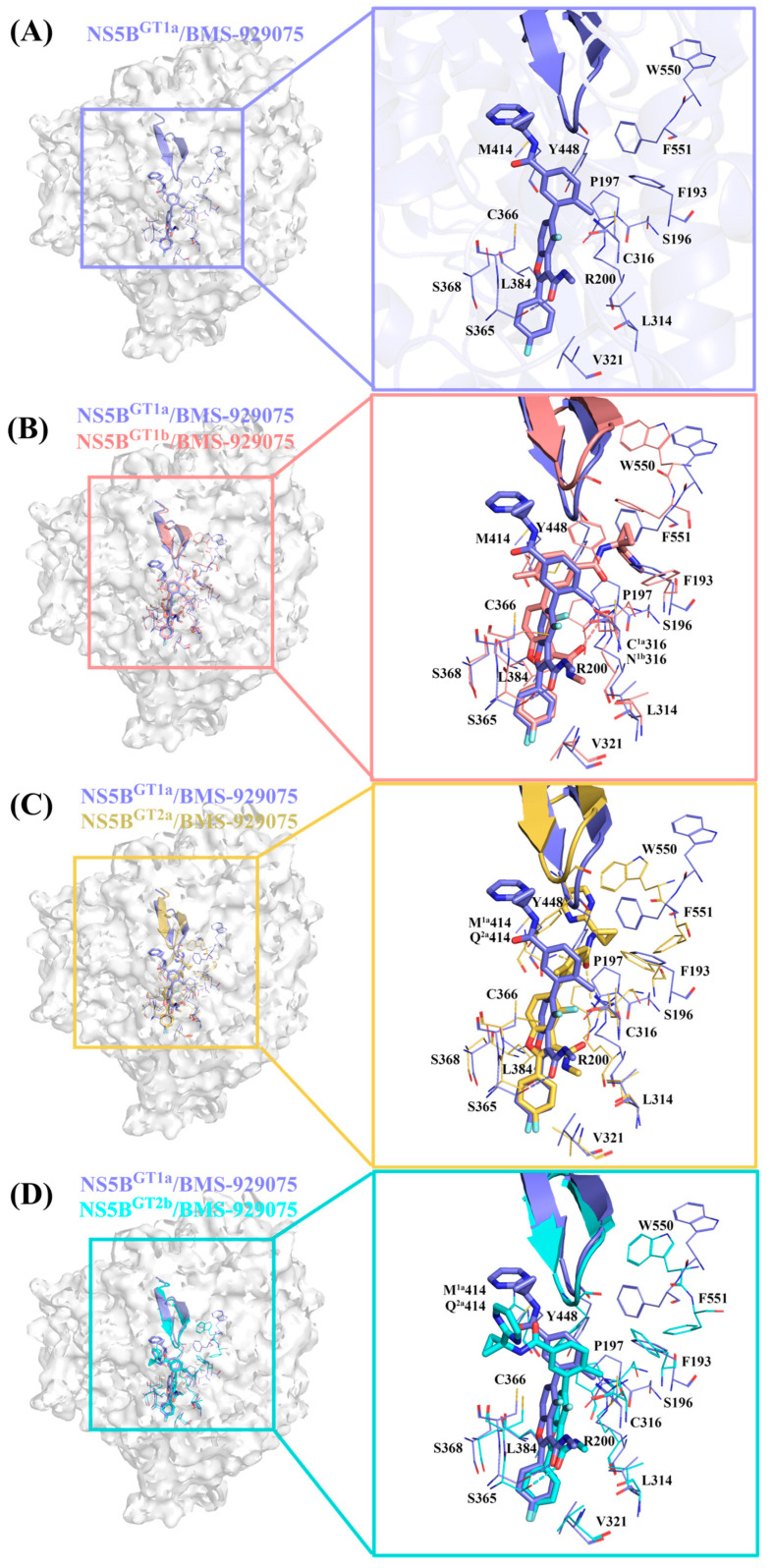
Binding modes of **BMS-929075** with four genotypes of NS5B polymerase. The structure shown represents the last frame of the simulation after reaching a steady state. (**A**) Binding modes of NS5B^GT1a^/**BMS-929075**. (**B**) Binding modes of NS5B^GT1a^/**BMS-929075** and NS5B^GT1b^/**BMS-929075**. (**C**) Binding modes of NS5B^GT1a^/**BMS-929075** and NS5B^GT2a^/**BMS-929075**. (**D**) Binding modes of NS5B^GT1a^/**BMS-929075** and NS5B^GT2b^/**BMS-929075**.

In the NS5B^GT1a^/**BMS-929075** system (Figure 5A), compared with the NS5B^GT1a^/**HCV-796** system (Figures S5 and S6A), the introduction of 4-methyl-N-(pyrimidin-2-ylcyclopropyl) benzamide at the C5 position of the **BMS-929075** benzofuran core significantly enhances its binding strength to the nearby residues Met414 (ΔΔG = −0.66 kcal/mol), Tyr448 (ΔΔG = −1.02 kcal/mol), and Gly449 (ΔΔG = −0.96 kcal/mol). Meanwhile, due to the absence of N-(2-hydroxyethyl)methanesulfonamide at the C6 position of **BMS-929075** compared to **HCV-796**, the binding force of this inhibitor with nearby residues Arg200 (ΔΔG = 4.77 kcal/mol) and Phe415 (ΔΔG = 0.55 kcal/mol) is weakened and cannot form hydrogen bonds with Arg200. However, the oxygen atom of the N-methylmethanamide at the C3 position of **BMS-929075** is still able to form a strong hydrogen bond with residue Ser365 (Table 2, [OG-HG (Ser365) ··· O2 (**BMS-929075**)]). Additionally, the interaction energy spectra of the NS5B^GT1a^/**BMS-929075** system and the NS5B^GT1a^/**HCV-796** system are similar (Figure 3A,B).

Compared to the NS5B^GT1a^/**BMS-929075** system, there are a total of eight residues (Ser196, Pro197, Arg200, Ser365, Cys366, Tyr448, Trp550, and Phe551) in the NS5B^GT1b^/**BMS-929075** system that contribute significantly to the binding free energy. The MD simulations indicated that the inhibitor can form a strong hydrogen bond with Arg200 ([NH1-HH12 (Arg200) ··· O2 (**BMS-929075**)]), effectively enhancing its binding strength (Table 2, Figure 5B and Figure S3B). This is mainly due to the rotation of the N-methylmethanamide at the C3 position and the 4-methyl-N-(pyrimidin-2-ylcyclopropyl)benzamide at the C5 position of **BMS-929075**. The N-methylmethanamide is rotated by almost 180°, which is the same as in the NS5B^GT1b^/**HCV-796** system. Meanwhile, this rotation enhances the interaction between the inhibitor and Cys366 (Figures S3B and S7F). This rotation, however, results in the inability of **BMS-929075** to form an effective hydrogen bond with Ser365, and thus the binding free energy contribution of Ser365 to the NS5B^GT1b^/**BMS-929075** system is lower compared to that to the NS5B^GT1a^/**BMS-929075** system (ΔΔG = 1.37 kcal/mol). In addition, the rotation of 4-methyl-N-(pyrimidin-2-ylcyclopropyl) benzamide at the C5 position of **BMS-929075** reduces its distances from the nearby residues Ser196, Pro197, Trp550, and Phe551 (Figure S7B,C,I,J), significantly enhancing the binding strength with these residues (Figure S3B), while the increase in distance from Tyr448 (Figure S7H) weakens the binding strength with this residue (ΔΔG = 1.89 kcal/mol).

Notably, a comparative analysis of the binding modes of NS5B^GT1b^/**BMS-929075** and NS5B^GT1a^/**BMS-929075** revealed that there is a significant difference in the orientation of the substituent at the C5 position of the **BMS-929075** benzofuran core upon binding to the GT1b and 1a NS5B polymerases. Calculations by CAVER 3.0 software showed that the substituent at the C5 position is oriented toward the cavity where residue Phe551 is located in NS5B^GT1b^ polymerase (Figure S8B) and toward the cavity where residue Arg394 is located in NS5B^GT1a^ polymerase (Figure S8A). These two cavities are separated by a reverse β-fold (residues 441–456).

Compared with binding to NS5B^GT1a^ polymerase, **BMS-929075**’s of N-methylmethanamide at the C3 position of its benzofuran core and 4-methyl-N-(pyrimidin-2-ylcyclopropyl)benzamide at the C5 position also undergo a certain degree of rotation upon binding to NS5B^GT2a^ polymerase (Figure 5C), and the magnitude of the rotation of N-methylmethanamide is about the same as that in the NS5B^GT1b^/**BMS-929075** system. Although the rotation of N-methylethanamide prevents it from forming effective hydrogen bonds with Ser365 and weakens the interaction between them (ΔΔG = 1.53 kcal/mol), the carbonyl oxygen of the 4-methyl-N-(pyrimidin-2-ylcyclopropyl) benzamide can form an effective hydrogen bond with Arg200 and Gln^2a^414 after the rotation ([NH2-HH22 (Arg200) ··· O3 (**BMS-929075**)] and [NE2-HE21 (Gln414) ··· O3 (**BMS-929075**)]). In the NS5B^GT2a^/**BMS-929075** system, the binding affinity between this inhibitor and Arg200 is significantly higher compared to the NS5B^GT1a^/**BMS-929075** system. According to the per-residue energy decomposition results (Figure S9A,C), this is mainly due to the enhanced van der Waals interaction between NS5B^GT2a^ and **BMS-929075**. Although the hydrogen bond formed between them greatly enhances their electrostatic interaction, this contribution is completely offset by the contribution of polar solvation (ΔE_ele_ + ΔG_GB_ = 0.65 kcal/mol). Unlike the above situation, after the mutation of Met414 in NS5B^GT1a^ polymerase to Gln414 in NS5B^GT2a^ polymerase, the hydrogen bond formed between this residue and **BMS-929075** enhances the net electrostatic contribution of this residue (ΔE_ele_ + ΔG_GB_ = −0.66 kcal/mol), thereby enhancing the binding strength of the two (Figures S3B and S9A,C). In addition, the rotation of the substituent at the C5 position of the **BMS-929075** benzofuran core decreases the distances between it and the nearby residues Ser196, Pro197, Trp550, and Phe551 (Figure S7B,C,I,J) and thus enhances the strength of its binding to these residues (Figure S3B), as is the case in the NS5B^GT1b^/**BMS-929075** system. However, both the van der Waals and electrostatic interactions between Tyr448 and the inhibitor are weakened, resulting in a decrease in their binding affinity (ΔΔG = 0.76 kcal/mol) (Figure S9A,C). Our simulations showed that **BMS-929075** binds to NS5B^GT2a^ polymerase with the substituent at the C5 position of the benzofuran core facing the cavity where residue Phe551 is located (Figure S8C), which is consistent with its binding to NS5B^GT1b^ polymerase.

For the NS5B^GT2b^/**BMS-929075** system (Figure 5D), compared to the NS5B^GT1a^/**BMS-929075** system, the N-methylethanamide at the C3 position of the benzofuran core does not rotate after binding, and also forms a strong hydrogen bond with its neighboring residue Ser365. Although the substituent of C5 also rotates to some extent, it is also oriented toward the cavity where residue Arg394 is located (Figure S8D). Compared with the NS5B^GT1a^/**BMS-929075** system, the distances between the inhibitor and the surrounding residues Phe193, Arg200, and Cys316 are lower in the NS5B^GT2b^/**BMS-929075** system (Figure S7A,D,E), thereby enhancing the energy contributions of these residues to the binding free energy of the system. In contrast, the distance between the inhibitor and residue Gln^2b^414 is greater in this system (Figure S7G), which leads to a significantly lower binding strength between the inhibitor and polymerase (Figure S3B).

#### 2.3.3. The Binding Modes of **MK-8876** with NS5B Polymerases

**MK-8876** is obtained by replacing the cyclopropyl group at the C5 position and the N-(2-hydroxyethyl)methanesulfonamide at the C6 position of the **HCV-796** benzofuran core with tetracyclic 11-fluoropyrido[2′,3′:5,6][1,3]oxazino[3,4-a]indole and N-methylmethanesulfonamide, respectively. From the key residues identified by binding free energy decomposition (Figure 3C), **MK-8876** can establish important interactions with residues Arg200, Leu314, Cys^1a,2a,2b^/Asn^1b^316, Val321, Ser365, Cys366, Ser368, Leu384, and Tyr448 after binding with the four genotypes of NS5B polymerase. Similar to the binding of inhibitor **HCV-796** to the four genotypes of NS5B polymerase, **MK-8876** can not only establish extensive interactions with residues in the palm II subdomain, but also interact with Ser368 in the palm I subdomain and Cys^1a,2a,2b^/Asn^1b^316 and Leu384 in the palm I/III overlap region.

**Figure 6:**
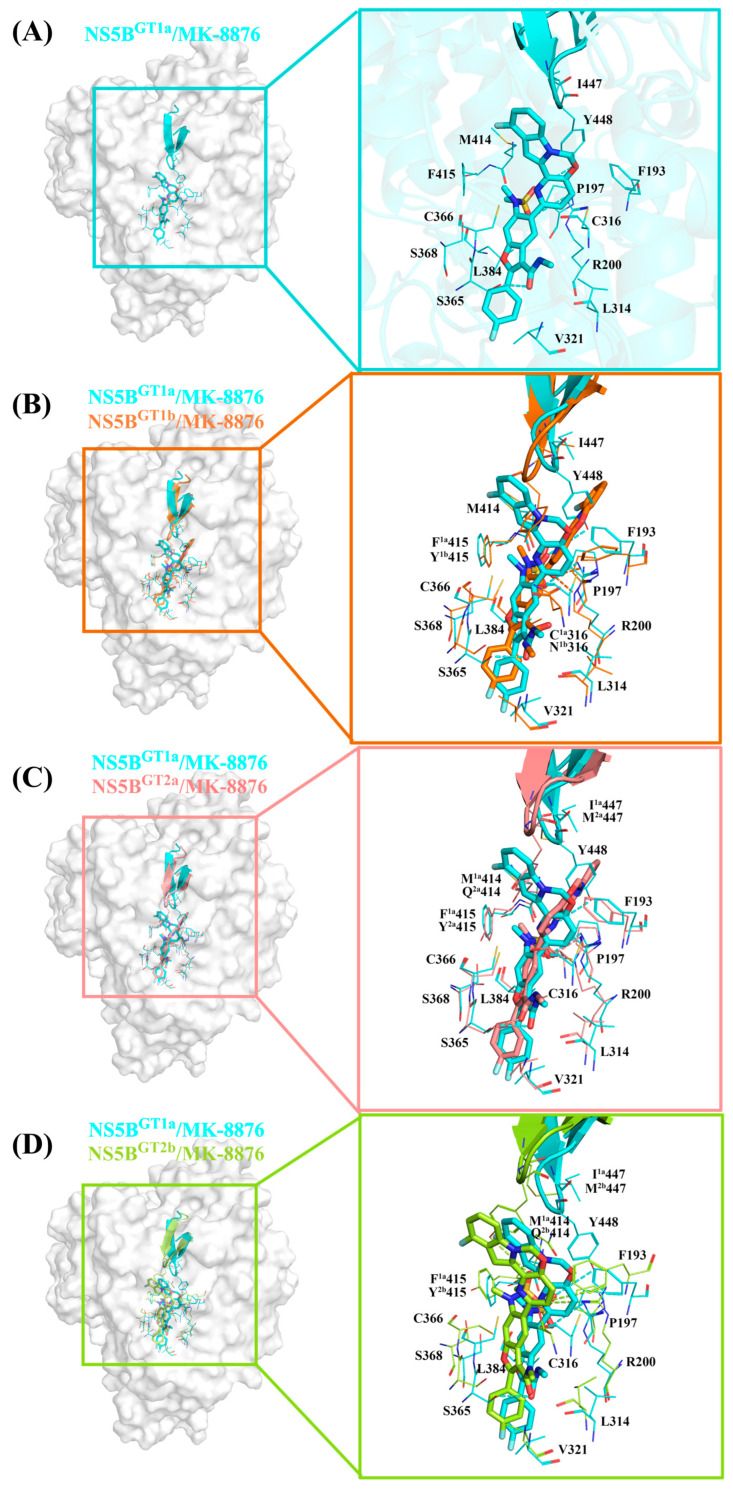
Binding modes of **MK-8876** with four genotypes of NS5B polymerase. The structure shown represents the last frame of the simulation after reaching a steady state. (**A**) Binding modes of NS5B^GT1a^/**MK-8876**. (**B**) Binding modes of NS5B^GT1a^/**MK-8876** and NS5B^GT1b^/**MK-8876**. (**C**) Binding modes of NS5B^GT1a^/**MK-8876** and NS5B^GT2a^/**MK-8876**. (**D**) Binding modes of NS5B^GT1a^/**MK-8876** and NS5B^GT2b^/**MK-8876**.

In the NS5B^GT1a^/**MK-8876** system (Figure 3C and Figure 6A), compared with the NS5B^GT1a^/**HCV-796** system (Figures S5 and S6B), the introduction of a tetracyclic structure at the C5 position of the **MK-8876** benzofuran core markedly enhances its interactions with the nearby residues Phe193 (ΔΔG = −0.79 kcal/mol), Cys316 (ΔΔG = −0.53 kcal/mol), and Tyr448 (ΔΔG = −2.45 kcal/mol). The simulation results indicated that after binding with NS5B^GT1a^ polymerase, the substituent at C5 is oriented toward the cavity where residue Arg394 is located (Figure S10A). Compared to the NS5B^GT1a^/**HCV-796** system, the N-methylmethanesulfonamide at the C6 position of the **MK-8876** benzofuran core undergoes a certain degree of rotation, which enhances its binding strength with the surrounding residues Pro197 (ΔΔG = −0.51 kcal/mol) and Phe415 (ΔΔG = −0.55 kcal/mol). Meanwhile, this group can form a stronger hydrogen bond with Arg200 ([NH1-HH12 (Arg200) ··· O5 (**MK-8876**)]) and a weaker hydrogen bond with Tyr448 ([OH-HH (Tyr448) ··· O4 (**MK-8876**)]). Unlike the hydrogen bond formed in the NS5B^GT1a^/**HCV-796** system, it is the O5 of N-methylmethanesulfonamide at the C6 position of this inhibitor that forms a hydrogen bond with Arg200 in this system. However, the energetic contribution of Arg200 to the binding free energy of this system is lower compared to that of the NS5B^GT1a^/**HCV-796** system (ΔΔG = 1.50 kcal/mol). Moreover, the interaction energy spectra and hydrogen bonding interaction networks of the NS5B^GT1a^/**MK-8876** system and the NS5B^GT1a^/**HCV-796** system are similar (Figure 3A,C).

In the NS5B^GT1b^/**MK-8876** system, the contributions of residues Pro197, Arg200, Ser365, Met414, Ile447, and Tyr448 to the binding free energy are significantly different compared to the contributions of the corresponding residues in the NS5B^GT1a^/**MK-8876** system (Figure S3C). Compared with its binding to NS5B^GT1a^ polymerase, **MK-8876** can form two hydrogen bonds with Arg200 after binding to NS5B^GT1b^ polymerase ([NH2-HH22 (Arg200) ··· O5 (**MK-8876**)] and [NE-HE (Arg200) ··· O5 (**MK-8876**)]), and the distance between the inhibitor and polymerase is reduced (Figure S11B), which enhances the contribution of this residue to the binding free energy of the system (Figure S3C). However, **MK-8876** fails to form hydrogen bonds with residues Ser365 and Tyr448 (Table 2 and Figure 6B), resulting in a low binding strength between the inhibitor and these two residues (ΔΔG_Ser365_ = 0.69 kcal/mol, ΔΔG_Tyr448_ = 2.51 kcal/mol). This is mainly due to the rotation of N-methylmethanamide at the C3 position and the tetracyclic structure at the C5 position of the **MK-8876** benzofuran core. In addition, the tetracyclic structure at the C5 position of the **MK-8876** benzofuran core rotates toward the cavity where residue Phe551 is located (Figure S10B), reducing the distances to the surrounding residues Pro197, Met414, and Ile447 (Figure S11A,E,G) and increasing the binding strength with these residues (Figures S3C and S12A,B).

Compared with binding to NS5B^GT1a^ polymerase, the tetracyclic structure at the C5 position of the **MK-8876** benzofuran core upon binding to NS5B^GT2a^ polymerase undergoes a certain degree of rotation toward the cavity in which residue Phe551 is located (Figure S10C), which is consistent with the situation in the NS5B^GT1b^/**MK-8876** system. The hydrogen bond calculations (Table 2) showed that compared to the situation in the NS5B^GT1a^/**MK-8876** system, **MK-8876** can form two different hydrogen bonds with Arg200 after binding to NS5B^GT2a^ polymerase ([NH1-HH11 (Arg200) ··· O5 (**MK-8876**)] and [NE-HE (Arg200) ··· O5 (**MK-8876**)]), in addition to forming the same hydrogen bond with residue Ser365. Although the inhibitor forms the same hydrogen bond with residue Ser365 in both systems, the electrostatic interaction is stronger in the NS5B^GT2a^/**MK-8876** system, which increases the energy contribution of Ser365 to the binding free energy of the system (Figures S3C and S12A,C). The rotation of the C5 position tetracyclic ring structure of the **MK-8876** benzofuran core prevents it from forming hydrogen bonds with the nearby residue Tyr448, which weakens the bonding strength between them (ΔΔ G = 2.09 kcal/mol). At the same time, this rotation also reduces the distances between the inhibitor to residues Pro197 and Met^2a^447 (Figure S11A,G), resulting in enhanced interactions between the inhibitor and these two residues (Figures S3C and S12A,C).

For the NS5B^GT2b^/**MK-8876** system, the binding conformation of **MK-8876** does not involve significant rotations compared to the NS5B^GT1a^/**MK-8876** system, and the tetracyclic ring structure at the C5 position of the benzofuran core is also oriented toward the cavity where residue Arg394 is located (Figure S10D). After binding with NS5B^GT2b^ polymerase, **MK-8876** can also form hydrogen bonds with Arg200 and Tyr448 (Table 2), but the occupancy rate of the hydrogen bond formed between the N-methylethanamide at the C3 position of its benzofuran core and residue Ser365 is only 4.60%, and the interaction between them is weak (Figure S12A,D). Compared with binding to NS5B^GT1a^ polymerase, **MK-8876** forms two hydrogen bonds with residue Arg200 after binding to NS5B^GT2b^ polymerase, but the distance between the two increases (Figure S11B), resulting in a decrease in their binding strength (ΔΔG = 0.85 kcal/mol). On the contrary, in the NS5B^GT2b^/**MK-8876** system, the hydrogen bond formed between the inhibitor and residue Tyr448 further enhances the electrostatic and van der Waals interactions between them compared to the NS5B^GT1a^/**MK-8876** system (Figure S12A,D), thereby enhancing the energy contribution of Tyr448 to the binding free energy of the system (Figure S3C). The simulation results showed that compared to binding to NS5B^GT1a^ polymerase, **MK-8876** moves toward Leu314 and Cys366 after binding to NS5B^GT2b^ polymerase, reducing the distances to these two residues (Figure S11C,D), and increasing the distances to Gln^2b^414 and Tyr^2b^415 (Figure S11E,F), which is also confirmed by the changes in the contribution of these residues to the binding free energy of the system (Figure S12A,D).

#### 2.3.4. The Binding Modes of Compound **2** with NS5B Polymerases

Compound **2** can be obtained by replacing the cyclopropyl group at the C5 position and the N-(2-hydroxyethyl)methanesulfonamide at the C6 position of **HCV-796** benzofuran core with 3-[(4-fluorophenyl)methyl]-3,4-dihydro-2H-pyrido[2,3-e][1,3]oxazin-4-one and N-methylmethylethanesulfonamide, respectively. The calculations showed that compound **2** can establish important interactions with residues Arg200, Leu314, Cys^1a,2a,2b^/Asn^1b^316, Val321, Ser365, Cys366, Ser368, Leu384, and Tyr448 after binding to the four genotypes of NS5B polymerase (Figure 3D), which is consistent with the binding of **MK-8876** to the four genotypes of NS5B polymerase. Among them, Ser368 is located in the palm I subdomain, Cys^1a,2a,2b^/Asn^1b^316 and Leu384 are located in the palm I/III overlap region, and other residues are all located in the palm II subdomain.

**Figure 7:**
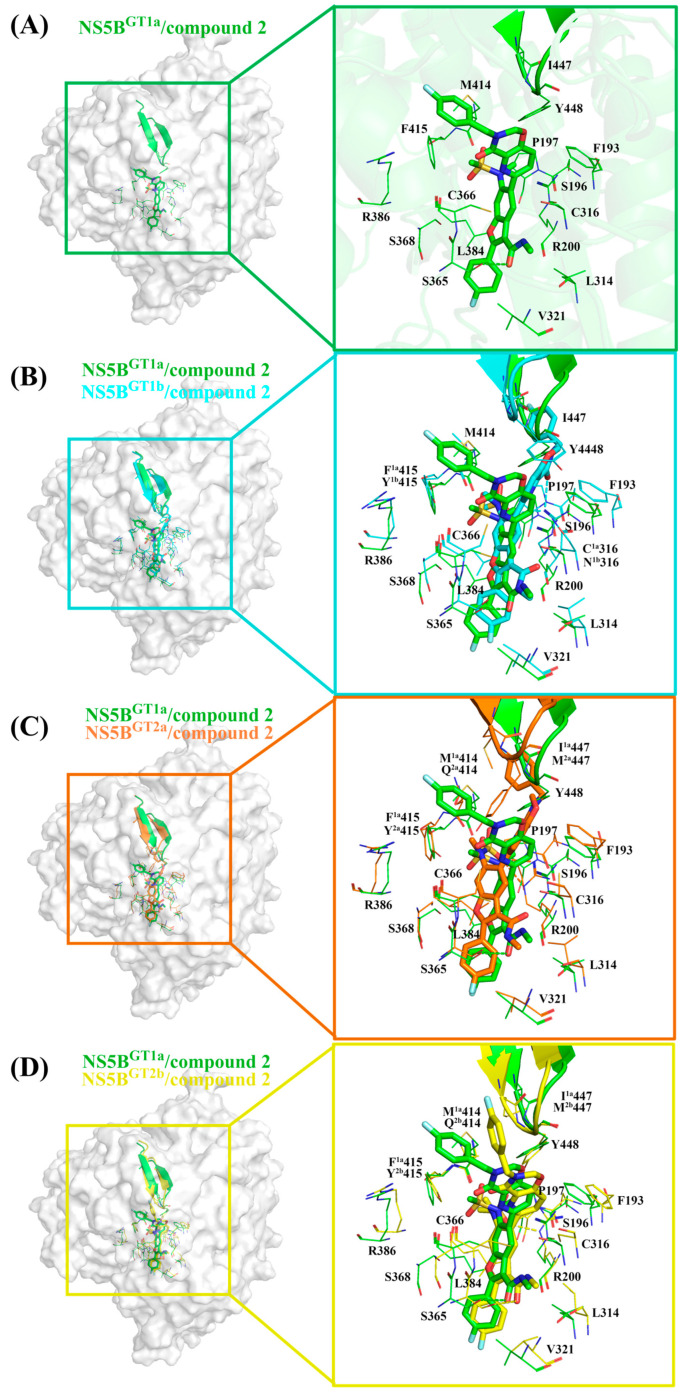
Binding modes of compound **2** with four genotypes of NS5B polymerase. The structure shown represents the last frame of the simulation after reaching a steady state. (**A**) Binding modes of NS5B^GT1a^/compound **2**. (**B**) Binding modes of NS5B^GT1a^/compound **2** and NS5B^GT1b^/compound **2**. (**C**) Binding modes of NS5B^GT1a^/compound **2** and NS5B^GT2a^/compound **2**. (**D**) Binding modes of NS5B^GT1a^/compound **2** and NS5B^GT2b^/compound **2**.

In the NS5B^GT1a^/compound **2** system (Figure 3D and Figure 7A), compared with the NS5B^GT1a^/**HCV-796** system (Figures S5 and S6C), the N-methylethanamide at the C3 position of the benzofuran core of compound **2** can still form a hydrogen bond with Ser365 ([OG-HG (Ser365) ··· O4 (compound **2**)]), but the strength of the hydrogen bond and the binding strength of the residue with the inhibitor are weaker (ΔΔG = 0.75 kcal/mol). At this site, the interaction between the inhibitor and the nearby residue Cys366 is enhanced (ΔΔG = −0.77 kcal/mol). Compared to **HCV-796**, the introduction of 3-[(4-fluorophenyl)methyl]-3,4-dihydro-2H-pyrido[2,3-e][1,3]oxazin-4-one at the C5 position of the benzofuran core enhances its interactions with the nearby residues Phe193 (ΔΔG = −0.80 kcal/mol), Cys316 (ΔΔG = −0.64 kcal/mol), and Gly449 (ΔΔG = −0.61 kcal/mol). Our simulation results showed that after binding with NS5B^GT1a^ polymerase, the substituent at the C5 position of the benzofuran core of compound **2** is oriented toward the cavity where the Arg394 residue is located (Figure S13A). In addition, compared with the situation in the NS5B^GT1a^/**HCV-796** system, the N-methylethanesulfonamide at the C6 position of the inhibitor’s benzofuran core in the NS5B^GT1a^/compound **2** system fails to form effective hydrogen bonds with the nearby residue Arg200, and the binding strength between them is significantly weaker (ΔΔG = 3.56 kcal/mol). Here, the interaction between compound **2** and Phe415 is weaker (ΔΔG = 0.88 kcal/mol), while the interaction with Arg386 is stronger (ΔΔG = −1.09 kcal/mol). Additionally, the interaction energy spectra of the NS5B^GT1a^/compound **2** system and the NS5B^GT1a^/**HCV-796** system are similar (Figure 3A,D).

The energetic difference spectra of the key residues (Figure S3D) showed that the contributions of residues Ser196, Pro197, Arg200, Asn^1b^316, Ser365, Cys366, Arg386, Met414, Tyr^1b^415, and Ile447 to the binding free energy in the NS5B^GT1b^/compound **2** system are significantly different from those in the NS5B^GT1a^/compound **2** system. Compared with the situation in the NS5B^GT1a^/compound **2** system, the N-methylethanamide at the C3 position, the bicyclic structure at the C5 position, and the N-methylethanesulfonamide at the C6 position of the benzofuran core all undergo a certain degree of rotation after binding with NS5B^GT1b^ polymerase (Figure 7B), forming a new hydrogen bonding network. The hydrogen bond calculations (Table 2) indicated that the bicyclic structure at the C5 position and the N-methylmethanesulfonamide at the C6 position of the inhibitor can form strong hydrogen bonds with residue Arg200 ([NH1-HH11 (Arg200) ··· O2 (compound **2**)] and [NH1-HH12 (Arg200) ··· O6 (compound **2**)]). Furthermore, the binding strength between them is notably higher (Figure S3D). However, the rotation of the N-methylethanamide at the C3 position of the compound **2** benzofuran core prevents it from forming hydrogen bonds with residue Ser365, which reduces the contribution of Ser365 to the binding free energy of the system (Figure S3D). At the same time, this rotation also brings the carbonyl oxygen of N-methylethanamide and the carbonyl oxygen of the nearby residue Asn^1b^316 side chain closer (Figure S14C), and the repulsion between the two makes the electrostatic contribution of Asn^1b^316 to the binding free energy of the system positive (0.76 kcal/mol), thus weakening the binding strength between them (Figure S15B). Compared with the situation in the NS5B^GT1a^/compound **2** system, the bicyclic structure at the C5 position in the NS5B^GT1b^/compound **2** system rotates and moves toward the cavity where residue Phe551 is located (Figure S13B). This rotation also results in a shorter distance between the inhibitor and the surrounding residues Ser196, Met414, and Ile447 (Figure S14A,F,H), strengthening the binding force of the inhibitor to these residues. However, the distance to Cys366 increases (Figure S14D), which weakens the binding of the inhibitor to this residue (Figure S3D). In addition, the rotation of N-methylmethanesulfonamide at the C6 position leads to a decrease in the distances to residues Pro197 and Tyr^1b^415 (Figure S14B,G) and an increase in the distance to Arg386 (Figure S14E). This also causes changes in the binding strength between the inhibitor and these residues (Figure S3D).

For the NS5B^GT2a^/compound **2** system, the N-methylethanamide at the C3 position, the bicyclic structure at the C5 position, and the N-methylethanesulfonamide at the C6 position of the inhibitor benzofuran core also undergo a similar rotation as in the NS5B^GT1b^/compound **2** system, with the bicyclic structure at the C5 position oriented toward the cavity where residue Phe551 is located (Figure S13C). Unlike the NS5B^GT1b^/compound **2** system, although compound **2** can also form two effective hydrogen bonds with Arg200 of NS5B^GT2a^ polymerase, both hydrogen bonds are formed by the O6 atom of the inhibitor and the guanidine group of Arg200 ([NE-HE (Arg200) ··· O6 (compound **2**)] and [NH1-HH11 (Arg200) ··· O6 (compound **2**)]). The residues whose contributions to the binding free energy in the NS5B^GT2a^/compound **2** system are quite different from those in the NS5B^GT1a^/compound **2** system and are similar to those in NS5B^GT1b^/compound **2** system. Moreover, the reasons for the energy differences in the contributions of the residues Ser196, Pro197, Arg200, Cys366, Arg386, Gln^2a^414, Tyr^2a^415, and Met^2a^447 are the same as those for the corresponding residues in the NS5B^GT1b^/compound **2** system (Figure S15A,C). Moreover, in the NS5B^GT2a^/compound **2** system, the distance between the inhibitor and residue Cys316 is greater due to the rotation of the C5 bicyclic ring structure compared to the situation in the NS5B^GT1a^/compound **2** system (Figure S14C), thereby resulting in a lower binding strength between the inhibitor and polymerase (Figure S3D).

Similar to the binding to NS5B^GT1a^ polymerase, compound **2** also orients toward the cavity where residue Arg394 is located after binding to NS5B^GT2b^ polymerase, although the bicyclic structure at the C5 position and N-methylethanesulfonamide at the C6 position of the benzofuran core also undergo some degree of rotation (Figure S13D). This inhibitor can form a hydrogen bond with Ser365, similar to that in the NS5B^GT1a^/compound **2** system, as well as a hydrogen bond with Tyr448 ([OH-HH (Tyr448) ··· O5 (compound **2**)]) and two hydrogen bonds with Arg200 ([NE-HE (Arg200) ··· O6 (compound **2**)] and [NH2-HH22 (Arg200) ··· O6 (compound **2**)]). These hydrogen bonds significantly enhance the binding strength of the inhibitor with Tyr448 and Arg200 (Figures S3D and S15A,D). Additionally, the residues in the NS5B^GT2b^/compound **2** system that contribute significantly to the binding free energy that do not contribute significantly in the NS5B^GT1a^/compound **2** system include Pro197, Arg386, Gln^2b^414, and Tyr^2b^415 (Figure S3D). The reason for the differences in the contributions of Pro197, Arg386, and Tyr^2b^415 to the binding free energy of the system is the same as for the corresponding residues in the NS5B^GT1b^/compound **2** system (Figure S14B,E,G). The Met414 in NS5B^GT1a^ polymerase is mutated to Gln414 in NS5B^GT2b^ polymerase; the strongly polar residue Gln414 enhances the electrostatic interaction with N-methylmethanesulfonamide at the C6 position of the inhibitor. However, the absolute value of the change in electrostatic interaction contribution between the two groups before and after mutation is less than that of the polar solvation contribution (ΔE_ele_ + ΔG_GB_ = 1.47 kcal/mol), which leads to a lower energy contribution of the residue to the binding free energy of the system (Figures S3D and S15A,D).

#### 2.3.5. The Binding Modes of Compound **9B** with NS5B Polymerases

The structure of compound **9B** is similar to that of compound **2**, with only some differences in the bicyclic structure at the C5 position of the benzofuran core. Compound **9B** can be obtained by replacing the cyclopropyl group at the C5 position and the N-(2-hydroxyethyl)methanesulfonamide at the C6 position of the **HCV-796** benzofuran core with 3-[(4-fluorophenyl)methyl]-2-methyl-3,4-dihydroquinazolin-4-one and N-methylmethanesulfonamide, respectively. From the key residues identified by binding free energy decomposition (Figure 3E), compound **9B** can establish important interactions between residues Leu314, Cys^1a,2a,2b^/Asn^1b^316, Val321, Ser365, Cys366, Ser368, and Leu384 and the four genotypes of NS5B polymerase. Among them, Ser368 is located in the palm I subdomain, Cys^1a,2a,2b^/Asn^1b^316 and Leu384 are located in the palm I/III overlap region, and the remaining residues are all located in the palm II subdomain. This is similar to the binding modes of inhibitors **HCV-796**, **MK-8876**, and compound **2** to the four genotypes of NS5B polymerase.

**Figure 8:**
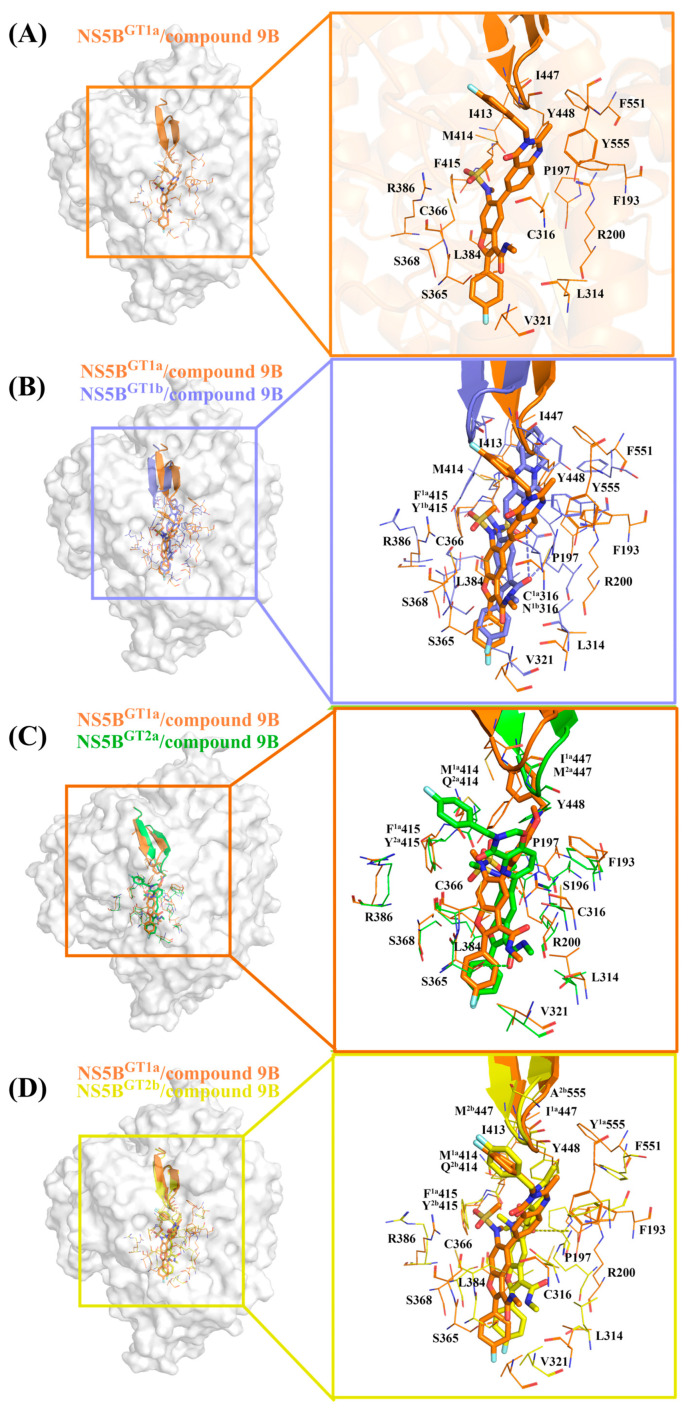
Binding modes of compound **9B** with four genotypes of NS5B polymerase. The structure shown represents the last frame of the simulation after reaching a steady state. (**A**) Binding modes of NS5B^GT1a^/compound **9B**. (**B**) Binding modes of NS5B^GT1a^/compound **9B** and NS5B^GT1b^/compound **9B**. (**C**) Binding modes of NS5B^GT1a^/compound **9B** and NS5B^GT2a^/compound **9B**. (**D**) Binding modes of NS5B^GT1a^/compound **9B** and NS5B^GT2b^/compound **9B**.

In the NS5B^GT1a^/compound **9B** system (Figure 3E and Figure 8A), the introduction of 3- [(4-fluorophenyl) methyl]-2-methyl-3,4-dihydroquinazolin-4-one enhances its interactions with nearby residues Cys316 (ΔΔG = −0.71 kcal/mol), Cys366 (ΔΔG = −0.65 kcal/mol), Gly449 (ΔΔG = −0.82 kcal/mol), and Tyr555 (ΔΔG = −0.79 kcal/mol) compared with the NS5B^GT1a^/**HCV-796** system (Figures S5 and S6D). Like the other inhibitors with longer substituents at the C5 position of the benzofuran core, the simulation results indicated that the substituent at the C5 position of compound **9B** after binding with NS5B^GT1a^ polymerase is also oriented toward the cavity where residue Arg394 is located (Figure S16A). Moreover, the N-methylethanesulfonamide at the C6 position of the compound **9B** benzofuran core fails to form effective hydrogen bonds with the nearby residue Arg200, resulting in a significantly lower binding strength between the two in the NS5B^GT1a^/compound **9B** system (ΔΔG = 4.52 kcal/mol). However, the interaction between this inhibitor and Arg386 near here is stronger (ΔΔG = −1.49 kcal/mol). Additionally, the interaction energy spectra and hydrogen bonding networks of the NS5B^GT1a^/compound **9B** system and the NS5B^GT1a^/**HCV-796** system are similar (Figure 3A,E).

From the energetic difference spectra of the key residues (Figure S3E), it can be seen that the energy contributions of residues Phe193, Pro197, Arg200, Ser365, Cys366, Arg386, Ile413, Met414, Ile447, Tyr448, Phe551, and Tyr555 to the binding free energy in the NS5B^GT1b^/compound **9B** system are significantly different from those in the NS5B^GT1a^/compound **9B** system. Compared with the situation in the NS5B^GT1a^/compound **9B** system, the substituents at the C3, C5, and C6 positions of the benzofuran core of compound **9B** all undergo a certain degree of rotation (Figure 8B) after binding to NS5B^GT1b^ polymerase, thus forming a new hydrogen bonding network. The hydrogen bond calculations showed (Table 2) that after the rotation of N-methylmethanamide at the C3 position of the inhibitor in the NS5B^GT1b^/compound **9B** system, its carbonyl oxygen can form a weak hydrogen bond with residues Arg200 and Asn^1b^316 ([NH1-HH12 (Arg200) ··· O3(compound **9B**)] and [ND2-HD21(Asn316) ··· O3(compound **9B**)]). Moreover, the distance between the inhibitor and Arg200 is closer (Figure S17C), and the binding force between the two is stronger (Figure S3E). Meanwhile, this rotation prevents the formation of hydrogen bonds between the inhibitor and Ser365, resulting in a decrease in the binding strength between them (Figures S3E and S18A,B). Compared with the NS5B^GT1a^/compound **9B** system, the bicyclic structure at the C5 position of compound **9B** is oriented toward the cavity where Phe551 is located after binding to NS5B^GT1b^ polymerase (Figure S16B), and the distances between compound **9B** and the surrounding residues Ile413, Met414, Ile447, Phe551, and Tyr555 are reduced (Figure S17G,H,J,L,M), thereby enhancing the energy contributions of these residues to the binding free energy of the system (Figure 3E). Conversely, this rotation increases the distances between the inhibitor and residues Phe193, Cys366, and Tyr448 (Figure S17A,C,K), thus weakening the binding strength between the inhibitor and these residues (Figure S3E). In addition, the rotation of N-methylethanesulfonamide at the C6 position of the inhibitor benzofuran core reduces the distance to Pro197 (Figure S17B), while increasing the distance to Arg386 (Figure S17F), which also causes corresponding changes in the binding strength between the inhibitor and these residues (Figure S3E).

After binding with NS5B^GT2a^ polymerase, the N-methylethanamide at the C3 position, the bicyclic structure at the C5 position, and the N-methylethanesulfonamide at the C6 position of the compound **9B** benzofuran core undergo similar rotations as in the NS5B^GT1b^/compound **9B** system (Figure 8C), and the bicyclic structure at the C5 position also orients toward the cavity where Phe551 is located (Figure S16C). Compared with binding to NS5B^GT1a^ polymerase, compound **9B** can form two hydrogen bonds with residue Arg200 after binding to NS5B^GT2a^ polymerase ([NH1-HH11 (Arg200) ··· O5 (compound **9B**)] and [NH1-HH12 (Arg200) ··· O1 (compound **9B**)]), and the binding strength between the two is significantly stronger (Figure S3E). However, the binding strength between this inhibitor and Ser365 is weaker (Figure S3E) for the same reason as in the NS5B^GT1b^/compound **9B** system. In this system, the rotation of the bicyclic structure at the C5 position reduces the distances between compound **9B** and the residues Phe193, Ile413, Tyr448, and Phe551 (Figure S17A,G,K,L), thus increasing the energy contributions of these residues to the binding free energy of the system. The distances to the residues Cys316, Cys366, and Ala^2a^555 (Figure S17D,E,M) also increases, thereby reducing the energy contribution of these residues (Figure S3E). In addition, the rotation of N-methylethanesulfonamide at the C6 position of the compound **9B** benzofuran core reduces its distances to the residues Pro197 and Tyr^2a^415 (Figure S17B,I), while increasing its distance to Arg386 (Figure S17F), which also causes corresponding changes in the binding strength between these residues and the inhibitor (Figure S3E).

Compared to the situation in the NS5B^GT1a^/compound **9B** system, the N-methylethanamide at the C3 position and N-methylethanesulfonamide at the C6 position of the benzofuran core of compound **9B** undergo significant rotations after binding with NS5B^GT2b^ polymerase. However, the bicyclic structure at the C5 position exhibits less rotation and is similarly oriented toward the cavity where residue Arg394 is located (Figure S16D). The MD simulations indicated that the inhibitor can form a strong hydrogen bond with Tyr448 ([OH-HH (Tyr448) ··· O4 (compound **9B**)) and a weak hydrogen bond with Arg200 ([NH2-HH22 (Arg200) ··· O5 (compound **9B**)]). These hydrogen bonds enhance the binding strength between the inhibitor and residues Arg200 and Tyr448 (Figure S3E). In the NS5B^GT2b^/compound **9B** system, the rotation of the N-methylethanamide at the C3 position of the inhibitor benzofuran core prevents it from forming hydrogen bonds with Ser365, resulting in a corresponding decrease in the binding strength between them (Figure S3E). However, this rotation enhances its interaction with nearby residue Leu314 (Figures S3E and S18A,D). Compared with binding to NS5B^GT1a^ polymerase, the binding strength between the inhibitor and residues Pro197 and Tyr^2b^415 of NS5B^GT2b^ polymerase is stronger, while the binding strength with Arg386 is weaker (Figure S3E). The reason for this is the same as the reason for the corresponding residues in the NS5B^GT2a^/compound **9B** system (Figure S18B,F,I). The mutation from Met414 in NS5B^GT1a^ polymerase to Gln414 in NS5B^GT2b^ polymerase causes the interaction between the inhibitor and the residue at this site to be weaker (Figure S3E), and the reason for this is the same as the one for the corresponding residue in the NS5B^GT2b^/compound **2** system. Additionally, although there is no obvious rotation of the C5 bicyclic structure of the compound **9B** benzofuran core, the distance between the inhibitor and the nearby residue Phe193 is shorter, and the binding strength between them is stronger (Figure S18A). Compared with the NS5B^GT1a^/compound **9B** system, the contribution of Ala^2b^555 to the binding free energy of the NS5B^GT2b^/compound **9B** system is weaker due to the dual effects of the mutation and distance change between Ala^2b^555 and the inhibitor (Figures S3E and S18M).

From the analyses of the above simulation results, it can be summarized that the five benzofuran core inhibitors (**HCV-796**, **BMS-929075**, **MK-8876**, compound **2**, and compound **9B**) can not only establish extensive interactions with residues in the palm II subdomain, but also interact with residues in the palm I subdomain or palm I/III overlap region after binding with the four genotypes of NS5B polymerase (GT**1a**, **1b**, **2a**, and **2b**). And, most of the key residues around the benzofuran core of this series of inhibitors are polar residues. Interestingly, the benzofuran inhibitors with longer substituents at the C5 position (**BMS-929075**, **MK-8876**, compound **2**, and compound **9B**) are oriented toward the cavity where Arg394 is located when binding with GT1a and 2b NS5B polymerases, and toward the cavity where Phe551 is located for GT1b and 2a NS5B polymerases. In addition, after binding with GT1a NS5B polymerase, the N-methylethanamide at the C3 position of these five benzofuran inhibitors is able to form hydrogen bonds with residue Ser365. However, when binding with GT1b NS5B polymerase, this group almost rotates 180° and cannot form hydrogen bonds with Ser365. There is no obvious pattern in the orientation of this group after binding with GT2a and 2b NS5B polymerases. Furthermore, in order to investigate the reasons for the characteristic binding patterns exhibited by this series of inhibitors when binding to the four genotypes of NS5B polymerase, we conducted ASMD simulation studies.

### 2.4. Identification of the Binding Pathway of Benzofuran Inhibitors to NS5B Polymerases

Before performing the ASMD simulations, it was necessary to determine the pathway through which the inhibitor binds to the corresponding protein. Here, the ASMD simulation of the dissociation process between the inhibitor and protein was conducted to explore the details, starting from the final MD simulation stable structure of each system. This study used the complex systems of **MK-8876** and the four genotypes of NS5B polymerase as representatives to conduct binding channel analysis calculations using CAVER 3.0 software. This software can evaluate the channel performance through the bottleneck radius, length, and curvature. The starting point for the calculations was the position of the ligand in the steady-state structure, with the minimum probe radius, clustering threshold, shell depth, and shell radius set to 1.2, 3.5, 4.0, and 3.0 Å, respectively.

**Figure 9:**
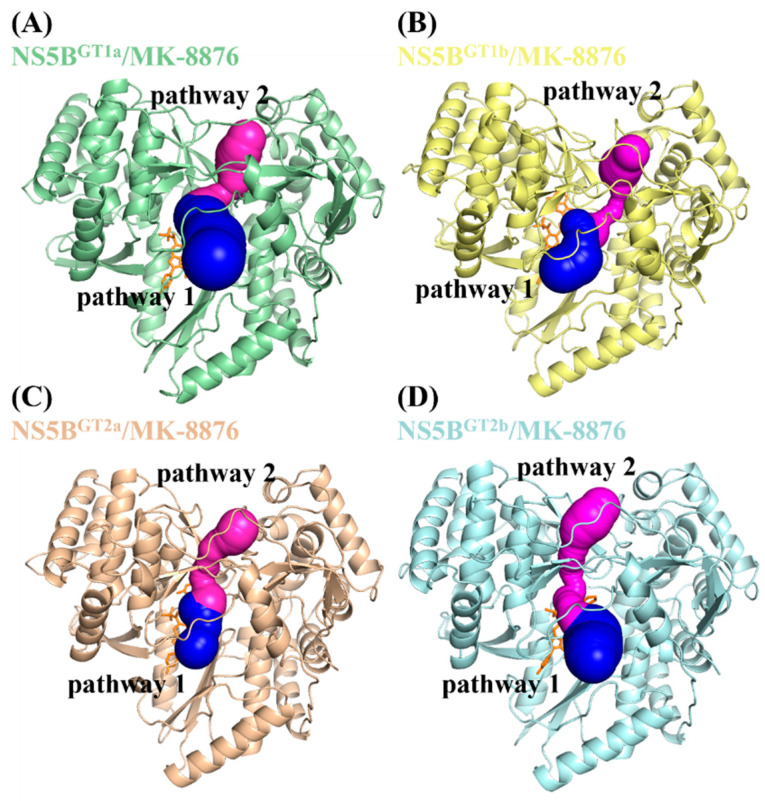
Two top ranked pathways identified by CAVER 3.0 for (**A**) NS5B^GT1a^/**MK-8876**, (**B**) NS5B^GT1b^/**MK-8876**, (**C**) NS5B^GT2a^/**MK-8876**, and (**D**) NS5B^GT2b^/**MK-8876** complexes.

The two calculated dominant channels that **MK-8876** uses to bind to the four genotypes of NS5B polymerase are shown in Figure 9, named pathway 1 (blue) and pathway 2 (purple), respectively. From the channel characteristic data listed in Table 3, it can be seen that among the four genotypes of NS5B polymerase, the bottleneck radius of pathway 1 is greater than that of pathway 2, while the length and curvature are both smaller than that of pathway 2. Therefore, pathway 1 is the more advantageous pathway among the four genotypes of NS5B polymerase. In the ASMD simulations, pathway 1 was selected as the dissociation channel of the inhibitors.

### 2.5. Analyses of the Binding Processes of Benzofuran Inhibitors to NS5B Polymerases

**Figure 10:**
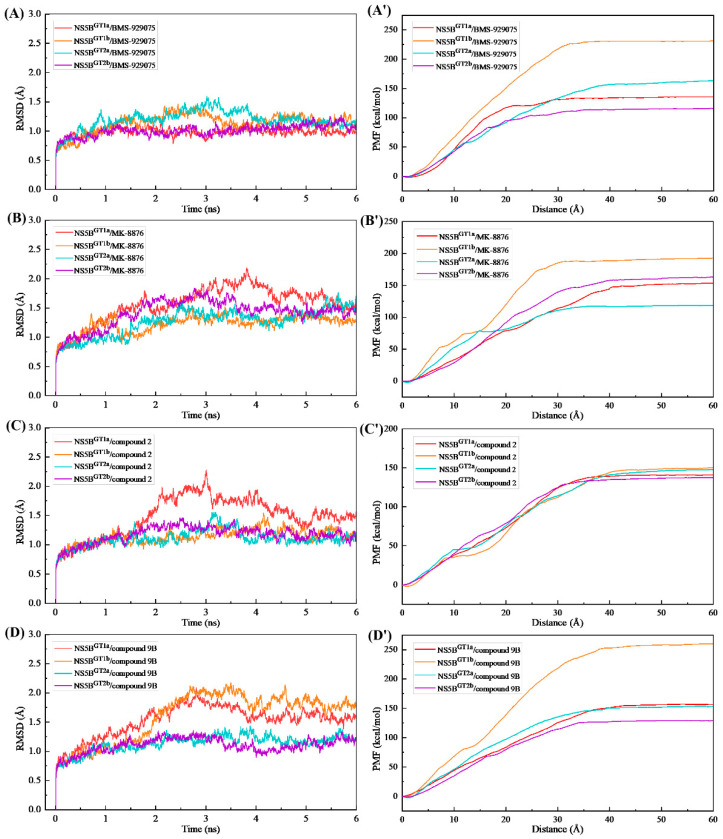
The RSMD of the receptor backbone atoms (CA, C, and O) in (**A**) NS5B/**BMS-929075**, (**B**) NS5B/**MK-8876**, (**C**) NS5B/compound **2**, and (**D**) NS5B/compound **9B** systems throughout the Jarzynski averaged trajectories, and the PMF profiles along the reaction coordinate for (**A’**) NS5B/**BMS-929075**, (**B’**) NS5B/**MK-8876**, (**C’**) NS5B/compound **2**, and (**D’**) NS5B/compound **9B** systems.

The RMSD changes of the NS5B polymerase backbone atoms during the ASMD simulations of the studied systems (Figure 10) showed that the receptor structures are relatively stable during the binding of the four inhibitors to the four genotypes of NS5B polymerase, with no significant conformational changes or the generation of irrational structures. This indicates that the direction of the external force applied to the inhibitors in these 16 complex systems is reasonable and the ASMD simulations and PMF calculations performed are reliable.

From the calculated PMF curves (Figure 10), it can be seen that the PMF profiles of these complex systems rise slowly from 0 Å (the equilibrium distance between the inhibitor and NS5B polymerase in each system was taken as the zero point of the distance). The distances between the inhibitors and the NS5B polymerases extend to approximately 35 Å as the interactions commence, and then the PMF curves reach equilibrium. During this period, there are no obvious potential barriers, indicating that the binding process of these inhibitors to the NS5B polymerases are relatively smooth. In order to understand the details of the dynamic process of the ligand–receptor binding and the channel residues that play a key role in each complex system, we performed binding free energy decomposition for the complex systems with stretching distances of 0, 5, 10, 15, 20, 25, 30, 35, 40, 45, 50, 55, and 60 Å.

**Figure 11:**
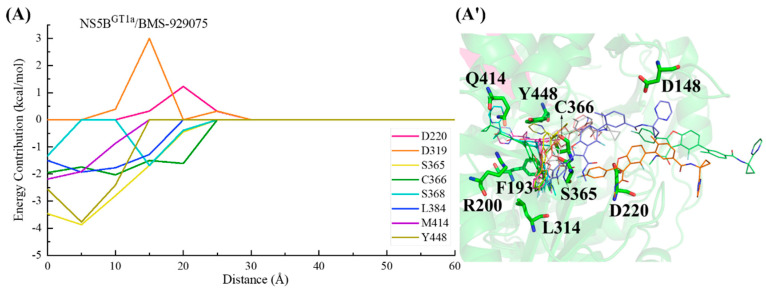
(**A**) Binding energy contributions of residues (>1.00 or <−1.50 kcal/mol) in the dissociation processes for the NS5B^GT1a^/**BMS-929075** system (the distance of **BMS-929075** from the equilibrium of the system is 0, 5, 10, 15, 20, 25, 30, 35, 40, 45, 50, 55 and 60 Å), and (**A’**) the binding path for **BMS-929075** (the color of the structure at 0, 5, 10, 15, 20, 25, 30, and 35 Å is green, cyan, light magenta, yellow, salmon, gray, slate, and orange, respectively).

In the NS5B^GT1a^/**BMS-929075** system, the key residues in the channel were screened based on the criterion of an energy contribution value >1.00 or <−1.50 kcal/mol. In the binding process between **BMS-929075** and NS5B^GT1a^ polymerase (Figure 11), the para-fluorophenyl at the C2 position of the benzofuran core first approaches the binding pocket, which has a guiding effect on the initial binding. Moving from a distance of ~25 to ~15 Å, **BMS-929075** successively establishes interactions with residues Cys366, Ser365, Ser368, and Leu384, prompting the 4-fluoro-2-(4-fluorophenyl)-N-methyl-1-benzofuran-3-carboxamide structure of the inhibitor to bind deeply into the pocket. At a distance from ~15 to ~5 Å, the interactions between the substituent at the C5 position of **BMS-929075** and the residues Met414 and Tyr448 gradually increase, prompting it to continuously approach the reverse β-fold. Although residues Asp220 and Asp319 may sometimes hinder the binding due to their unfavorable polar desolvation during the above process, under the combined action of residues Met414 and Tyr448, **BMS-929075** binds to NS5B^GT1a^ polymerase and its C5 substituent, ultimately moving toward the cavity where Arg394 is located.

**Figure 12:**
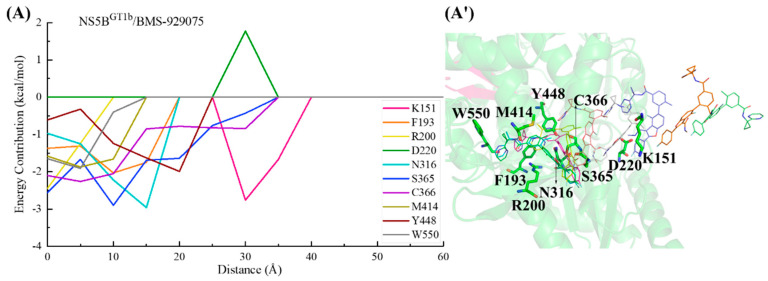
(**A**) Binding energy contributions of residues (>1.00 or <−1.50 kcal/mol) in the dissociation processes for the NS5B^GT1b^/**BMS-929075** system (the distance of **BMS-929075** from the equilibrium of the system is 0, 5, 10, 15, 20, 25, 30, 35, 40, 45, 50, 55, and 60 Å), and (**A’**) the binding path for **BMS-929075** (the color of the structure at 0, 5, 10, 15, 20, 25, 30, 35, and 40 Å is green, cyan, light magenta, yellow, salmon, gray, slate, orange, and lime, respectively).

In the NS5B^GT1b^/**BMS-929075** system, the key residues in the channel were also screened based on the criterion of an energy contribution value >1.00 or <−1.50 kcal/mol. During the binding process between **BMS-929075** and NS5B^GT1b^ polymerase (Figure 12), the para-fluorophenyl at the C2 position of the **BMS-929075** benzofuran core first approaches residue Lys151 when entering the binding pocket, and the interaction between them gradually increases between a distance of ~40 to ~30 Å. The interaction formed between **BMS-929075** and residues Ser365 and Cys366 when the distance is ~35 to ~25 Å facilitates its further binding to NS5B^GT1b^ polymerase. During this period, the interaction between the N-methylmethanamide at the C3 position of **BMS-929075** and Asp220 is unfavorable for binding. Moving from distances of ~25 to ~15 Å, **BMS-929075** successively establishes interactions with residues Tyr448, Asn316, and Phe193. These residues are mainly distributed near the C5 substituent of the inhibitor, and the interactions formed promote the inhibitor to penetrate deeper into the binding pocket. Subsequently (from ~15 to ~5 Å), under the combined action of residues Phe193, Met414, and Trp550, the substituent at the C5 position of **BMS-929075** crosses the reverse β-fold, binding to NS5B^GT1b^ polymerase and becoming oriented toward the cavity where residue Phe551 is located.

**Figure 13:**
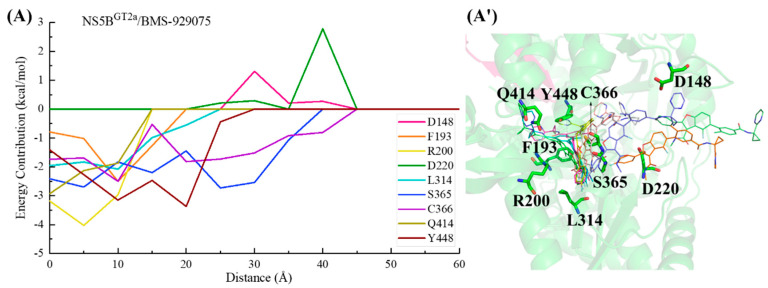
(**A**) Binding energy contributions of residues (>1.00 or <−2.00 kcal/mol) in the dissociation processes for the NS5B^GT2a^/**BMS-929075** system (the distance of **BMS-929075** from the equilibrium of the system is 0, 5, 10, 15, 20, 25, 30, 35, 40, 45, 50, 55, and 60 Å), and (**A’**) the binding path for **BMS-929075** (the color of the structure at 0, 5, 10, 15, 20, 25, 30, 35, and 40 Å is green, cyan, light magenta, yellow, salmon, gray, slate, orange, and lime, respectively).

For the NS5B^GT2a^/**BMS-929075** system, if the key residues are screened according to the criterion of an energy contribution of >1.00 or <−1.50 kcal/mol, the key residues are not prominent enough due to too many residues in the results, so the criterion of a residue energy contribution of >1.00 or <−2.00 kcal/mol was used in this system. Similarly, this screening standard was also adopted in subsequent systems. In the binding process between **BMS-929075** and NS5B^GT2a^ polymerase (Figure 13), the para-fluorophenyl at the C2 position of the **BMS-929075** benzofuran core successively establishes key interactions with residues Cys366 and Ser365 between distances of ~45 and ~30 Å, driving the inhibitor into the binding pocket. During this period, the residues Asp220 and Asp148 located at the entrance of the binding pocket exert varying degrees of hindrance on the binding of this inhibitor to NS5B polymerase. From distances of ~30 to ~20 Å, the substituent at the C5 position of **BMS-929075** gradually approaches Tyr448. The progressively strengthening interactions between them drive the substituent closer to the reverse β-fold. Subsequently (from ~20 to ~5 Å), this substituent interacts with the residues Phe193, Arg200, and Gln414, guiding the C5 substituent toward the cavity where residue Phe551 is located and completing the binding process.

**Figure 14:**
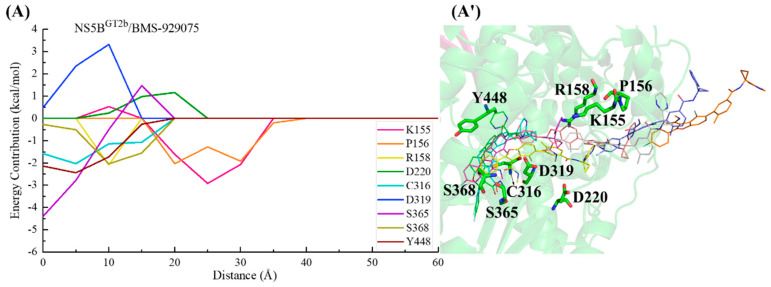
(**A**) Binding energy contributions of residues (>1.00 or <−2.00 kcal/mol) in the dissociation processes for the NS5B^GT2b^/**BMS-929075** system (the distance of **BMS-929075** from the equilibrium of the system is 0, 5, 10, 15, 20, 25, 30, 35, 40, 45, 50, 55, and 60 Å), and (**A’**) the binding path for **BMS-929075** (the color of the structure at 0, 5, 10, 15, 20, 25, 30, 35 Å is green, cyan, light magenta, yellow, salmon, gray, slate and orange, respectively).

In the NS5B^GT2b^/**BMS-929075** system (Figure 14), the para-fluorophenyl group at the C2 position of **BMS-929075** initially interacts with residues Lys155 and Pro156, guiding the inhibitor into the binding pocket. Moving from a distance of ~25 to ~20 Å, the binding of this inhibitor to the NS5B^GT2b^ polymerase is mainly promoted by residues Lys155 and Pro156, and is hindered by Asp220. Subsequently (from ~20 to ~5 Å), the 4-fluoro-2-(4-fluorophenyl)-N-methyl-1-benzofuran-3-carboxamide moiety of **BMS-929075** gradually approaches residues Cys316, Ser365, and Ser368, establishing crucial interactions with them. During this period, the interaction between the substituent at the C5 position of the inhibitor and Tyr448 becomes progressively stronger, leading it closer to the reverse β-fold. Moving from a distance of ~15 to ~5 Å, the interaction between the C5 substituent of **BMS-929075** and Arg158 poses a certain level of hindrance to **BMS-929075**’s approach to the reverse β-fold. Under the combined action of Arg158 and Tyr448, the C5 substituent of **BMS-929075** eventually completes its binding to the NS5B^GT2b^ polymerase, orienting toward the cavity where residue Arg394 is located. During this process, residue Asp319 exerts some hindrance due to its unfavorable polar solvation effect.

**Figure 15:**
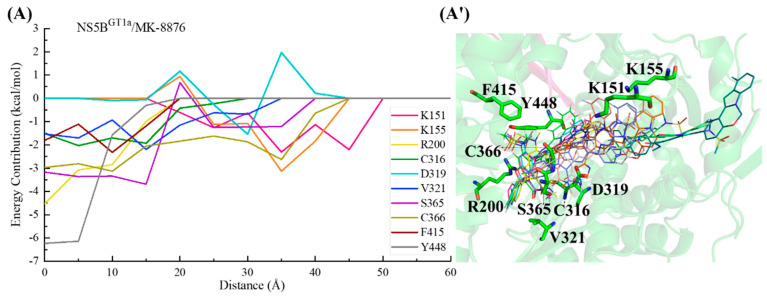
(**A**) Binding energy contributions of residues (>1.00 or <−2.00 kcal/mol) in the dissociation processes for the NS5B^GT1a^/**MK-8876** system (the distance of **MK-8876** from the equilibrium of the system is 0, 5, 10, 15, 20, 25, 30, 35, 40, 45, 50, 55, and 60 Å), and (**A’**) the binding path for **MK-8876** (the color of the structure at 0, 5, 10, 15, 20, 25, 30, 35, 40, and 45 Å is green, cyan, light magenta, yellow, salmon, gray, slate, orange, lime, and dark teal, respectively).

For the NS5B^GT1a^/**MK-8876** system (Figure 15), the C2 position para-fluorophenyl group of **MK-8876** sequentially interacts with residues Lys151, Lys155, Cys366, and Ser365, driving the binding of this inhibitor to NS5B^GT1a^ polymerase. Moving from a distance of ~35 to ~20 Å, **MK-8876** successively interacts with residues Val321 and Cys316. During this period, Asp319 temporarily exerts a repulsive effect on the binding due to unfavorable polar solvation. Subsequently (from ~20 to ~15 Å), the interaction between the N-methylmethanamide at the C3 position of **MK-8876** and residue Ser365 gradually strengthens. With the collaborative assistance of surrounding residues, the spatial position of the inhibitor undergoes significant changes. Its benzofuran moiety becomes deeply embedded in the palm II subdomain of the NS5B^GT1a^ polymerase. Moving from a distance of ~15 to ~5 Å, the progressively stronger interaction between **MK-8876** and Tyr448 causes the tetracyclic structure at the C5 position to approach the reverse β-fold. After binding to NS5B^GT1a^ polymerase, it ultimately orients toward the cavity where residue Arg394 is located.

**Figure 16:**
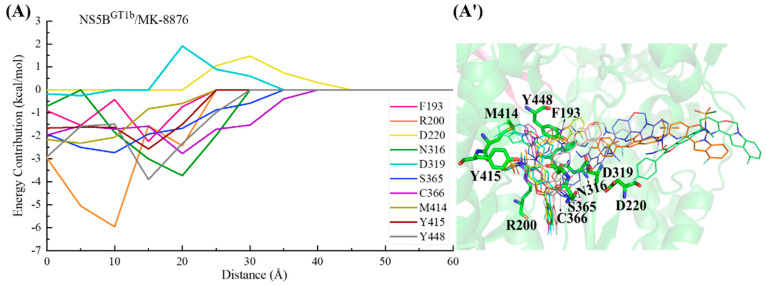
(**A**) Binding energy contributions of residues (>1.00 or <−2.00 kcal/mol) in the dissociation processes for the NS5B^GT1b^/**MK-8876** system (the distance of **MK-8876** from the equilibrium of the system is 0, 5, 10, 15, 20, 25, 30, 35, 40, 45, 50, 55, and 60 Å), and (**A’**) the binding path for **MK-8876** (the color of the structure at 0, 5, 10, 15, 20, 25, 30, 35, and 40 Å is green, cyan, light magenta, yellow, salmon, gray, slate, orange, and lime, respectively).

In the binding process between **MK-8876** and NS5B^GT1b^ polymerase (Figure 16), the para-fluorophenyl group at the C2 position of the inhibitor first establishes a crucial interaction with residue Cys366, guiding the inhibitor into the binding pocket. Moving from a distance of ~35 to ~20 Å, **MK-8876** sequentially establishes significant interactions with the residues Ser365, Asn316, Tyr448, Arg200, Tyr415, Phe193, and Met414, driving the inhibitor deeper into the binding pocket. Throughout this process, residues Asp220 and Asp319 exert some hindrance on the binding of **MK-8876** to NS5B^GT1b^ polymerase. Subsequently (from ~15 to ~5 Å), with the combined action of residues Phe193, Arg200, and Met414, the tetracyclic structure at the C5 position of **MK-8876** crosses the reverse β-fold, leading to its binding to NS5B^GT1b^ polymerase and ultimately orienting toward the cavity where residue Phe551 is located.

**Figure 17:**
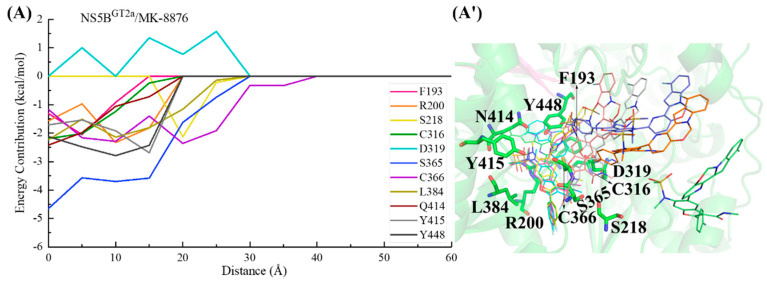
(**A**) Binding energy contributions of residues (>1.00 or <−2.00 kcal/mol) in the dissociation processes for the NS5B^GT2a^/**MK-8876** system (the distance of **MK-8876** from the equilibrium of the system is 0, 5, 10, 15, 20, 25, 30, 35, 40, 45, 50, 55, and 60 Å), and (**A’**) the binding path for **MK-8876** (the color of the structure at 0, 5, 10, 15, 20, 25, 30, 35, and 40 Å is green, cyan, light magenta, yellow, salmon, gray, slate, orange, and lime, respectively).

In the NS5B^GT2a^/**MK-8876** system (Figure 17), during the binding process, the para-fluorophenyl at the C2 position of **MK-8876** first interacts with residue Cys366, guiding the inhibitor into the binding pocket. Subsequently (from ~30 to ~20 Å), the 2-(4-fluorophenyl)-N-methyl-1-benzofuran-3-carboxamide moiety of **MK-8876** interacts with residues Ser218, Ser365, and Leu384. Moving from a distance from ~20 to ~5 Å, the tetracyclic structure at the C5 position establishes crucial interactions with residues Phe193, Arg200, Cys316, Gln414, and Tyr448, further pushing it toward the reverse β-fold. Following the completion of binding with the polymerase, the substituent at the C5 position of the inhibitor is finally oriented toward the cavity where residue Phe551 is located. Throughout the entire binding process, Asp319 exhibits varying degrees of repulsion due to unfavorable polar solvation effects on the binding of **MK-8876** with NS5B^GT2a^ polymerase.

**Figure 18:**
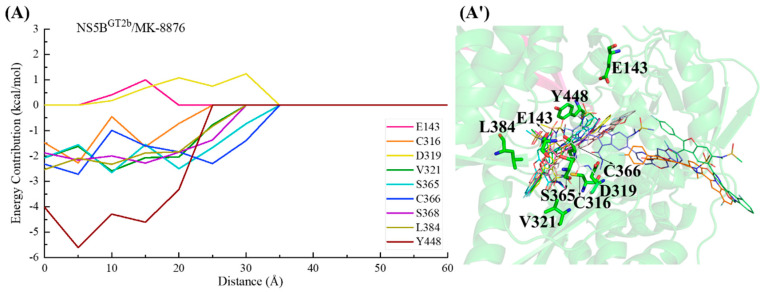
(**A**) Binding energy contributions of residues (>1.00 or <−2.00 kcal/mol) in the dissociation processes for the NS5B^GT2b^/**MK-8876** system (the distance of **MK-8876** from the equilibrium of the system is 0, 5, 10, 15, 20, 25, 30, 35, 40, 45, 50, 55, and 60 Å), and (**A’**) the binding path for **MK-8876** (the color of the structure at 0, 5, 10, 15, 20, 25, 30, 35, and 40 Å is green, cyan, light magenta, yellow, salmon, gray, slate, orange, and lime, respectively).

For the NS5B^GT2b^/**MK-8876** system (Figure 18), the C2-position para-fluorophenyl of **MK-8876** first establishes interactions with residues Cys366 and Ser365, guiding the inhibitor to bind to NS5B^GT2b^ polymerase. Moving from a distance of ~30 to ~20 Å, **MK-8876** sequentially interacts with residues Ser368, Val321, Leu384, Cys316, and Tyr448, causing the benzofuran core of **MK-8876** to penetrate deeply into the binding pocket. During this period, residue Asp319 exerts a certain degree of hindrance on the binding of **MK-8876** to NS5B^GT2b^ polymerase. Moving from a distance from ~20 to ~5 Å, the residue Tyr448 makes a particularly significant contribution to the binding, inducing the substituent at the C5 position of the inhibitor to approach the reverse β-fold, which eventually orients toward the cavity where residue Arg394 is located.

**Figure 19:**
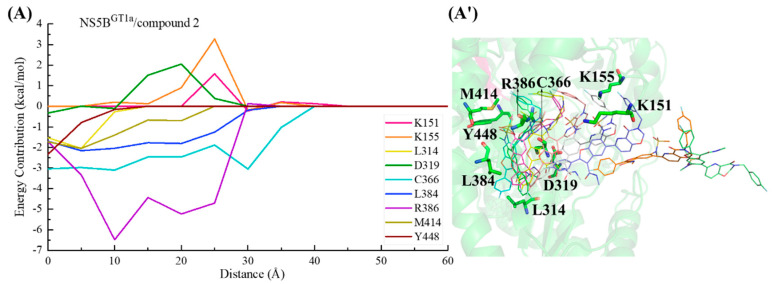
(**A**) Binding energy contributions of residues (>1.00 or <−2.00 kcal/mol) in the dissociation processes for the NS5B^GT1a^/compound **2** system (the distance of compound **2** from the equilibrium of the system is 0, 5, 10, 15, 20, 25, 30, 35, 40, 45, 50, 55, and 60 Å), and (**A’**) the binding path for compound **2** (the color of the structure at 0, 5, 10, 15, 20, 25, 30, 35, and 40Å is green, cyan, light magenta, yellow, salmon, gray, slate, orange, and lime, respectively).

During the binding process of compound **2** with NS5B^GT1a^ polymerase (Figure 19), the para-fluorophenyl at the C2 position of the benzofuran core first interacts with Cys366, guiding the inhibitor into the binding pocket. Moving from a distance of ~30 to ~20 Å, the binding strength between this inhibitor and the residues Arg386, Leu384, and Met414 gradually increases. During this period, residues Lys151, Lys155, and Asp319 exhibit varying degrees of hindrance on the binding due to their unfavorable polar solvation effects. Moving from a distance from ~20 to ~10 Å, residue Arg386 near the N-methylmethanesulfonamide at the C6 position of the benzofuran core makes a prominent contribution to the binding of compound **2** to NS5B^GT1a^ polymerase. Subsequently, induced by residues Met414 and Tyr448, the substituent at the C5 position of the inhibitor approaches the reverse β-fold and eventually orients toward the cavity where residue Arg394 is located.

**Figure 20:**
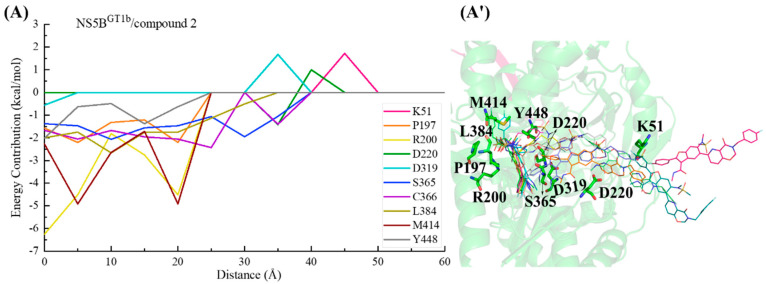
(**A**) Binding energy contributions of residues (>1.00 or <−2.00 kcal/mol) in the dissociation processes for the NS5B^GT1b^/compound **2** system (the distance of compound **2** from the equilibrium of the system is 0, 5, 10, 15, 20, 25, 30, 35, 40, 45, 50, 55, and 60 Å), and (**A’**) the binding path for compound **2** (the color of the structure at 0, 5, 10, 15, 20, 25, 30, 35, 40, 45, and 50 Å is green, cyan, light magenta, yellow, salmon, gray, slate, orange, lime, dark teal, and hot pink, respectively).

In the NS5B^GT1b^/compound **2** system (Figure 20), the C2-position para-fluorophenyl of compound **2** similarly initially orients toward the binding pocket and guides the binding. Although residues Lys51, Asp220, and Asp319 successively hindered the binding of this inhibitor to NS5B^GT1b^ polymerase between distances of ~50 and ~25 Å, the interactions between compound **2** and residues Ser365, Cys366, and Leu384 allow the inhibitor to overcome this hindrance and gradually enter the binding pocket. Moving from a distance of ~25 to ~5 Å, the substituent at the C5 position of this inhibitor establishes crucial interactions with residues Met414 and Tyr448, while the N-methylmethanesulfonamide at the C6 position establishes important hydrophobic interactions with residue Pro197 and electrostatic interactions with the positively charged moiety of Arg200. Among them, the interaction established by compound **2** and Met414 promotes its C5 substituent to continuously penetrate the binding pocket and cross the reverse β-fold, ultimately orienting toward the cavity where residue Phe551 is located.

**Figure 21:**
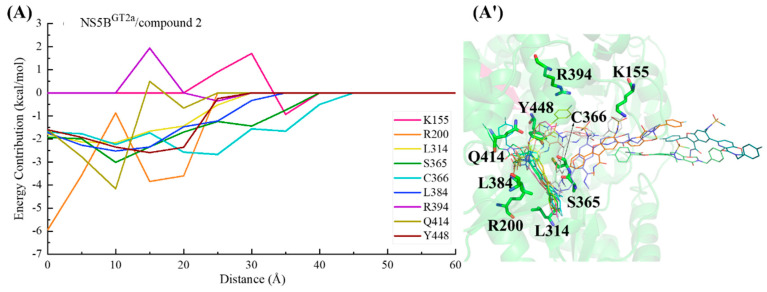
(**A**) Binding energy contributions of residues (>1.00 or <−2.00 kcal/mol) in the dissociation processes for the NS5B^GT2a^/compound **2** system (the distance of compound **2** from the equilibrium of the system is 0, 5, 10, 15, 20, 25, 30, 35, 40, 45, 50, 55, and 60 Å), and (**A’**) the binding path for compound **2** (the color of the structure at 0, 5, 10, 15, 20, 25, 30, 35, 40, and 45 Å is green, cyan, light magenta, yellow, salmon, gray, slate, orange, lime, and dark teal, respectively).

For the NS5B^GT2a^/compound **2** system (Figure 21), the C2-position para-fluorophenyl of the inhibitor first interacts with residues Cys366 and Ser365, driving the entire inhibitor into the binding pocket. Between a distance of ~25 and ~15 Å, the interaction between the C5 substituent of compound **2** and Tyr448 gradually strengthens, allowing the inhibitor to overcome the hindrance posed by residues Lys155 and Arg394 and continuously approach the reverse β-fold. Moving from a distance of ~15 to ~5 Å, the crucial interaction established between the C5 substituent and Gln414 guides it across the reverse β-fold, leading the inhibitor to complete binding to NS5B^GT2a^ polymerase, with the C5 substituent oriented toward the cavity where residue Phe551 is located.

**Figure 22:**
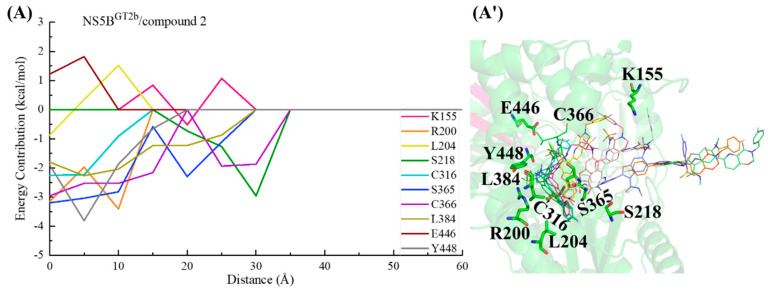
(**A**) Binding energy contributions of residues (>1.00 or <−2.00 kcal/mol) in the dissociation processes for the NS5B^GT2b^/compound **2** system (the distance of compound **2** from the equilibrium of the system is 0, 5, 10, 15, 20, 25, 30, 35, 40, 45, 50, 55, and 60 Å), and (**A’**) the binding path for compound **2** (the color of the structure at 0, 5, 10, 15, 20, 25, 30, 35, and 40 Å is green, cyan, light magenta, yellow, salmon, gray, slate, orange, and lime, respectively).

During the process of compound **2** binding to NS5B^GT2b^ polymerase (Figure 22), the para-fluorophenyl at the C2 position first interacts with Ser218 and Cys366, guiding the inhibitor into the binding pocket. Moving from a distance of ~30 to ~15 Å, the interaction of the 2-(4-fluorophenyl)-N-methyl-1-benzofuran-3-carboxamide moiety of compound **2** gradually approaches Ser365 and Leu384, inducing the inhibitor to penetrate deeper into the palm II subdomain of NS5B^GT2b^ polymerase. Moving from a distance from ~15 to ~5 Å, the interaction between the substituent at the C5 position and Tyr448 continues to strengthen, driving the inhibitor closer to the reverse β-fold. However, this process is hindered by Glu446. Under the combined action of Tyr448 and Glu446, compound **2** binds to NS5B^GT2b^ polymerase and its C5 substituent eventually orients toward the cavity where the residue Arg394 is located.

**Figure 23:**
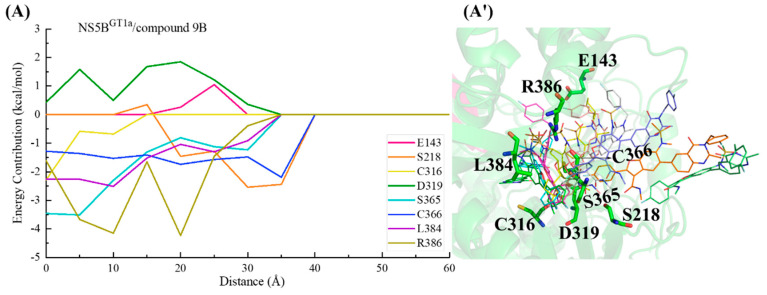
(**A**) Binding energy contributions of residues (>1.00 or <−2.00 kcal/mol) in the dissociation processes for the NS5B^GT1a^/compound **9B** system (the distance of compound **9B** from the equilibrium of the system is 0, 5, 10, 15, 20, 25, 30, 35, 40, 45, 50, 55, and 60 Å), and (**A’**) the binding path for compound **9B** (the color of the structure at 0, 5, 10, 15, 20, 25, 30, 35, and 40 Å is green, cyan, light magenta, yellow, salmon, gray, slate, orange, and lime, respectively).

In the NS5B^GT1a^/compound **9B** system (Figure 23), the C2 position para-fluorophenyl of compound **9B** first approaches residues Ser218 and Cys366 at the binding pocket entrance, and the established interactions guide the inhibitor into the binding pocket. Moving from a distance of ~35 to ~20 Å, compound **9B** gradually interacts with the residues Ser365, Leu384, and Arg386. Among them, residue Arg386 with positively charged groups is located near the N-methylethanesulfonamide of the inhibitor, making a prominent contribution to its binding to NS5B^GT1a^ polymerase. During this period, Glu143 near the C5 substituent and Asp319 near the C3 N-methylmethanamide of the inhibitor exhibit a certain repulsive effect on the binding of the inhibitor to NS5B^GT1a^ polymerase. Subsequently (from ~15 to ~5 Å), interactions between the substituent at the C5 position of compound **9B** and the key residue Cys316 induce the inhibitor to continuously move toward the reverse β-fold. Finally, after completing the binding to NS5B^GT1a^ polymerase, the C5 substituent of compound **9B** orients toward the cavity where residue Arg394 is located.

**Figure 24:**
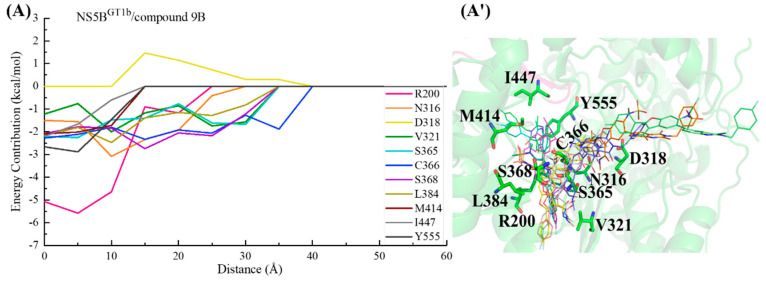
(**A**) Binding energy contributions of residues (>1.00 or <−2.00 kcal/mol) in the dissociation processes for the NS5B^GT1b^/compound **9B** system (the distance of compound **9B** from the equilibrium of the system is 0, 5, 10, 15, 20, 25, 30, 35, 40, 45, 50, 55, and 60 Å), and (**A’**) the binding path for compound **9B** (the color of the structure at 0, 5, 10, 15, 20, 25, 30, 35, and 40 Å is green, cyan, light magenta, yellow, salmon, gray, slate, orange, and lime, respectively).

During the binding process of compound **9B** with NS5B^GT1b^ polymerase (Figure 24), the para-fluorophenyl group at the C2 position first interacts with Cys366, guiding the inhibitor to bind. Moving from a distance of ~35 to ~15 Å, the inhibitor interacts with residues Val321, Ser365, Ser368, Leu384, Asn316, and Arg200, gradually going deeper into the binding pocket. During this period, residue Asp318 exerts a certain hindrance on the binding due to unfavorable polar solvation effects. Subsequently (from ~15 to~5 Å), interactions between compound **9B** and the residues Met414, Ile447, and Tyr555 near its C5 substituent are progressively strengthened, propelling the C5 substituent toward the reverse β-fold. With the assistance of the three residues mentioned above, the substituent at the C5 position crosses the reverse β-fold and ultimately orients toward the cavity where residue Phe551 is located.

**Figure 25:**
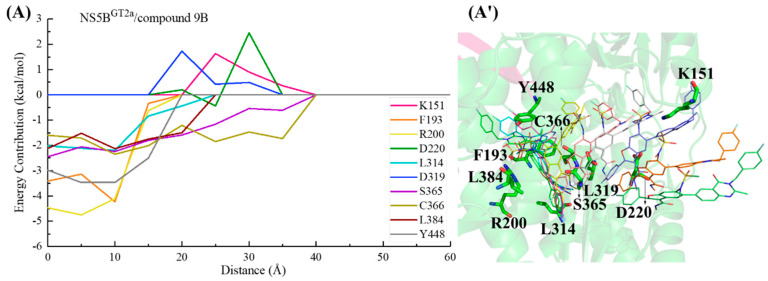
(**A**) Binding energy contributions of residues (>1.00 or <−2.00 kcal/mol) in the dissociation processes for the NS5B^GT2a^/compound **9B** system (the distance of compound **9B** from the equilibrium of the system is 0, 5, 10, 15, 20, 25, 30, 35, 40, 45, 50, 55, and 60 Å), and (**A’**) the binding path for compound **9B** (the color of the structure at 0, 5, 10, 15, 20, 25, 30, 35, and 40 Å is green, cyan, light magenta, yellow, salmon, gray, slate, orange, and lime, respectively).

For the NS5B^GT2a^/compound **9B** system (Figure 25), Moving from a distance from ~40 to ~25 Å, the para-fluorophenyl at the C2 position of compound **9B** mainly establishes critical interactions with residues Ser365 and Cys366, guiding the inhibitor into the binding pocket. Within this period, the binding is hindered by the entrance residues Lys151 and Asp220. Subsequently, from a distance of ~25 to ~10 Å, interactions between compound **9B** and the residues Leu384, Leu314, Tyr448, Arg200, and Phe193 gradually strengthen. Among them, residues Phe193 and Tyr448 near the C5 substituent induce the substituent to continuously approach the reverse β-fold. Under the influence of this effect, the substituent at the C5 position of the inhibitor traverses the reverse β-fold, moving toward the cavity where residue Phe551 is located, completing the binding.

**Figure 26:**
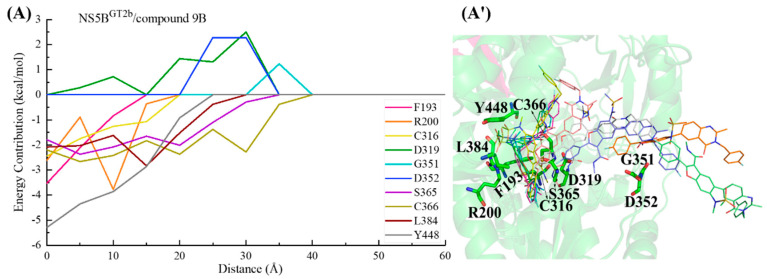
(**A**) Binding energy contributions of residues (>1.00 or <−2.00 kcal/mol) in the dissociation processes for the NS5B^GT2b^/compound **9B** system (the distance of compound **9B** from the equilibrium of the system is 0, 5, 10, 15, 20, 25, 30, 35, 40, 45, 50, 55, and 60 Å), and (**A’**) the binding path for compound **9B** (the color of the structure at 0, 5, 10, 15, 20, 25, 30, 35, and 40 Å is green, cyan, light magenta, yellow, salmon, gray, slate, orange, and lime, respectively).

For the NS5B^GT2b^/compound **9B** system (Figure 26), while moving from a distance of ~40 to ~20 Å, compound **9B** interacts successively with residues Cys366, Ser365, Leu384, and Tyr448, gradually guiding the inhibitor to bind. During this period, residues Gly351, Asp352, and Asp319 exert a certain degree of hindrance on the binding. Subsequently (from ~20 to ~10 Å), the interactions between compound **9B** and residues Arg200, Cys316, and Phe193 strengthen gradually. Among them, Phe193 and Cys316 are located on either side of the C5 substituent of the inhibitor. With the combined effect of residues Phe193, Cys316, and Tyr448, the C5 substituent of compound **9B** gradually approaches the reverse β-fold. Finally, after completing the binding to NS5B^GT2b^ polymerase, the C5 substituent of compound **9B** is oriented toward the cavity where residue Arg394 is located.

As analyzed above, when benzofuran core pan-genotypic inhibitors with long substituents at the C5 position (**BMS-929075**, **MK-8876**, compound **2**, and compound **9B**) bind to GT**1a**, **1b**, **2a**, and **2b** NS5B polymerases, interactions are first established by the para-fluorophenyl at the C2 position of the inhibitor with residues such as Cys366, Ser365, Lys155, Lys151, or Ser218 at the entrance of the binding pocket, and the recognition of the binding pocket is completed. During the binding process with GT1a and 2b NS5B polymerases, the interactions between the C5 substituent of the inhibitor and residues such as Tyr448 or Cys316 will guide the substituent into the cavity where residue Arg394 is located. In the process of binding to GT1b and 2a NS5B polymerases, the interactions between the C5 substituent and residues such as Phe193, Met^1b^/Gln^2a^414, or Arg200 will guide the substituent to cross the reverse β-fold (residues 441–456) and finally enter the cavity where residue Phe551 is located.

## 3. Materials and Methods

### 3.1. Preparation of Initial Models 

**Figure 27:**
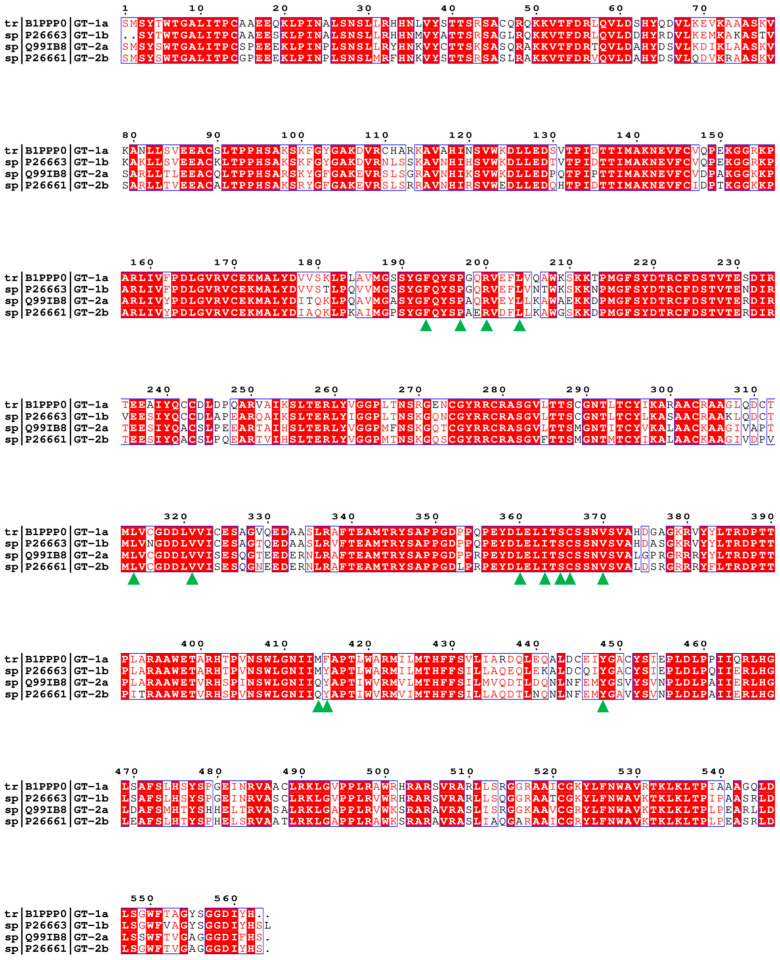
Multiple sequence alignment of four NS5B polymerases (GT**1a**, **1b**, **2a**, and **2b**). Residues conserved across all four genotypes are highlighted in red, while amino acid variations are shown on a white background. The green triangles indicate the crucial residues located within the binding site.

In this study, NS5B polymerases whose crystal structure has been characterized in the RCSB protein databank and who account for a large percentage of the global genotypes (GT**1a**, **1b**, **2a**, and **2b**) [26] were selected to study the mechanism of action of the five benzofuran core pan-genotypic inhibitors. To better illustrate the resemblance and differences between the target proteins, we performed a multiple sequence alignment of the four genotypes of NS5B polymerase (GT**1a**, GT**1b**, GT**2a**, and GT**2b**) using CLUSTALW and ESPript 3.0 (Figure 27). This alignment highlighted the key residues within the binding site, providing a clearer understanding of the conserved and variable regions among these genotypes. This detailed comparison underscores the basis for the interaction analyses and supports the rationale behind selecting specific residues for further investigation. Among them, NS5B^GT1b^ polymerase has corresponding co-crystal structures with **HCV-796**, **BMS-929075**, and **MK-8876** (their PDB codes are 3FQK [27], 5PZP [20], and 5W2E [16], respectively). In addition to the NS5B^GT1b^/**MK-8876** complex, complexes NS5B^GT1a^/**HCV-796**, NS5B^GT1a^/**BMS-929075**, NS5B^GT1a^/**MK-8876**, NS5B^GT1a^/compound **2**, NS5B^GT1a^/compound **9B**, NS5B^GT1b^/**HCV-796**, NS5B^GT1b^/**BMS-929075**, NS5B^GT1b^/compound **2**, NS5B^GT1b^/compound **9B**, NS5B^GT2a^/**HCV-796**, NS5B^GT2a^/**BMS-929075**, NS5B^GT2a^/**MK-8876**, NS5B^GT2a^/compound **2**, NS5B^GT2a^/compound **9B**, NS5B^GT2b^/**HCV-796**, NS5B^GT2b^/**BMS-929075**, NS5B^GT2b^/**MK-8876**, NS5B^GT2b^/compound **2**, and NS5B^GT2b^/compound **9B** were obtained through molecular docking using the Molecular Operating Environment 2019 (MOE 2019.0102) suite (Chemical Computing Group ULC, Montreal, QC, Canada). In the molecular docking process, the structures of GT1a and 2a NS5B polymerases co-crystallized with other benzofuran core inhibitors were used as the template structures for the corresponding molecular docking (the PDB IDs are 4KHR [28] and 5TWM [29], respectively). The newly reported NS5B^GT1b^/**MK-8876** co-crystal structure (PDB ID: 5W2E) was selected as the template structure of NS5B^GT1b^ polymerase for the molecular docking. Since NS5B^GT2b^ polymerase has no co-crystal structure with these five inhibitors, its wild-type crystal structure (PDB ID: 3GSZ [30]) was selected as the template for the docking. Before molecular docking, the mutant residues in the above crystal structures were reverse mutated to the corresponding wild-type residues. Then, missing atom completion, impurity ion deletion, protonation, and structure optimization were carried out. Since the ligands co-crystallized with GT**1a**, **1b**, and **2a** NS5B polymerases in the selected crystal structures are all benzofuran core inhibitors, the location of the original ligand was chosen as the docking site for the construction of the relevant complex systems. However, NS5B^GT2b^ polymerase does not contain co-crystallized ligands, so the key amino acid residues in the palm II subdomain (Phe193, Pro197, Arg200, Leu204, Leu314, Leu360, Ile363, Val321, Ser365, Cys366, Val370, Met^1a,1b^/Gln^2a,2b^414, Phe^1a^/Tyr^1b,2a,2b^415, and Tyr448) [25] were chosen to locate the docking site. The 3D structures of the five inhibitors were obtained by converting the 2D structures constructed in ChemDraw 20.0 software in MOE 2019 software. These structures were then protonated and optimized for the molecular docking. Specifically, the ligands were placed in the binding sites and optimized using the triangle matcher method. The top 100 conformations were initially screened according to the London dG scoring function. Subsequently, the induced-fit docking method was employed to further refine the top ten ligands from the first step using the GBVI/WSA dG (Generalized-Born Volume Integral/Weighted Surface Area dG) force-field-based scoring function. The best conformation was then selected based on these refinements.

To validate the accuracy of our molecular docking results, the docking poses were superimposed onto the corresponding crystallographic structures, and the RMSD values of the docking structures relative to the crystallographic structures were calculated (Table S1). The low RMSD values indicated a high degree of similarity between the docking poses and the crystallographic structures, supporting the validity of our docking methodology and confirming the consistency of the predicted binding modes with the crystallographic structures.

### 3.2. Molecular Dynamics Simulations

First, the tleap module of AMBER 18 (University of California, San Francisco, CA, USA) was used to generate the topology/coordinate/parameter files of the receptors, ligands and complexes in the above constructed complex systems using FF14SB [31] and GAFF [32] force fields. The electrostatic potential (ESP) of the ligands was computed at the HF/6-31G* level using Gaussian 09 [33] software, followed calculating the Restrained Electrostatic Potential (RESP) charges [34] using the Antechamber [35] module. Each system was solvated within a cube box of TIP3P [36] water molecules with a 12 Å distance around the solute. In addition, Cl^-^ counterions were added to the solution to make the system electrically neutral.

Before running the MD simulations, energy minimization of each system was carried out in two steps: first, the receptor backbone and ligand atoms were restrained, and the water molecules and ions were relaxed for 5000 cycles (2500 cycles of steepest descent and 2500 cycles of conjugate gradient minimizations). Next, the whole system was minimized for 5000 cycles without any restraints (2500 cycles of steepest descent and 2500 cycles of conjugate gradient minimizations). After minimization, the systems were gradually heated up from 0 K to 310 K in seven steps under the NPT ensemble. When the systems reached equilibrium, a 100 ns MD simulation was carried out at a constant temperature of 310 K for each system. The time step and nonbonded cutoff were set to 2.0 fs and 10 Å, respectively. During the MD simulations, the SHAKE algorithm [37] was used to deal with the bonds connected to the hydrogen atoms, and the particle mesh Ewald (PME) method [38] was employed to calculate the long-range electrostatic interactions.

### 3.3. Binding Free Energy Calculations and Energy Decomposition Analyses

The binding free energy of each system was calculated using the MM/GBSA method according to the following equation [39]:

$$∆G_{\mathrm{bind}}=∆G_{\mathrm{complex}}-\left(∆G_{\mathrm{protein}}+∆G_{\mathrm{ligand}}\right)=∆E_{\mathrm{gas}}+∆G_{\mathrm{sol}}-T∆S\;∆E_{\mathrm{gas}}=∆E_{\mathrm{vdw}}+∆E_{\mathrm{ele}}+∆E_{\mathrm{int}},\;∆G_{\mathrm{sol}}=∆G_{\mathrm{GB}}+∆G_{\mathrm{SA}}$$

where $∆E_{\mathrm{gas}}$ represents the gas-phase interaction energy between the receptor and the ligand, including $∆E_{\mathrm{vdw}}$ (van der Waal energy) and $∆E_{\mathrm{ele}}$ (electrostatic energy) and $∆E_{\mathrm{int}}$ (internal energy). In the single trajectory MD simulations, $∆E_{\mathrm{int}}$ is usually eliminated [40]. $∆G_{\mathrm{sol}}$ stands for the desolvation energy and consists of two parts, $∆G_{\mathrm{GB}}$ (polar desolvation energy) and $∆G_{\mathrm{SA}}$ (non-polar desolvation energy). −T∆S refers to the contribution of configurational entropy to the binding free energy at temperature T. Here, $∆G_{\mathrm{GB}}$ was calculated using the Generalized Born (GB) model [41], and $∆G_{\mathrm{SA}}$ was calculated using the LCPO method [42]. Since the structures of the compounds in this work are relatively similar and the normal mode of calculation is very time-consuming [43,44,45], we ignored it here. To deeply investigate the binding mode and action details of each inhibitor with different genotypes of NS5B polymerase, the energy contribution of each residue to the binding of the inhibitor with NS5B polymerase was obtained through energy decomposition calculations. The energy contribution is mainly composed of four energy terms: the van der Waals contribution ($∆E_{\mathrm{vdw}}$), electrostatic contribution ($∆E_{\mathrm{ele}}$), polar contribution of desolvation ($∆G_{\mathrm{GB}}$), and non-polar contribution ($∆G_{\mathrm{SA}}$).

### 3.4. Adaptive Steered Molecular Dynamics (ASMD) Simulations and Potential of Mean Force (PMF) Calculations

Before the ASMD simulations, CAVER 3.0 software [46] was used to analyze the optimal unbinding channel. Then, ASMD simulations [47,48] and PMF calculations [49] were performed on the twenty complex systems that had reached a steady state in the molecular dynamics simulations. In the ASMD simulations, the predetermined reaction coordinates, i.e., the distance between the receptor and the ligand, were divided into stages. In each stage, multiple simulations were performed in parallel with the same starting structure and the last structure of the trajectory whose work value was closest to the Jarzynski average (JA) [50] was used as the starting structure for the next stage. Finally, the PMF calculations were performed using the JA trajectories selected in each stage. Specifically, the distance between the inhibitor and NS5B in the equilibrium structure of the complexes was set to 0 Å, and the stretching distance was set to 60 Å. The reaction coordinate was split into 12 stages, and 20 parallel simulations were performed for each stage. The stretching velocity and force constant were set to 10 Å/ns and 7.2 kcal/(mol/Å^2^), respectively. Additionally, the cartoon models were generated using the PyMOL 2.3.1 software [51].

## 4. Conclusions

In the present study, in order to elucidate the detailed binding mechanisms of five benzofuran core pan-genotypic HCV NS5B polymerase inhibitors (**HCV-796**, **BMS-929075**, **MK-8876**, compound **2**, and compound **9B**) with great potential as drugs, the pan-genotypic (GT**1a**, **1b**, **2a**, and **2b**) action modes of these inhibitors were thoroughly studied through a series of molecular simulation methods.

Our calculations showed that these five benzofuran inhibitors can not only establish extensive interactions with residues in the palm II subdomain, but they also interact with residues in the palm I subdomain or palm I/III overlap region after binding to the four genotypes of NS5B polymerase. Interestingly, the benzofuran inhibitors with longer substituents at the C5 position (**BMS-929075**, **MK-8876**, compound **2**, and compound **9B**) are oriented toward the cavity where Arg394 is located after binding to GT1a and 2b NS5B polymerases, and toward the cavity where Phe551 is located after binding GT1b and 2a NS5B polymerases. The ASMD simulations showed that when these four inhibitors bind to the four genotypes of NS5B polymerase, and the interactions are established initially by the para-fluorophenyl at the C2 position of the inhibitor with residues such as Cys366, Ser365, Lys155, Lys151, or Ser218 at the entrance of the binding pocket, completing the recognition of the binding pocket. During the process of binding to GT1a and 2b NS5B polymerases, the interactions between the C5 substituent of the inhibitor and residues such as Tyr448 or Cys316 will guide the substituent into the cavity where residue Arg394 is located. In the process of binding to GT1b and 2a NS5B polymerases, the interactions between the C5 substituent and residues such as Phe193, Met^1b^/Gln^2a^414, or Arg200 will guide the substituent to cross the reverse β-fold (residues 441–456) and finally enter the cavity where residue Phe551 is located. Additionally, a description of common residues contributing to the binding of benzofuran core inhibitors and the differences in target residues or compound substituents are summarized in the Supplementary Materials.

These research findings clearly revealed the detailed mechanisms of action of benzofuran HCV NS5B polymerase pan-genotypic inhibitors, providing valuable insights for the further research and development of novel benzofuran-based antiviral agents targeting a broad spectrum of HCV genotypes.

Future research should focus on expanding the analysis to additional HCV genotypes as structural data become available, and conducting experimental validations such as binding assays and crystallographic studies. These efforts will enhance the understanding of the pan-genotypic efficacy of benzofuran inhibitors and support the development of more effective NS5B polymerase inhibitors.

## Figures and Tables

**Table 1:** The binding free energies and the individual energy terms for each complex system predicted by the MM/GBSA method (kcal/mol).

System	$∆E_{\mathrm{ele}}$ ^a^	$∆E_{\mathrm{vdw}}$ ^b^	$∆G_{\mathrm{GB}}$ ^c^	$∆G_{\mathrm{SA}}$ ^d^	$(∆E_{\mathrm{ele}}+∆G_{\mathrm{GB}})$ ^e^	$∆G_{\mathrm{sol}}$ ^f^	$∆G_{\mathrm{bind}}$ ^g^
NS5B^GT1a^/**HCV-796**	−22.21 ± 7.41	−59.56 ± 3.02	35.42 ± 5.30	−7.50 ± 0.19	13.21 ± 9.11	27.93 ± 5.26	−53.83 ± 4.20
NS5B^GT1b^/**HCV-796**	−41.68 ± 7.03	−62.15 ± 2.95	46.79 ± 3.32	−7.81 ± 0.14	5.11 ± 7.77	38.98 ± 3.31	−64.85 ± 4.86
NS5B^GT2a^/**HCV-796**	−36.65 ± 5.27	−61.27 ± 2.94	44.40 ± 3.90	−7.92 ± 0.18	7.75 ± 6.56	36.48 ± 3.87	−61.44 ± 3.62
NS5B^GT2b^/**HCV-796**	−43.44 ± 6.98	−63.13 ± 3.21	46.67 ± 3.57	−8.12 ± 0.13	3.23 ± 7.84	38.54 ± 3.56	−68.03 ± 4.71
NS5B^GT1a^/**BMS-929075**	−15.33 ± 3.85	−64.84 ± 2.71	36.43 ± 3.06	−7.78 ± 0.21	21.10 ± 4.92	28.64 ± 3.05	−51.53 ± 2.81
NS5B^GT1b^/**BMS-929075**	−32.10 ± 3.30	−73.73 ± 3.14	50.57 ± 2.86	−9.17± 0.22	18.47 ± 4.37	41.40 ± 2.81	−64.43 ± 3.46
NS5B^GT2a^/**BMS-929075**	−33.57 ± 4.48	−76.74 ± 3.02	54.33 ± 2.79	−9.55 ± 0.14	20.76 ± 5.28	44.78 ± 2.78	−65.53 ± 3.56
NS5B^GT2b^/**BMS-929075**	−10.80 ± 4.59	−61.87 ± 3.26	32.49 ± 4.09	−7.79 ± 0.28	21.69 ± 6.15	24.70 ± 4.07	−47.97 ± 3.19
NS5B^GT1a^/**MK-8876**	−31.81 ± 5.67	−75.47 ± 3.39	48.27 ± 3.20	−8.81 ± 0.18	16.46 ± 6.51	39.46 ± 3.27	−67.82 ± 4.34
NS5B^GT1b^/**MK-8876**	−39.49 ± 4.21	−84.31 ± 3.09	57.78 ± 3.19	−9.98 ± 0.16	18.29 ± 5.28	47.81 ± 3.18	−75.99 ± 3.36
NS5B^GT2a^/**MK-8876**	−35.83 ± 3.76	−83.43 ± 3.10	52.47 ± 2.87	−9.88 ± 0.14	16.64 ± 4.73	42.59 ± 2.85	−76.67 ± 3.49
NS5B^GT2b^/**MK-8876**	−28.53 ± 5.22	−72.69 ± 3.43	46.69 ± 3.31	−8.62 ± 0.24	18.16 ± 6.18	38.07 ± 3.27	−63.15 ± 4.76
NS5B^GT1a^/compound **2**	−21.63 ± 7.45	−71.57 ± 5.82	48.06 ± 6.00	−8.60 ± 0.56	26.43 ± 9.57	39.46 ± 5.97	−53.74 ± 7.72
NS5B^GT1b^/compound **2**	−39.79 ± 3.86	−87.35 ± 3.15	57.53 ± 3.03	−10.58 ± 0.18	17.74 ± 4.91	46.95 ± 3.02	−80.18 ± 3.49
NS5B^GT2a^/compound **2**	−47.00 ± 4.34	−85.22 ± 2.95	64.66 ± 3.20	−10.42 ± 0.17	17.66 ± 5.39	54.24 ± 3.17	−77.99 ± 3.83
NS5B^GT2b^/compound **2**	−32.81 ± 7.14	−76.52 ± 3.86	47.25 ± 5.65	−9.17 ± 0.30	14.44 ± 9.11	38.08 ± 5.71	−71.24 ± 4.54
NS5B^GT1a^/compound **9B**	−13.92 ± 6.99	−68.33 ± 4.02	38.72 ± 5.57	−8.44 ± 0.36	24.8 ± 8.94	30.28 ± 5.49	−51.97 ± 4.18
NS5B^GT1b^/compound **9B**	−36.08 ± 3.84	−92.10 ± 3.18	56.97 ± 3.69	−10.94 ± 0.17	20.89 ± 5.33	46.03 ± 2.66	−82.14 ± 3.48
NS5B^GT2a^/compound **9B**	−44.38 ± 4.33	−93.35 ± 3.34	63.32 ± 3.08	−10.58 ± 0.16	18.94 ± 5.31	52.74 ± 3.07	−84.98 ± 3.79
NS5B^GT2b^/compound **9B**	−31.47 ± 4.32	−84.10 ± 3.45	51.52 ± 3.07	−9.87 ± 0.16	20.05 ± 5.30	41.65 ± 3.05	−73.91 ± 3.82

^a^ electrostatic contribution; ^b^ van der Waals contribution; ^c^ the polar contribution of desolvation; ^d^ the nonpolar contribution of desolvation; ^e^ the net electrostatic contribution; ^f^ the desolvation energy; ^g^ the predicted binding free energy.

**Table 2:** Hydrogen bonds formed between the benzofuran core inhibitors and the four genotypes of NS5B polymerase during the last 20 ns in the MD simulations.

System	Donor	Acceptor	Occupied (%)	Distance (Å)	Angle (deg)
NS5B^GT1a^/**HCV-796**	Ser365@OG	**HCV-796**@O2	70.81	2.75	156.77
	Arg200@NH1	**HCV-796**@O3	24.97	2.89	151.74
NS5B^GT1b^/**HCV-796**	Tyr448@OH	**HCV-796**@O4	63.34	2.81	164.68
	Tyr415@OH	**HCV-796**@O3	52.39	2.76	160.03
	Arg200@NH1	**HCV-796**@O2	44.95	2.85	149.42
	Asn316@ND2	**HCV-796**@O2	25.90	2.87	156.13
NS5B^GT2a^/**HCV-796**	Gln414@NE2	**HCV-796**@O4	73.29	2.83	159.52
	Arg200@NH2	**HCV-796**@O5	67.65	2.83	150.34
NS5B^GT2b^/**HCV-796**	Arg200@NH2	**HCV-796**@O5	75.10	2.82	150.20
	Arg200@NE	**HCV-796**@O5	52.91	2.86	149.51
	Tyr415@OH	**HCV-796**@O3	37.17	2.76	160.12
NS5B^GT1a^/**BMS-929075**	Ser365@OG	**BMS-929075**@O2	87.00	2.74	158.40
NS5B^GT1b^/**BMS-929075**	Arg200@NH1	**BMS-929075**@O2	76.92	2.80	151.47
NS5B^GT2a^/**BMS-929075**	Gln414@NE2	**BMS-929075**@O3	66.85	2.86	157.30
	Arg200@NH2	**BMS-929075**@O3	59.63	2.85	150.75
NS5B^GT2b^/**BMS-929075**	Ser365@OG	**BMS-929075**@O2	94.14	2.70	159.35
NS5B^GT1a^/**MK-8876**	Arg200@NH2	**MK-8876**@O5	66.27	2.83	154.21
	Ser365@OG	**MK-8876**@O3	51.14	2.77	155.59
	Tyr448@OH	**MK-8876**@O4	41.49	2.75	162.82
NS5B^GT1b^/**MK-8876**	Arg200@NE	**MK-8876**@O5	59.05	2.86	152.30
	Arg200@NH2	**MK-8876**@O5	56.52	2.85	149.85
NS5B^GT2a^/**MK-8876**	Ser365@OG	**MK-8876**@O3	75.67	2.76	156.90
	Arg200@NH1	**MK-8876**@O5	53.52	2.86	152.22
	Arg200@NE	**MK-8876**@O5	45.36	2.88	153.68
NS5B^GT2b^/**MK-8876**	Tyr448@OH	**MK-8876**@O4	69.77	2.76	161.14
	Arg200@NH2	**MK-8876**@O5	25.69	2.82	150.93
	Arg200@NH1	**MK-8876**@O5	23.52	2.82	151.63
	Ser365@OG	**MK-8876**@O3	4.60	2.79	152.98
NS5B^GT1a^/compound **2**	Ser365@OG	compound **2**@O4	30.33	2.79	153.92
NS5B^GT1b^/compound **2**	Arg200@NH1	compound **2**@O2	69.64	2.84	154.15
	Arg200@NH1	compound **2**@O6	57.19	2.86	151.57
NS5B^GT2a^/compound **2**	Arg200@NE	compound **2**@O6	43.99	2.89	153.96
	Arg200@NH1	compound **2**@O6	35.68	2.86	150.18
NS5B^GT2b^/compound **2**	Tyr448@OH	compound **2**@O5	81.31	2.75	160.11
	Arg200@NE	compound **2**@O6	60.94	2.85	151.81
	Ser365@OG	compound **2**@O4	55.35	2.77	154.42
	Arg200@NH2	compound **2**@O6	45.01	2.84	148.11
NS5B^GT1a^/compound **9B**	Ser365@OG	compound **9B**@O3	57.09	2.77	156.50
NS5B^GT1b^/compound **9B**	Arg200@NH1	compound **9B**@O3	47.58	2.83	146.21
	Asn316@ND2	compound **9B**@O3	20.86	2.85	161.70
NS5B^GT2a^/compound **9B**	Arg200@NH1	compound **9B**@O5	63.50	2.84	149.45
	Arg200@NH1	compound **9B**@O1	36.93	2.82	145.36
NS5B^GT2b^/compound **9B**	Tyr448@OH	compound **9B**@O4	80.70	2.77	161.65
	Arg200@NH2	compound **9B**@O5	47.47	2.82	152.72

**Table 3:** Characteristics of the two top ranked pathways of NS5B^GT1a^/**MK-8876**, NS5B^GT1b^/**MK-8876**, NS5B^GT2a^/**MK-8876**, and NS5B^GT2b^/**MK-8876** complexes.

System	Pathway 1			Pathway 2		
	Bottleneck Radius (Å)	Length (Å)	Curvature (Å)	Bottleneck Radius (Å)	Length (Å)	Curvature (Å)
NS5B^GT1a^/**MK-8876**	4.60	14.81	1.06	2.25	19.13	1.13
NS5B^GT1b^/**MK-8876**	3.29	11.39	1.22	1.49	26.04	1.46
NS5B^GT2a^/**MK-8876**	3.11	9.73	1.18	2.20	21.85	1.24
NS5B^GT2b^/**MK-8876**	3.44	11.81	1.26	1.73	26.72	1.33

## Data Availability

All data are contained within the article and Supplementary Materials.

## Supplementary Materials

The following supporting information can be downloaded at: https://www.mdpi.com/article/10.3390/ijms25158028/s1.
