# Supplementary material for: Distinct Characteristic Binding Modes of Benzofuran Core Inhibitors to Diverse Genotypes of Hepatitis C Virus NS5B Polymerase: A Molecular Simulation Study
Source: Int J Mol Sci. 2024 Jul 23;25(15):8028. doi: 10.3390/ijms25158028 (PMC11311972; doi:10.3390/ijms25158028)
Supplement: Supplementary file 1 [file ijms-25-08028-s001.zip › ijms-3093670-supplementary.pdf]

# Distinct characteristic binding modes of benzofuran core inhibitors to diverse genotypes of NS5B polymerase: Implications for pan-genotypic HCV inhibition via a molecular simulation study

Di Han[*,‡,1,2,3], Fang Zhao[‡,1,2,3], Yifan Chen[1,2,3], Yiwei Xue[1,2,3], Ke Bao[1,2,3], Yuxiao Chang[1,2,3], Jiarui Lu[1,2,3], Meiting Wang[1,2,3], Taigang Liu[1,2,3], Qinghe Gao[4], Wei Cui[5], Yongtao Xu[**,1,2,3]

[1] School of Medical Engineering, Xinxiang Medical University, Xinxiang, Henan, 453003, China

[2] Henan International Joint Laboratory of Neural Information Analysis and Drug Intelligent Design, Xinxiang, Henan, 453003, China

[3] Xinxiang Key Laboratory of Biomedical Information Research, Xinxiang, Henan, 453003, China

[4] School of Pharmacy, Xinxiang Medical University, Xinxiang, 453003, China

[5] School of Chemical Sciences, University of Chinese Academy of Sciences, No. 19A, YuQuan Road, Beijing, 100049, China

[*] Author for correspondence: hd@xxmu.edu.cn

[**] Author for correspondence: yxu@xxmu.edu.cn

[‡] Authors contributed equally to this work

## Contents


Table S1. The best docking pose scores and the RMSD values of the docking structures relative to the crystallographic structures ........ 3

Figure S1. The root-mean-square deviations (RMSD) of the ligands in the 20 complex systems. ........ 5

Figure S2. The individual energy term contributions of key residues to the binding free energies of (A) NS5B$^{GT1a}$/HCV-796, (B) NS5B$^{GT1b}$/HCV-796, (C) NS5B$^{GT2a}$/HCV-796, and (D) NS5B$^{GT2b}$/HCV-796 systems. ........ 6

Figure S3. The energetic difference ($\Delta\Delta G = \Delta G$NS5B$^{GT1b/GT2a/GT2b}$/inhibitor - $\Delta G$NS5B$^{GT1a}$/inhibitor) spectra of the corresponding key residues between the NS5B$^{GT1a}$ system and the NS5B$^{GT1b}$ system, NS5B$^{GT2a}$ system, or NS5B$^{GT2b}$ system. (A) NS5B/HCV-796; (B) NS5B/BMS-929075; (C) NS5B/MK-8876; (D) NS5B/compound 2; (E) NS5B/compound 9B. ........ 7

Figure S4. Distances between HCV-796 and residues during the last 20 ns MD trajectories. .. 8

Figure S5. The energetic difference spectra between the NS5B$^{GT1a}$/HCV-796 system and the NS5B$^{GT1a}$/BMS-929075, NS5B$^{GT1a}$/MK-8876, NS5B$^{GT1a}$/compound 2 or NS5B$^{GT1a}$/compound 9B system ($\Delta\Delta G = \Delta G$NS5B$^{GT1a}$/(BMS-929075/MK-8876/compound 2/compound 9B) - $\Delta G$NS5B$^{GT1a}$/HCV-796). ........ 9

Figure S6. The binding modes of (A) NS5B$^{GT1a}$/HCV-796 versus NS5B$^{GT1a}$/BMS-929075, (B) NS5B$^{GT1a}$/HCV-796 versus NS5B$^{GT1a}$/MK-8876, (C) NS5B$^{GT1a}$/HCV-796 versus NS5B$^{GT1a}$/compound 2, and (D) NS5B$^{GT1a}$/HCV-796 versus NS5B$^{GT1a}$/compound 9B. ........ 10

Figure S7. Distances between BMS-929075 and residues during the last 20 ns MD trajectories. ........ 11

Figure S8. The tunnels represented by residues Arg394 (skyblue) and Phe551 (warmpink) for (A) NS5B$^{GT1a}$/BMS-929075 (slate), (B) NS5B$^{GT1b}$/BMS-929075 (salmon), (C) NS5B$^{GT2a}$/BMS-929075 (yellow orange) and (D) NS5B$^{GT2b}$/BMS-929075 (cyan). ........ 12

Figure S9. The individual energy term contributions of key residues to the binding free energies of (A) NS5B$^{GT1a}$/BMS-986139, (B) NS5B$^{GT1b}$/BMS-986139, (C) NS5B$^{GT2a}$/BMS-986139, and (D) NS5B$^{GT2b}$/BMS-986139 system. ........ 13

Figure S10. The tunnels represented by residues Arg394 (skyblue) and Phe551 (warmpink) for (A) NS5B$^{GT1a}$/MK-8876 (cyan), (B) NS5B$^{GT1b}$/MK-8876 (orange), (C) NS5B$^{GT2a}$/MK-8876 (salmon) and (D) NS5B$^{GT2b}$/MK-8876 (limon). ........ 14

Figure S11. Distances between MK-8876 and residues during the last 20 ns MD trajectories. ........ 15

Figure S12. The individual energy term contributions of key residues to the binding free energies of (A) NS5B$^{GT1a}$/MK-8876, (B) NS5B$^{GT1b}$/MK-8876, (C) NS5B$^{GT2a}$/MK-8876, and (D) NS5B$^{GT2b}$/MK-8876 system. .......... 16

Figure S13. The tunnels represented by residues Arg394 (skyblue) and Phe551 (warmpink) for (A) NS5B$^{GT1a}$/compound 2 (green), (B) NS5B$^{GT1b}$/compound 2 (cyan), (C) NS5B$^{GT2a}$/compound 2 (orange) and (D) NS5B$^{GT2b}$/compound 2 (yellow). .......... 17

Figure S14. Distances between compound 2 and residues during the last 20 ns MD trajectories. .......... 18

Figure S15. The individual energy term contributions of key residues to the binding free energies of (A) NS5B$^{GT1a}$/compound 2, (B) NS5B$^{GT1b}$/compound 2, (C) NS5B$^{GT2a}$/compound 2 and (D) NS5B$^{GT2b}$/compound 2 system. .......... 19

Figure S16. The tunnels represented by residues Arg394 (skyblue) and Phe551 (warmpink) for (A) NS5B$^{GT1a}$/compound 9B (orange), (B) NS5B$^{GT1b}$/compound 9B (slate), (C) NS5B$^{GT2a}$/compound 9B (limon) and (D) NS5B$^{GT2b}$/compound 9B (yellow). .......... 20

Figure S17. Distances between compound 9B and residues during the last 20 ns MD trajectories. .......... 21

Figure S18. The individual energy term contributions of key residues to the binding free energies of (A) NS5B$^{GT1a}$/compound 9B, (B) NS5B$^{GT1b}$/compound 9B, (C) NS5B$^{GT2a}$/compound 9B and (D) NS5B$^{GT2b}$/compound 9B system. .......... 22

Summary of Common Residues Contributing to the Binding of Benzofuran Core Inhibitors 23

Highlight of Differences in Target Residues or Compound Substituents for Designing More Efficient or Selective Compounds .......... 25

## Table S1. The best docking pose scores and the RMSD values of the docking structures relative to the crystallographic structures

| COMPLEX | Score(kcal/mol) | RMSD | Reference Structure for the RMSD Calculation |
|---|---|---|---|
| NS5B$^{GT1a}$/HCV-796 | -10.82 | 0.264 | 4KHR |
| NS5B$^{GT1a}$/BMS-929075 | -10.66 | 0.264 | 4KHR |
| NS5B$^{GT1a}$/MK-8876 | -11.58 | 0.264 | 4KHR |
| NS5B$^{GT1a}$/compound 2 | -12.54 | 0.264 | 4KHR |
| NS5B$^{GT1a}$/compound 9B | -12.53 | 0.264 | 4KHR |
| NS5B$^{GT1b}$/HCV-796 | -11.11 | 0.218 | 3FQK |
| NS5B$^{GT1b}$/BMS-929075 | -12.48 | 0.390 | 5PZP |
| NS5B$^{GT1b}$/MK-8876 | -13.68 | 0.161 | 5W2E |
| NS5B$^{GT1b}$/compound 2 | -13.91 | 0.161 | 5W2E |

| | | | |
|---|---|---|---|
| NS5B$^{GT1b}$/compound 9B | -14.04 | 0.161 | 5W2E |
| NS5B$^{GT2a}$/HCV-796 | -11.51 | 0.154 | 5TWM |
| NS5B$^{GT2a}$/BMS-929075 | -12.97 | 0.154 | 5TWM |
| NS5B$^{GT2a}$/MK-8876 | -13.98 | 0.154 | 5TWM |
| NS5B$^{GT2a}$/compound 2 | -14.41 | 0.154 | 5TWM |
| NS5B$^{GT2a}$/compound 9B | -14.76 | 0.154 | 5TWM |
| NS5B$^{GT2b}$/HCV-796 | -11.05 | 0.160 | 3GSZ |
| NS5B$^{GT2b}$/BMS-929075 | -11.13 | 0.160 | 3GSZ |
| NS5B$^{GT2b}$/MK-8876 | -12.10 | 0.160 | 3GSZ |
| NS5B$^{GT2b}$/compound 2 | -12.45 | 0.160 | 3GSZ |
| NS5B$^{GT2b}$/compound 9B | -12.36 | 0.160 | 3GSZ |

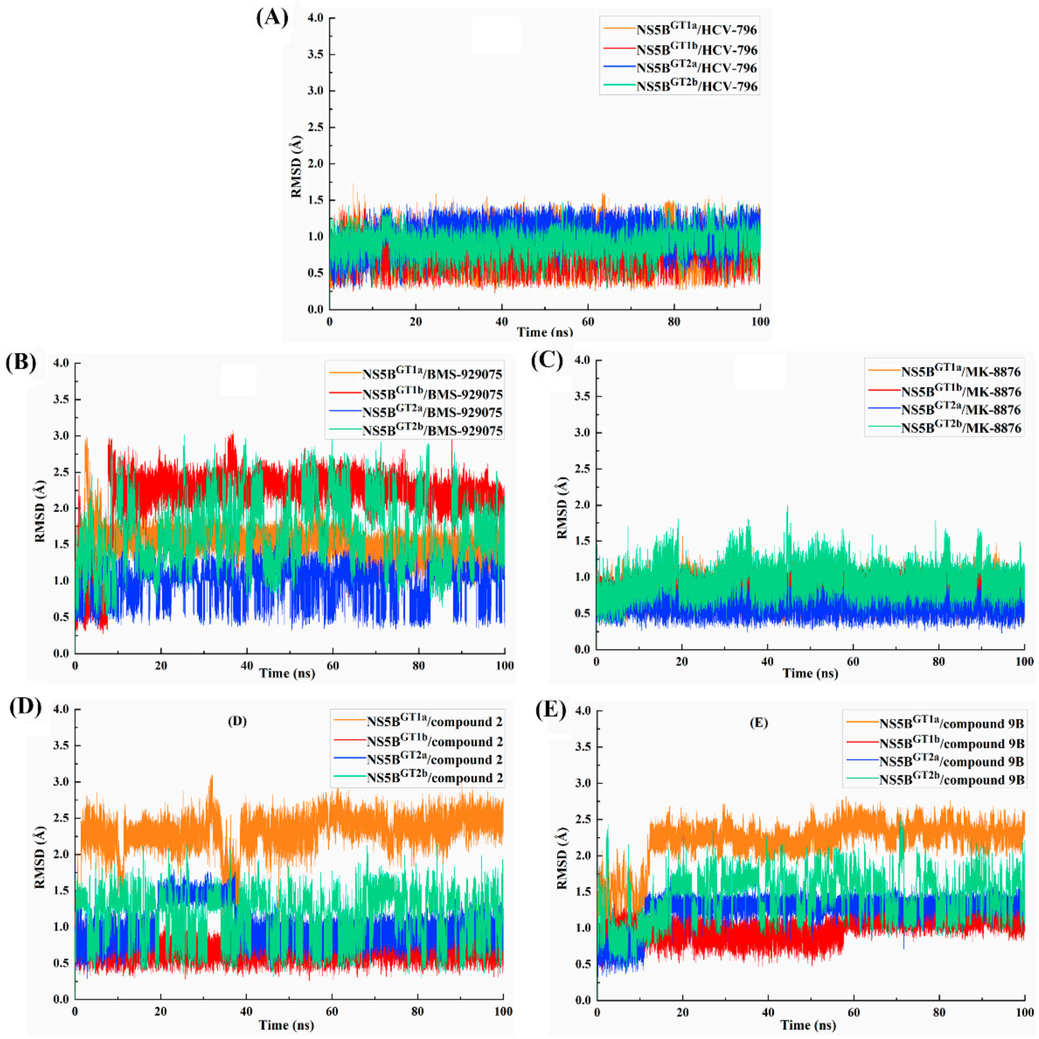


Figure S1. The root-mean-square deviations (RMSD) of the ligands in the 20 complex systems.

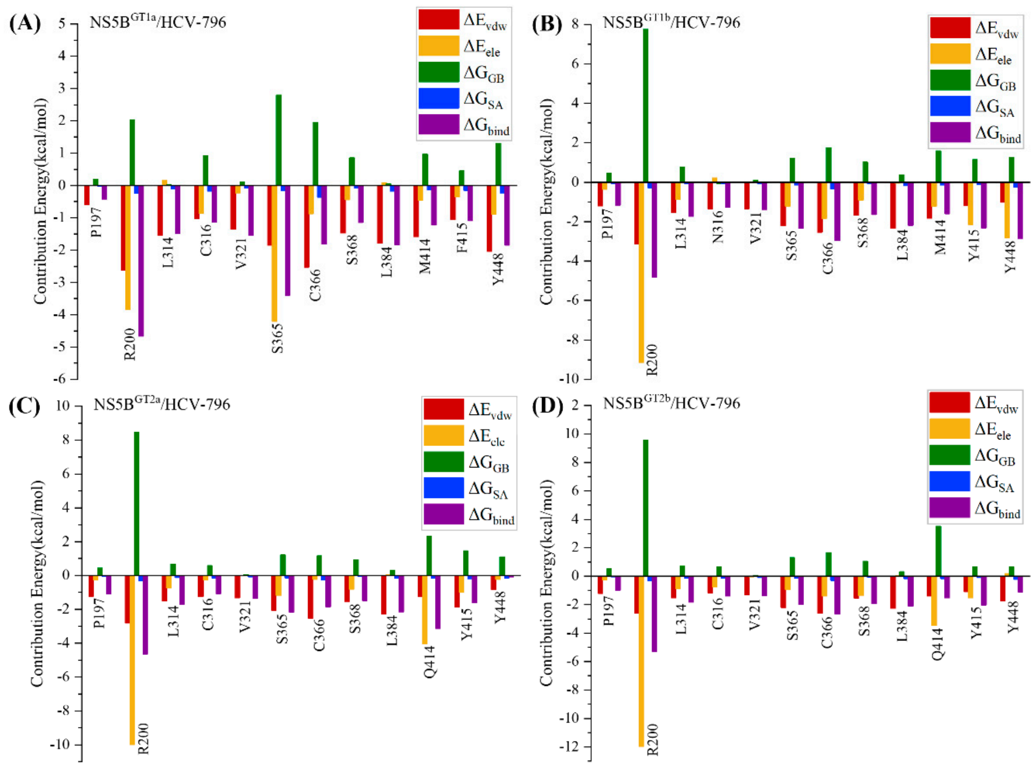


Figure S2. The individual energy term contributions of key residues to the binding free energies of (A) NS5B$^{GT1a}$/HCV-796, (B) NS5B$^{GT1b}$/HCV-796, (C) NS5B$^{GT2a}$/HCV-796, and (D) NS5B$^{GT2b}$/HCV-796 systems.

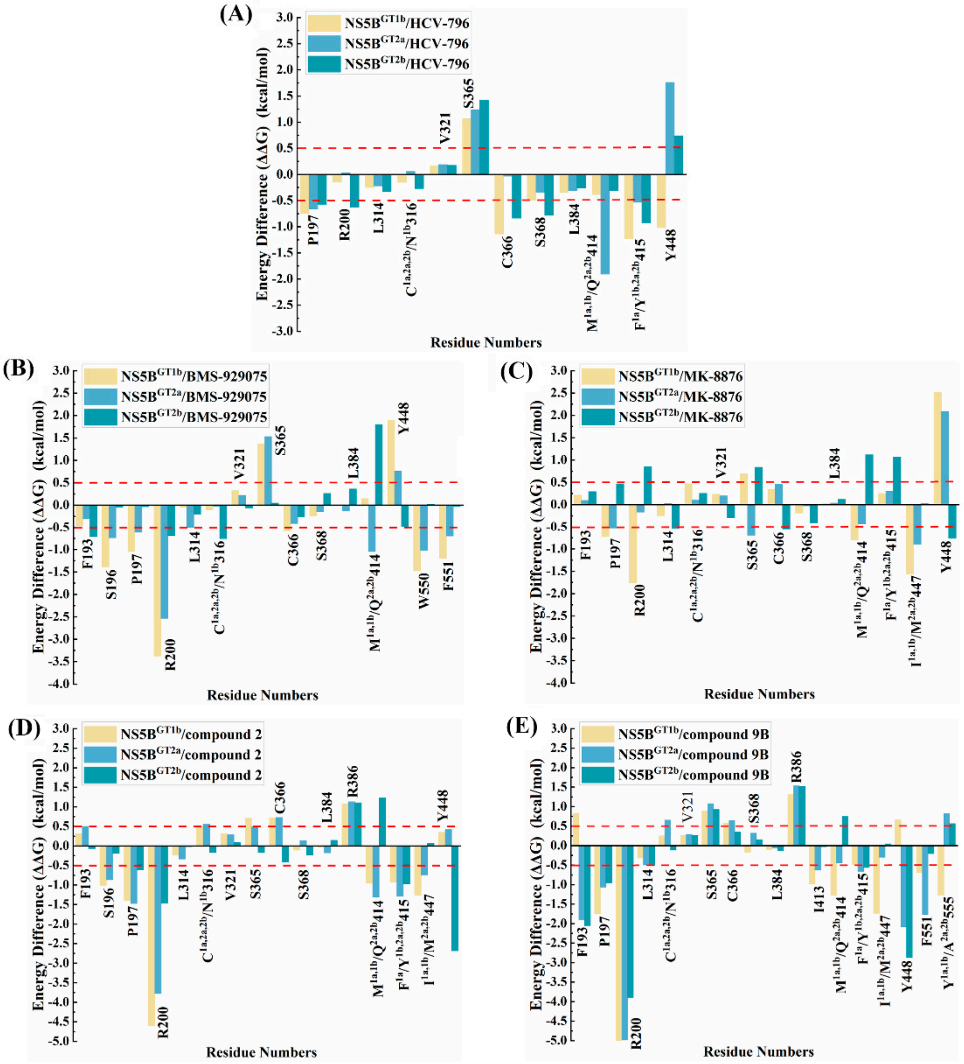


Figure S3. The energetic difference ($\Delta\Delta G=\Delta GNS5B^{GT1b/GT2a/GT2b}$/inhibitor - $\Delta GNS5B^{GT1a}$/inhibitor) spectra of the corresponding key residues between the NS5B$^{GT1a}$ system and the NS5B$^{GT1b}$ system, NS5B$^{GT2a}$ system, or NS5B$^{GT2b}$ system. (A) NS5B/HCV-796; (B) NS5B/BMS-929075; (C) NS5B/MK-8876; (D) NS5B/compound 2; (E) NS5B/compound 9B.

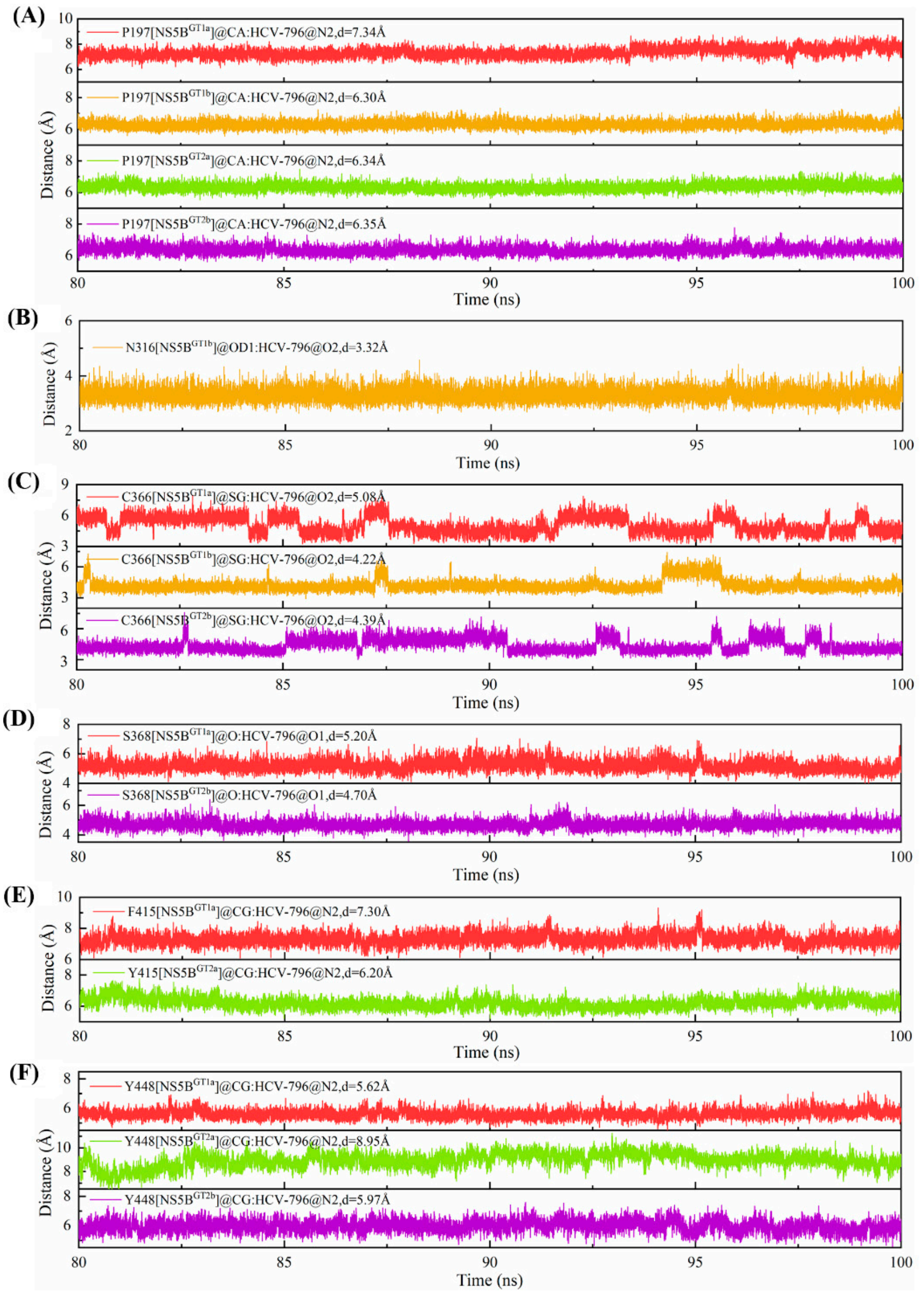


Figure S4. Distances between HCV-796 and residues during the last 20 ns MD trajectories.

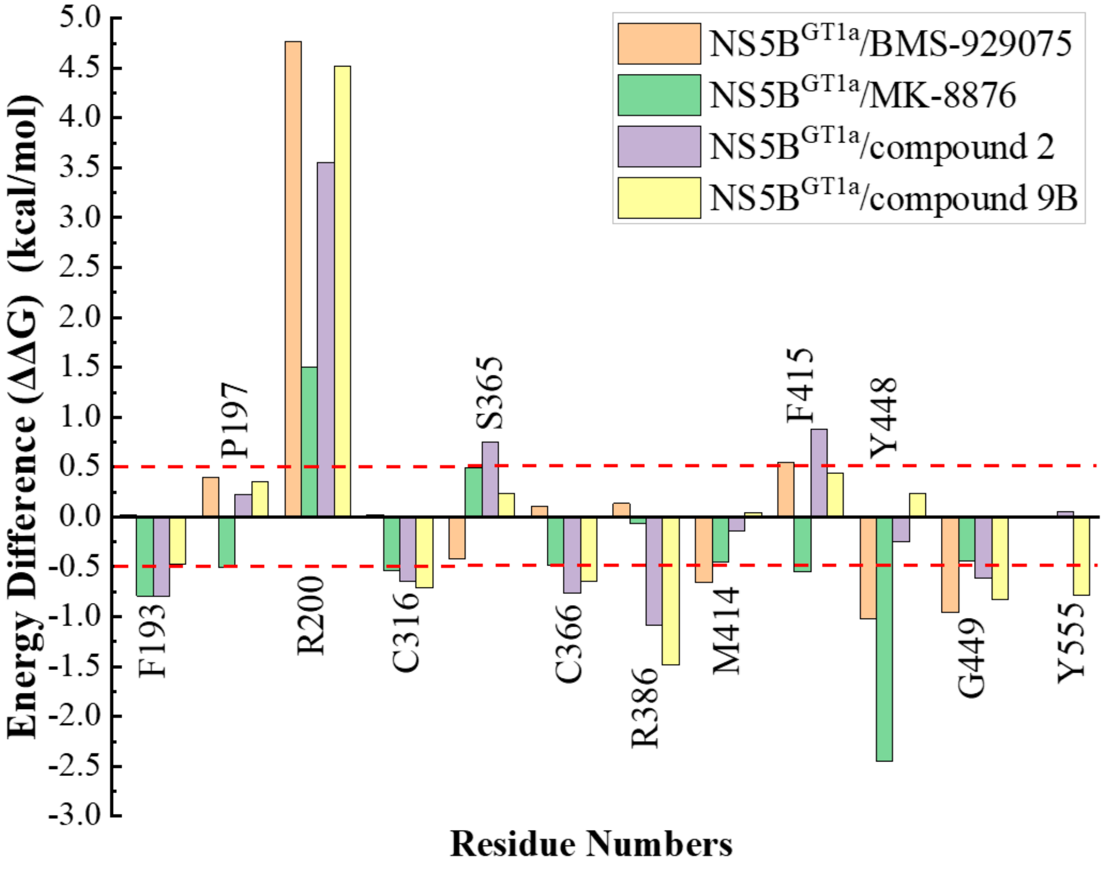


Figure S5. The energetic difference spectra between the NS5B$^{GT1a}$/HCV-796 system and the NS5B$^{GT1a}$/BMS-929075, NS5B$^{GT1a}$/MK-8876, NS5B$^{GT1a}$/compound 2 or NS5B$^{GT1a}$/compound 9B system ($\Delta\Delta G = \Delta G$NS5B$^{GT1a}$/(BMS-929075/MK-8876/compound 2/compound 9B) - $\Delta G$NS5B$^{GT1a}$/HCV-796).

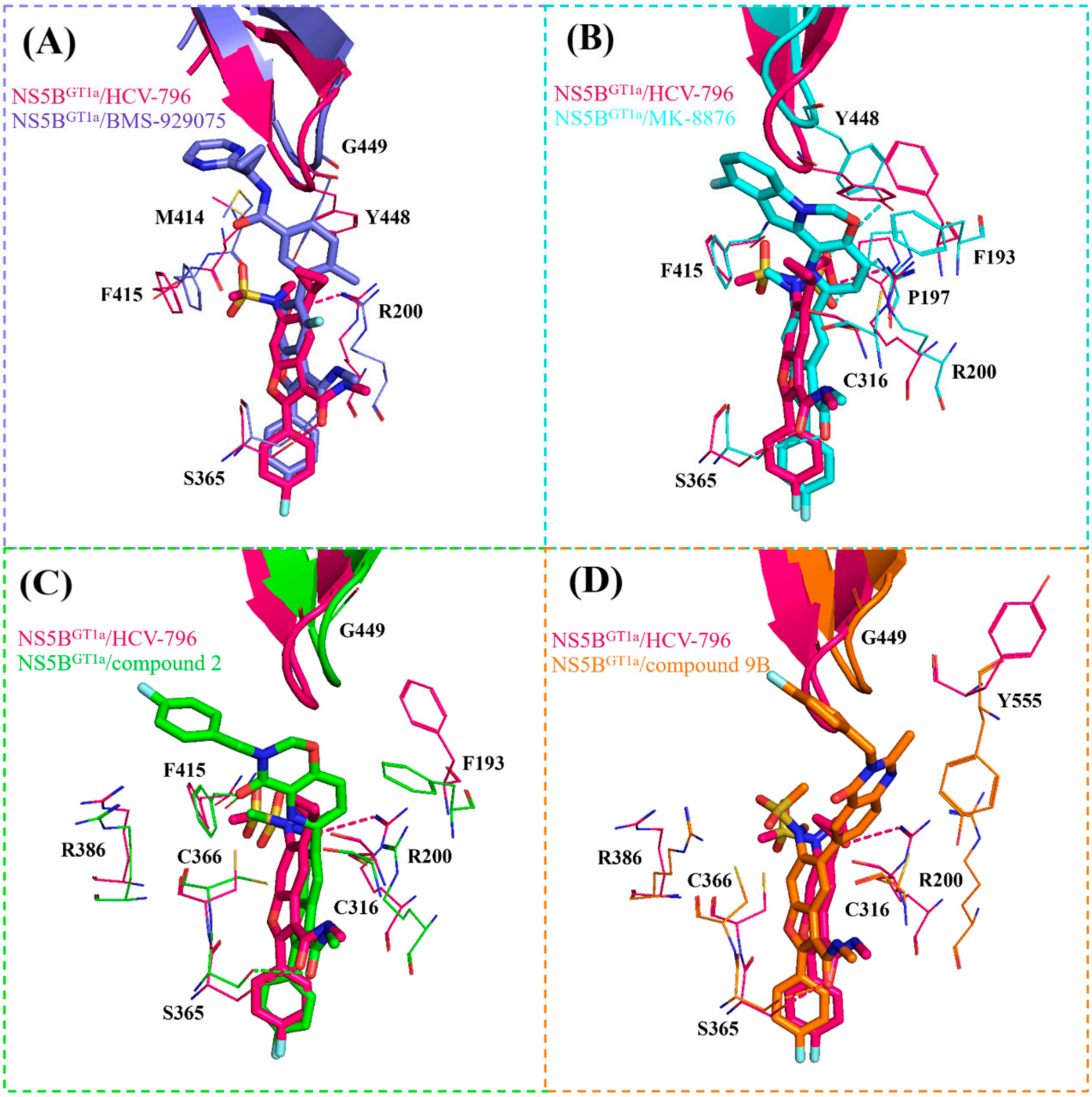


Figure S6. The binding modes of (A) NS5B$^{GT1a}$/HCV-796 versus NS5B$^{GT1a}$/BMS-929075, (B) NS5B$^{GT1a}$/HCV-796 versus NS5B$^{GT1a}$/MK-8876, (C) NS5B$^{GT1a}$/HCV-796 versus NS5B$^{GT1a}$/compound 2, and (D) NS5B$^{GT1a}$/HCV-796 versus NS5B$^{GT1a}$/compound 9B.

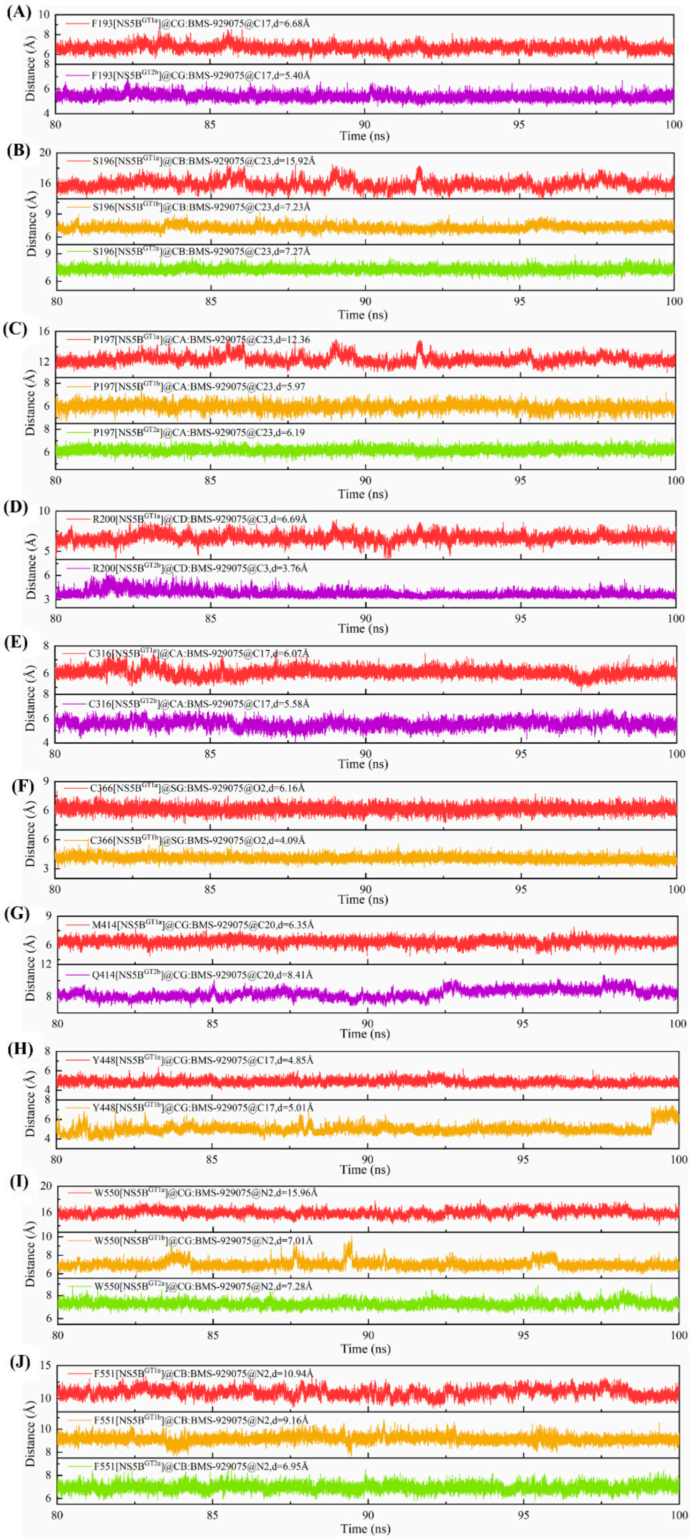


Figure S7. Distances between BMS-929075 and residues during the last 20 ns MD trajectories.

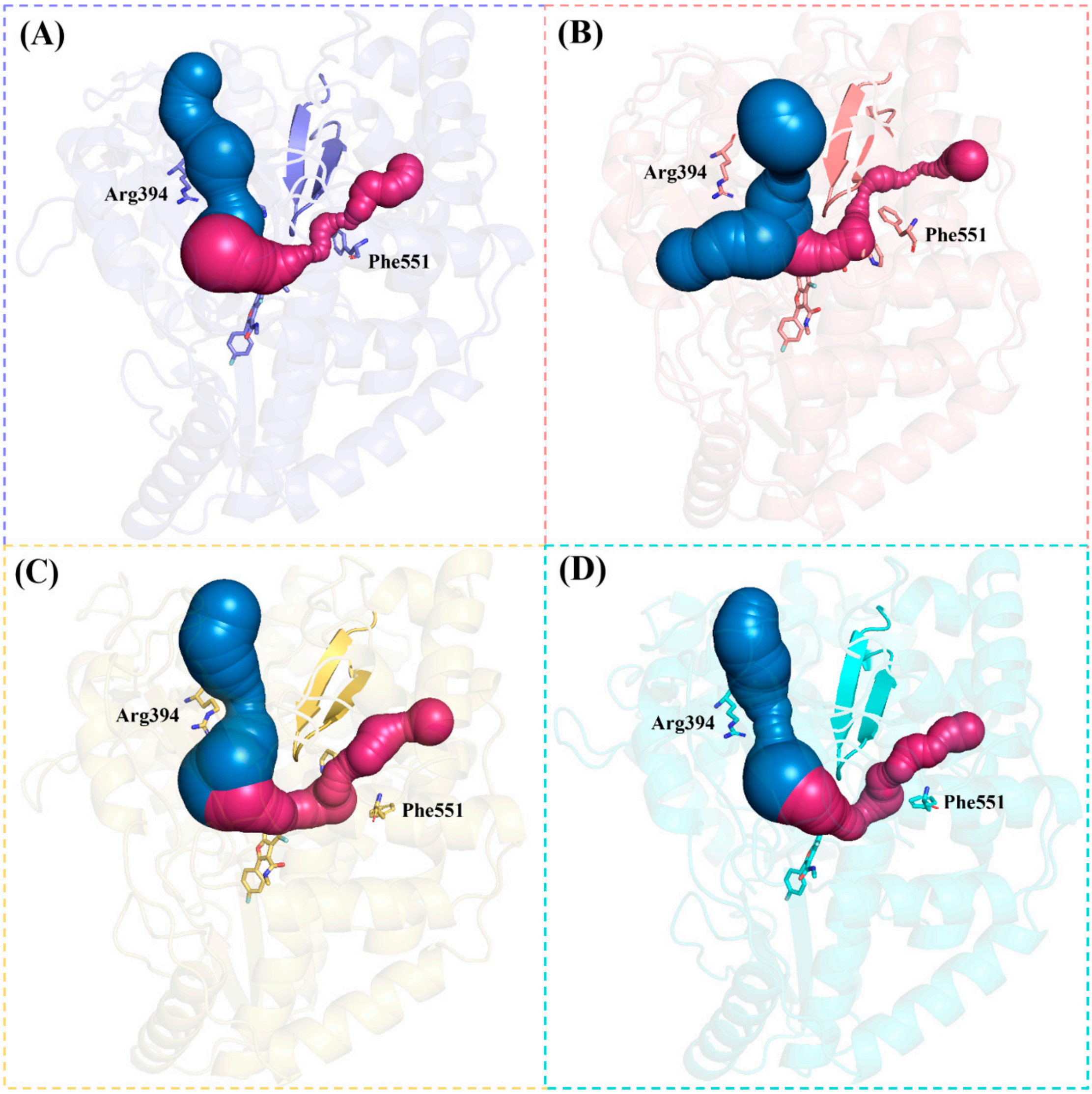


Figure S8. The tunnels represented by residues Arg394 (skyblue) and Phe551 (warmpink) for (A) NS5B$^{GT1a}$/BMS-929075 (slate), (B) NS5B$^{GT1b}$/BMS-929075 (salmon), (C) NS5B$^{GT2a}$/BMS-929075 (yellow orange) and (D) NS5B$^{GT2b}$/BMS-929075 (cyan).

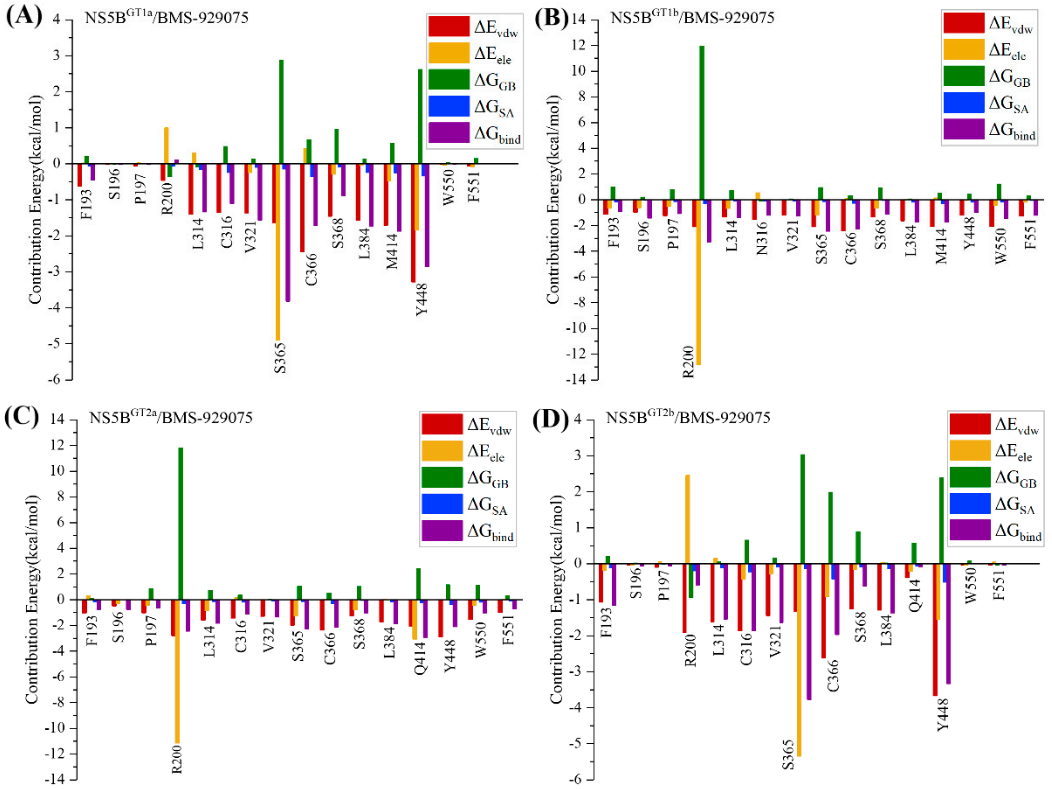


Figure S9. The individual energy term contributions of key residues to the binding free energies of (A) $NS5B^{GT1a}$/BMS-986139, (B) $NS5B^{GT1b}$/BMS-986139, (C) $NS5B^{GT2a}$/BMS-986139, and (D) $NS5B^{GT2b}$/BMS-986139 system.

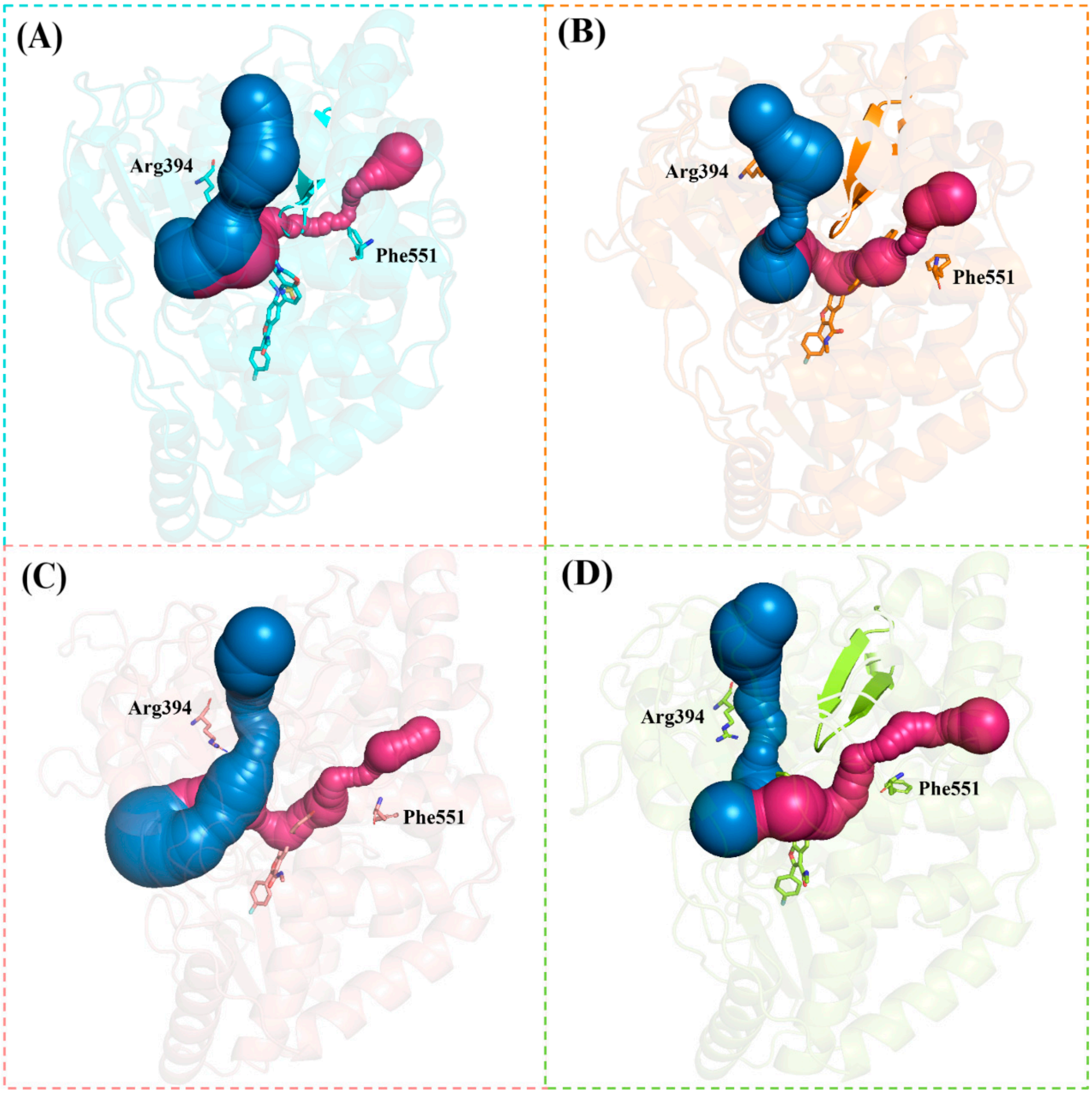


Figure S10. The tunnels represented by residues Arg394 (skyblue) and Phe551 (warmpink) for (A) $NS5B^{GT1a}$/MK-8876 (cyan), (B) $NS5B^{GT1b}$/MK-8876 (orange), (C) $NS5B^{GT2a}$/MK-8876 (salmon) and (D) $NS5B^{GT2b}$/MK-8876 (limon).

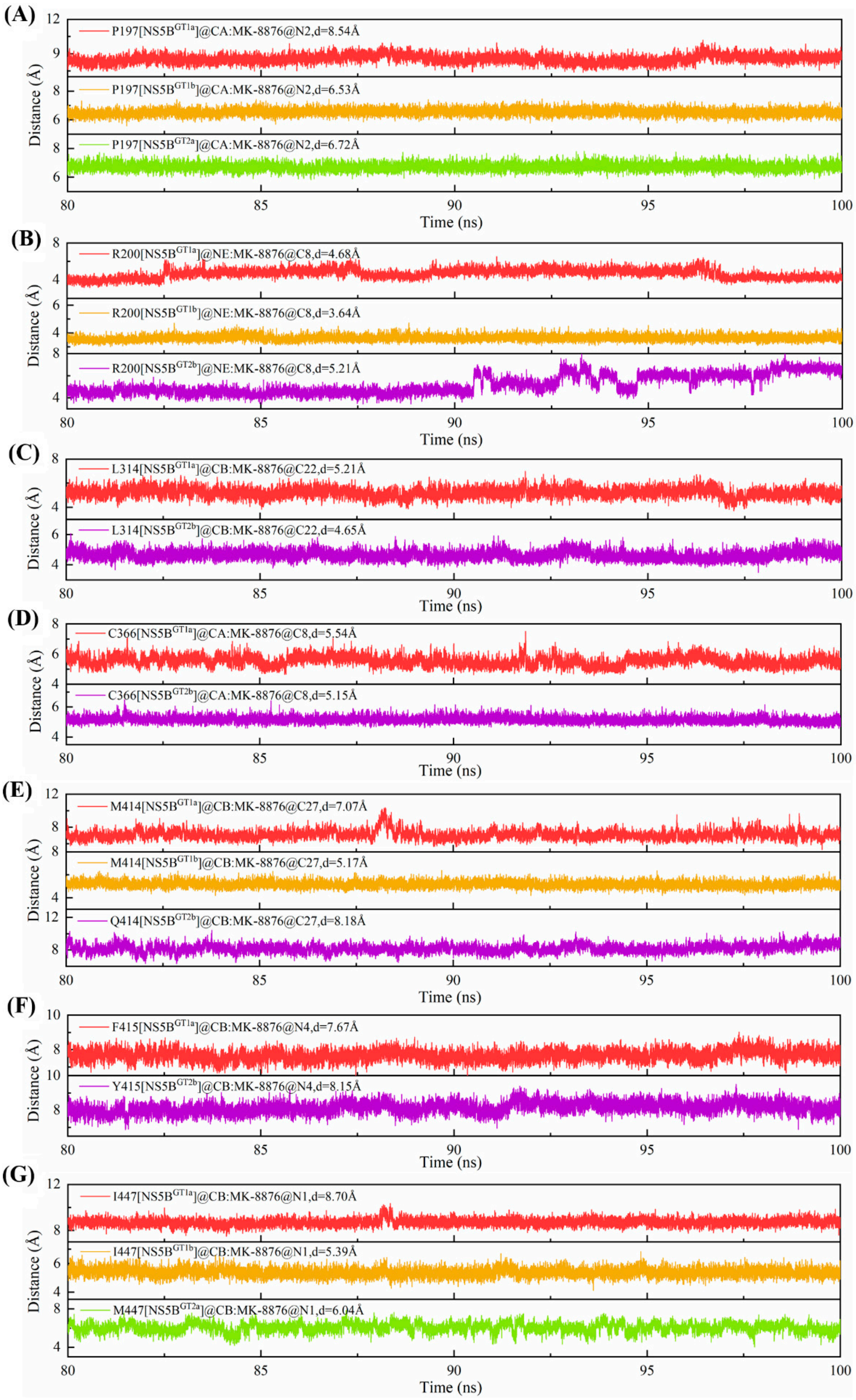


Figure S11. Distances between MK-8876 and residues during the last 20 ns MD trajectories.

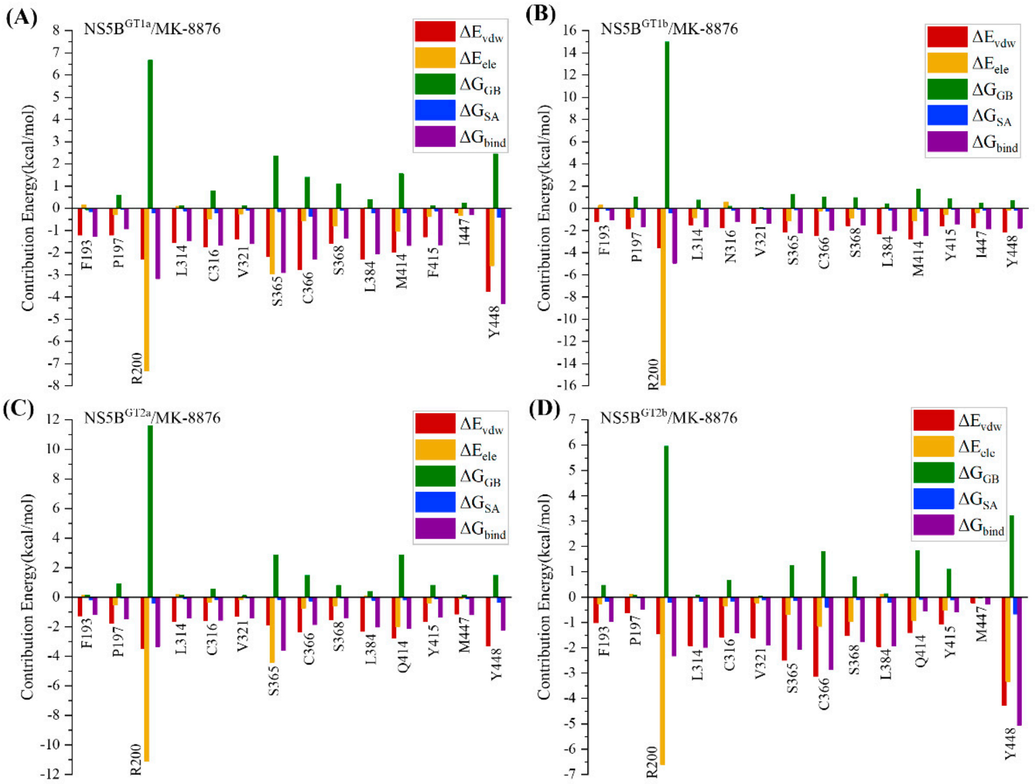


Figure S12. The individual energy term contributions of key residues to the binding free energies of (A) $NS5B^{GT1a}$/MK-8876, (B) $NS5B^{GT1b}$/MK-8876, (C) $NS5B^{GT2a}$/MK-8876, and (D) $NS5B^{GT2b}$/MK-8876 system.

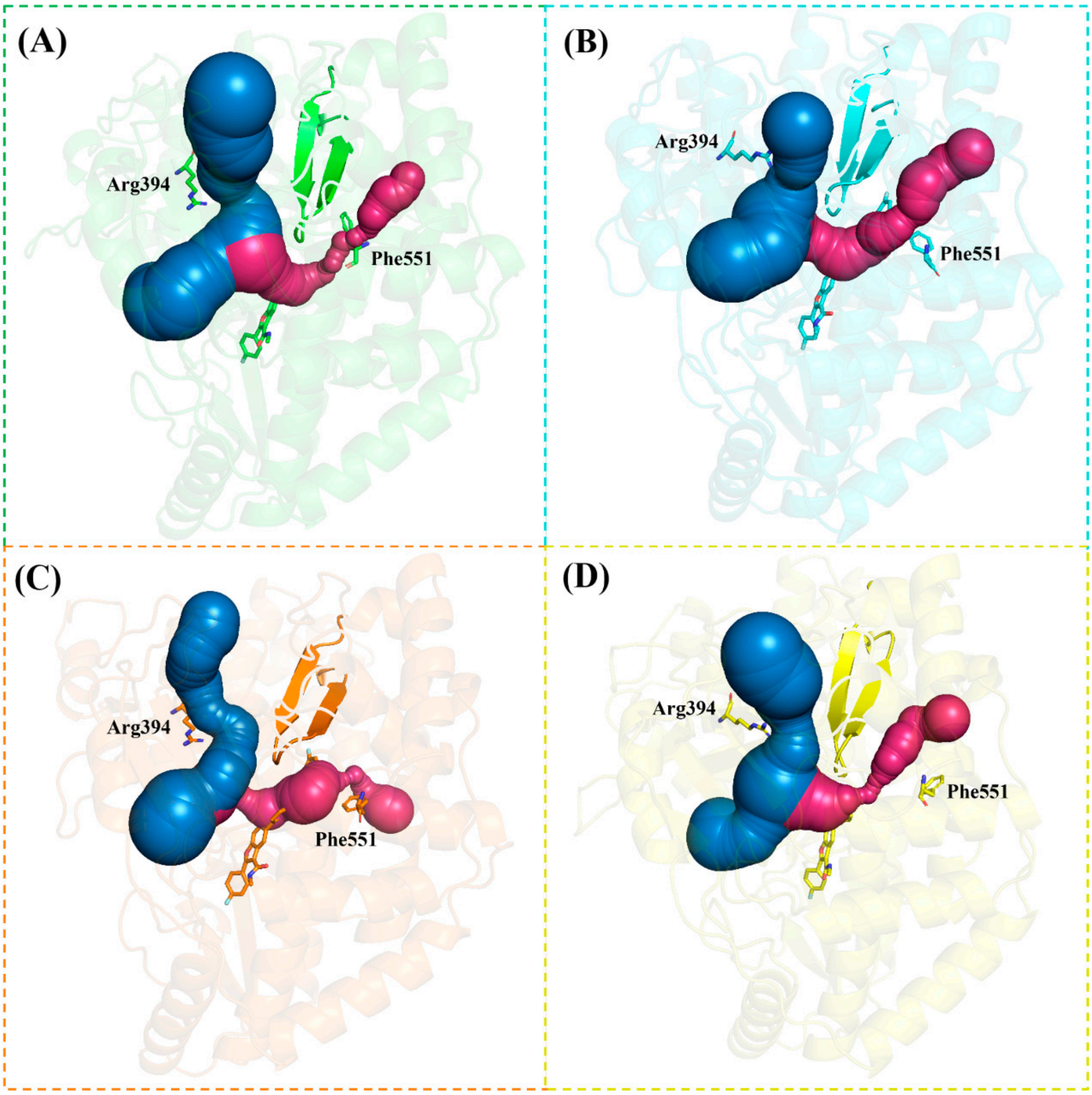


Figure S13. The tunnels represented by residues Arg394 (skyblue) and Phe551 (warmpink) for (A) NS5B$^{GT1a}$/compound 2 (green), (B) NS5B$^{GT1b}$/compound 2 (cyan), (C) NS5B$^{GT2a}$/compound 2 (orange) and (D) NS5B$^{GT2b}$/compound 2 (yellow).

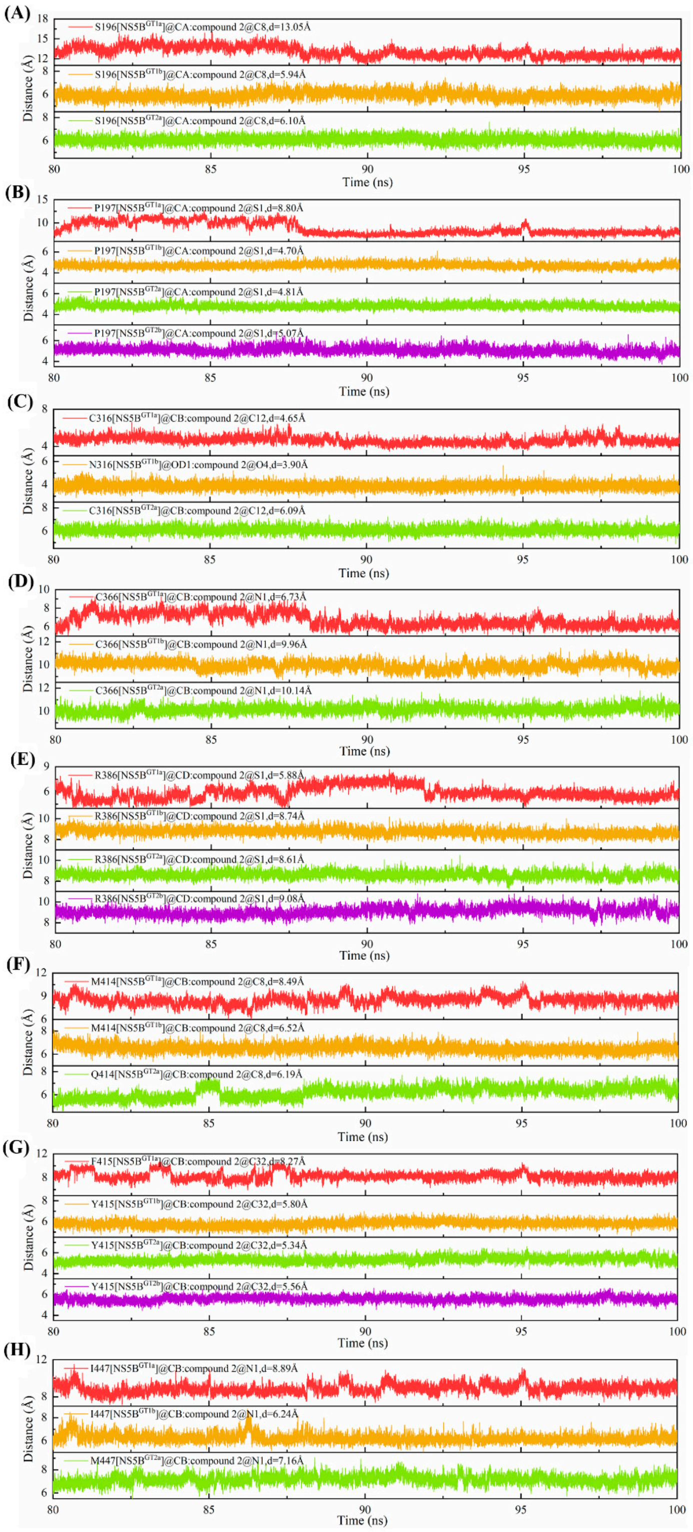


Figure S14. Distances between compound 2 and residues during the last 20 ns MD trajectories.

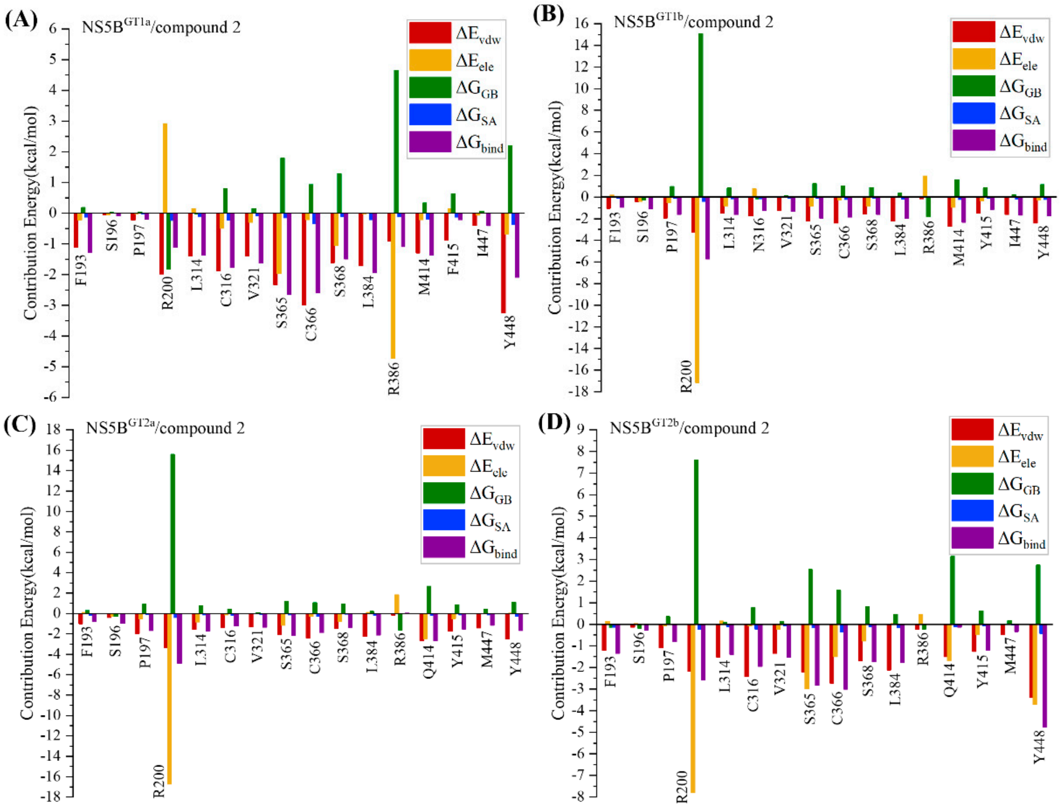


Figure S15. The individual energy term contributions of key residues to the binding free energies of (A) $NS5B^{GT1a}$/compound 2, (B) $NS5B^{GT1b}$/compound 2, (C) $NS5B^{GT2a}$/compound 2 and (D) $NS5B^{GT2b}$/compound 2 system.

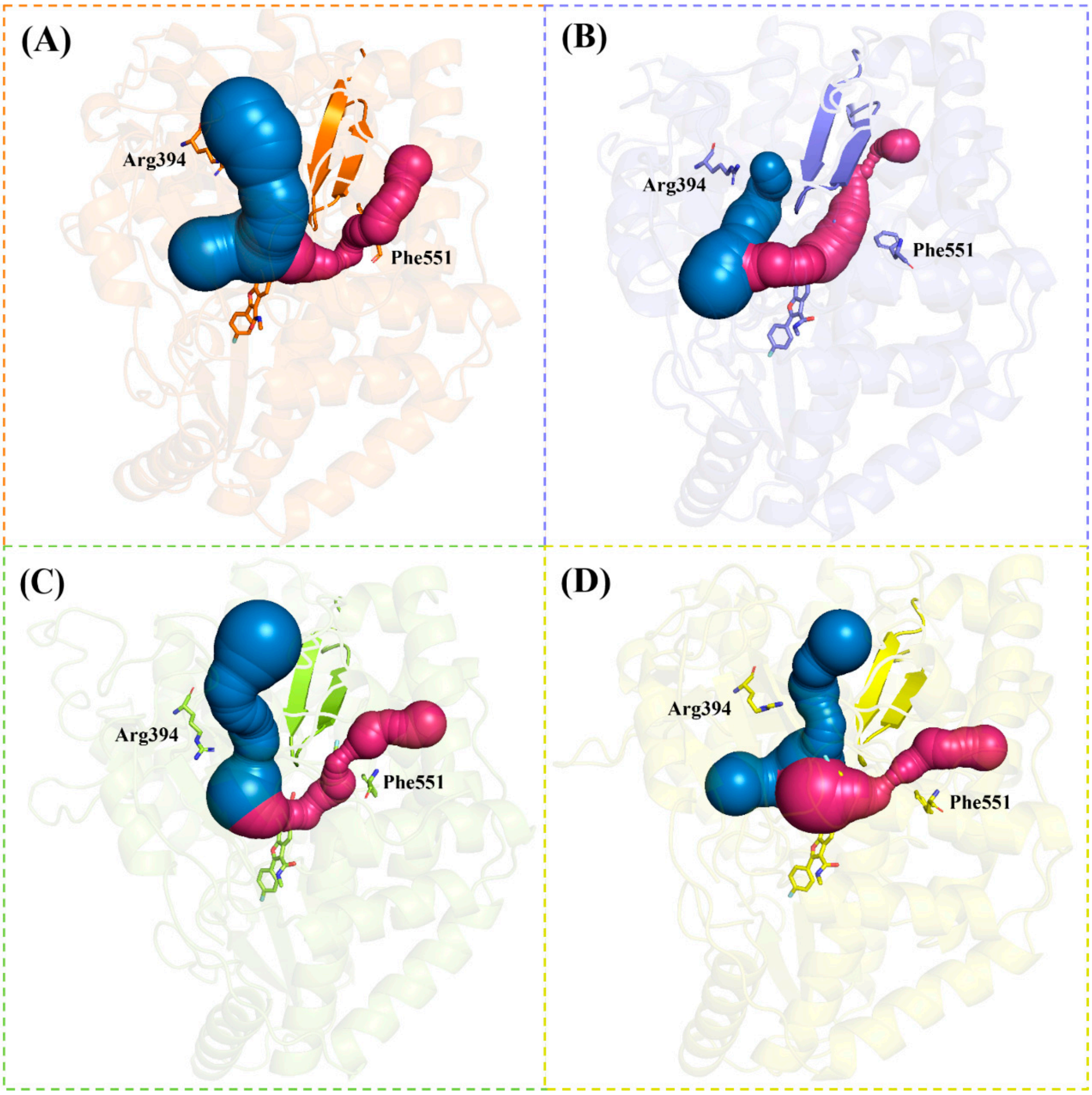


Figure S16. The tunnels represented by residues Arg394 (skyblue) and Phe551 (warmpink) for (A) NS5B$^{GT1a}$/compound 9B (orange), (B) NS5B$^{GT1b}$/compound 9B (slate), (C) NS5B$^{GT2a}$/compound 9B (limon) and (D) NS5B$^{GT2b}$/compound 9B (yellow).

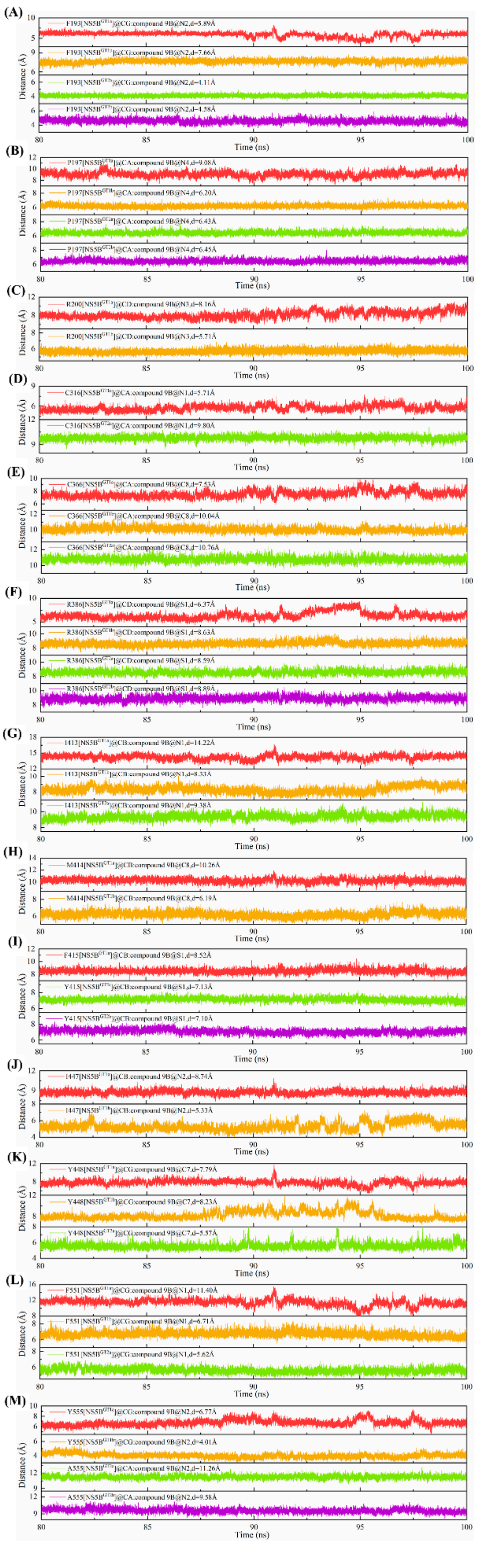

Figure S17. Distances between compound 9B and residues during the last 20 ns MD trajectories.

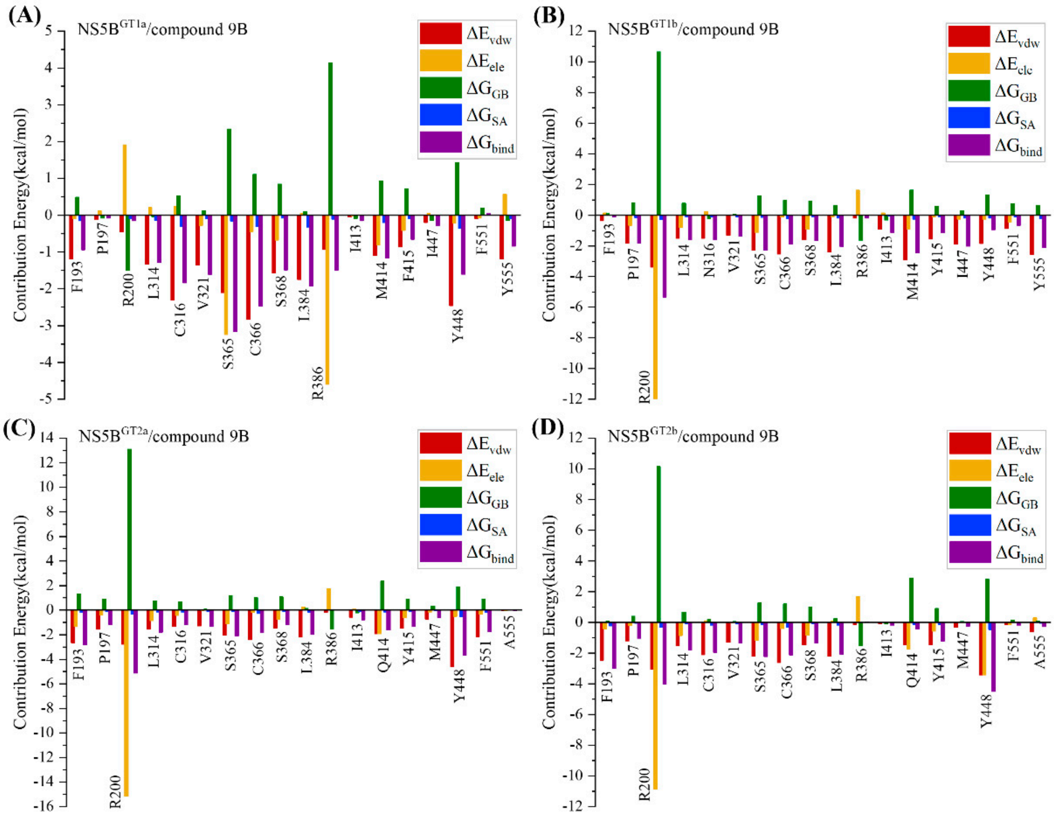


Figure S18. The individual energy term contributions of key residues to the binding free energies of (A) NS5B$^{GT1a}$/compound 9B, (B) NS5B$^{GT1b}$/compound 9B, (C) NS5B$^{GT2a}$/compound 9B and (D) NS5B$^{GT2b}$/compound 9B system.

## Summary of Common Residues Contributing to the Binding of Benzofuran Core Inhibitors

The benzofuran core inhibitors, including HCV-796, BMS-929075, MK-8876, compound 2, and compound 9B, exhibit significant pan-genotypic activity against various genotypes of the NS5B polymerase of the hepatitis C virus (HCV). Molecular simulations, including molecular docking, molecular dynamics (MD) simulations, MM/GBSA energy calculations, and adaptive steered molecular dynamics (ASMD) simulations, were used to elucidate their binding mechanisms. The results highlight several common residues that play crucial roles in the binding of these inhibitors to the NS5B polymerase across different genotypes (GT1a, 1b, 2a, and 2b). These key residues are predominantly located within the Palm II subdomain and its overlapping regions with Palm I and Palm III subdomains of the NS5B polymerase.

**Key Residues and Their Contributions**

1. Arg200: This residue is involved in significant interactions with all five inhibitors. It frequently forms hydrogen bonds and contributes to the electrostatic interactions, playing a critical role in stabilizing the inhibitor binding.
2. Leu314: Contributes mainly through van der Waals interactions, providing hydrophobic stability to the inhibitor binding.
3. Cys316/Asn316: Depending on the genotype, this residue contributes through hydrogen bonding and van der Waals interactions. Notably, Asn316 in GT1b forms additional hydrogen bonds due to its polar side chain.
4. Val321: Engages in hydrophobic interactions with the inhibitors, contributing to van der Waals forces that stabilize the binding.
5. Ser365: Frequently forms hydrogen bonds with the inhibitors, particularly with HCV-796 and MK-8876, indicating its importance in polar interactions.
6. Cys366: Contributes through both van der Waals and electrostatic interactions, enhancing the overall binding strength of the inhibitors.
7. Ser368: Forms hydrogen bonds with some inhibitors and contributes to the binding through polar interactions.

8. Leu384: Involved in hydrophobic interactions, contributing to the stability of the inhibitor binding through van der Waals forces.

9. Met414/Gln414: Depending on the genotype, this residue participates in hydrophobic and polar interactions, significantly affecting binding affinity.

10. Phe415/Tyr415: These residues engage in hydrophobic interactions and, in the case of Tyr415 in GT1b, additional hydrogen bonding, significantly enhancing binding strength.

11. Tyr448: Consistently forms hydrogen bonds with various inhibitors, contributing to both polar and hydrophobic interactions.

**Binding Modes and Interaction Patterns**

The binding of benzofuran core inhibitors is characterized by extensive interactions with residues in the Palm II subdomain and its overlapping regions with Palm I and Palm III subdomains. The interactions include hydrogen bonds, van der Waals forces, and electrostatic contributions. The van der Waals interactions, particularly with residues such as Leu314, Val321, and Leu384, are crucial for the stable binding of the inhibitors. Polar residues like Arg200, Ser365, and Tyr448 often form hydrogen bonds, further stabilizing the inhibitor-NS5B complex.

The detailed binding modes and interaction patterns elucidated by the simulations provide valuable insights for the design and optimization of novel benzofuran core inhibitors with enhanced pan-genotypic activity against HCV NS5B polymerase.

## Highlight of Differences in Target Residues or Compound Substituents for Designing More Efficient or Selective Compounds

The study on benzofuran core inhibitors reveals significant differences in target residues and compound substituents that could be leveraged to design more efficient and selective inhibitors against various genotypes of the NS5B polymerase. The key findings from the binding mode analyses and molecular simulations highlight several critical aspects:

**Differences in Target Residues**

1. **Cys316 vs. Asn316 (GT1a vs. GT1b)**:

   **Cys316 (GT1a)**: Primarily involved in van der Waals interactions.

   **Asn316 (GT1b)**: Engages in additional hydrogen bonding due to its polar side chain, enhancing binding strength.

2. **Phe415 vs. Tyr415 (GT1a vs. GT1b/GT2a/GT2b)**:

   **Phe415 (GT1a)**: Contributes mainly through hydrophobic interactions.

   **Tyr415 (GT1b/GT2a/GT2b)**: Forms stronger hydrogen bonds with inhibitors, significantly enhancing binding affinity.

3. **Met414 vs. Gln414 (GT1a/GT1b vs. GT2a/GT2b)**:

   **Met414 (GT1a/GT1b)**: Participates in hydrophobic interactions.

   **Gln414 (GT2a/GT2b)**: Involved in polar interactions, contributing to a different binding dynamic.

4. **Tyr448**:

   **GT2b**: Forms stronger hydrogen bonds compared to other genotypes, suggesting genotype-specific interactions that could be targeted for selective inhibition.

**Differences in Compound Substituents**

1. **C5 Position Substituents**:

   **Longer Substituents (e.g., BMS-929075, MK-8876, compound 2, compound 9B)**: Exhibit different binding patterns between GT1a/2b and GT1b/2a, indicating that modifications at the C5 position can be tailored to enhance

selectivity and potency across different genotypes.

2. **Para-Fluorophenyl Groups at C2 Position**:

**Interaction Variations**: The para-fluorophenyl groups at the C2 position interact differently with residues across various genotypes, suggesting that modifying this substituent could optimize binding affinity and specificity.

**Design Implications**

- **Targeting Polar Residues**: Enhancing interactions with polar residues like Asn316 and Gln414 can improve binding strength in genotypes where these residues are present, such as GT1b and GT2b.
- **Substituent Optimization**: Modifying the substituents at the C5 and C2 positions to enhance specific interactions with key residues like Tyr415 and Tyr448 can lead to more selective inhibitors.
- **Hydrophobic vs. Polar Balancing**: Balancing hydrophobic and polar interactions by adjusting substituents can optimize binding across different genotypes, as seen with the varied contributions of residues like Cys316 and Asn316.

**Example Binding Modes**

- **HCV-796**: Forms multiple hydrogen bonds and hydrophobic interactions, but its hepatotoxicity suggests the need for substituent modifications to reduce off-target effects.
- **BMS-929075 and MK-8876**: Show strong binding across multiple genotypes, indicating that their structural features, such as longer C5 substituents, are beneficial for pan-genotypic activity.

These insights provide a foundation for designing novel benzofuran core inhibitors with improved efficiency and selectivity by focusing on the differences in target residues and optimizing compound substituents accordingly.
